# Refractive Error and Eye Health: An Umbrella Review of Meta-Analyses

**DOI:** 10.3389/fmed.2021.759767

**Published:** 2021-11-04

**Authors:** Yin-hao Wang, Chen Huang, Yu-lin Tseng, Jing Zhong, Xue-min Li

**Affiliations:** ^1^Department of Ophthalmology, Peking University Third Hospital, Beijing, China; ^2^Beijing Key Laboratory of Restoration of Damaged Ocular Nerve, Peking University Third Hospital, Beijing, China; ^3^Center of Basic Medical Research, Peking University Third Hospital, Beijing, China

**Keywords:** refractive error, umbrella review, eye health, glaucoma, cataract, age-related macular degeneration (AMD), diabetic retinopathy, strabismus

## Abstract

**Purpose:** To explore the associations between refractive errors and multiple eye health outcomes.

**Methods:** This is an umbrella review based on systematic reviews with meta-analyses. In our study, refractive errors included myopia, hyperopia, astigmatism, and anisometropia. We reconducted the meta-analyses whose primary data were available in sufficient detail by random effect model. Heterogeneity was assessed by *I*^2^. The main outcomes included myopic macular degeneration (MMD), retinal detachment (RD), cataract, open-angle glaucoma (OAG), strabismus, age-related macular degeneration (AMD), and diabetic retinopathy (DR).

**Results:** Myopia was associated with increased risk of MMD (relative risk = 102.11, 95% CI 52.6–198.22), RD (3.45, 1.08–11.00), nuclear cataract (2.15, 1.53–3.03), posterior subcapsular (PSC) cataract (1.74, 1.41–2.15), OAG (1.95, 1.74–2.19), exotropia (5.23, 2.26–12.09), but decreased risk of DR (0.83, 0.66–1.04), and early AMD (0.80, 0.67–0.94). From mild-to-high myopia, the association strengthened for MMD, RD, nuclear cataract, PSC cataract, OAG, and DR. Hyperopia was associated with an increased risk of early AMD (1.09, 1.01–1.18) and esotropia (22.94, 10.20–51.62). Astigmatism and anisometropia were associated with increased risk of both exotropia and esotropia.

**Conclusions:** Myopia, especially high myopia, demonstrated the highest risk for eye health outcomes, such as MMD, RD, OAG, nuclear and PSC cataracts, and exotropia. However, myopia was associated with a lower risk of early AMD and DR. Individuals with hyperopia are more likely to suffer early AMD and esotropia. Astigmatism and anisometropia predispose to strabismus. A lot of research studies on the mechanism of the associations are needed.

**Systematic Review Registration:** https://www.crd.york.ac.uk/PROSPERO/display_record.php?RecordID=239744; identifier: 239744

## Introduction

In refractive error, parallel light cannot focus on the fovea to form a clear image after passing through the ocular refractive system; refractive errors include myopia, hyperopia, astigmatism, anisometropia, and presbyopia (1). In 2010, uncorrected refractive error was the leading cause of vision impairment and the second leading cause of blindness worldwide, affecting 108 million people (2). Additionally, ametropia-associated ocular diseases, including myopic macular degeneration (MMD), cataract, glaucoma, diabetic retinopathy (DR), and age-related macular degeneration (AMD), also greatly impair vision (3, 4). Eyes with a refractive error have a changed intraocular environment, globe structure, and accommodation, which can influence multiple aspects of eye health (5–7). Several studies have explored the associations between refractive errors and eye health. However, a comprehensive and systematic summary of these associations is lacking, which limits the thorough understanding of refractive errors.

High-quality evidence is needed to demonstrate the associations between refractive errors and eye health. In the past, systematic reviews and meta-analyses were widely considered the highest level of evidence. However, with numerous systematic reviews and meta-analyses available, it is difficult to distinguish priority in most cases. Thus, it may be very useful to perform systematic reviews of systematic reviews, i.e., umbrella reviews (8). Umbrella reviews allow a higher-level synthesis of the evidence and better recognition of the uncertainties, biases, and knowledge gaps (9). Therefore, we aimed to conduct an umbrella review to explore a broad range of eye health outcomes in patients with refractive error in order to provide guidance for future research.

## Methods

We registered this umbrella review on PROSPERO (CRD42021239744). We reviewed refractive errors and multiple eye health outcomes by systematically searching for meta-analyses. In this review, refractive errors included myopia, hyperopia, astigmatism, and anisometropia. Presbyopia was excluded because we considered that presbyopia is a physiological lens-aging process. Specifically, we excluded systematic reviews without meta-analyses.

### Search Strategy and Selection Criteria

Two researchers (YW and YT) independently searched PubMed, Embase, the Cochrane Library, China National Knowledge Internet, China Biology Medicine, and the WANFANG database from inception to February 21, 2021, for meta-analyses of observational or interventional studies that analyzed the association between refractive errors and eye health outcomes. We used the following medical subject heading (MeSH) terms and keywords in the search: (“refractive error,” “ametropia,” “hyperopia,” “myopia,” “astigmatism,” “anisometropia”, or their synonyms) and (“systematic review” or “meta-analysis”). Two researchers (YW and YT) independently screened the titles and abstracts and then reviewed the full text of the selected articles for eligibility. When differences occurred, a third researcher (CH) was consulted. We also conducted a manual search of the references cited in eligible articles.

An article was eligible if it (1) provided odds ratios, risk ratios, hazard ratios, and weighted mean differences, or raw data that could be used to calculate the relative risks or mean difference; (2) was a systematic review with a meta-analysis of observational population-based (cohort, case-control, and cross-sectional) and interventional studies (randomized controlled trial [RCT]); and (3) investigated the association between refractive error and eye health outcomes. There were no language limitations. If an article contained several meta-analyses, all meta-analyses were included separately. For the same outcome compared in the same group (e.g., two articles compared the risk of AMD in myopia vs. emmetropia) by multiple articles, we chose only one meta-analysis for each exposure to avoid the inclusion of duplicate studies. The following criteria were used: when the primary articles did not overlap or partially overlapped, we chose the meta-analysis with the largest number of studies, and when the primary studies completely overlapped, we selected the review with the highest quality.

### Data Extraction and Statistical Analysis

Authors YW and YT independently extracted data from eligible systematic reviews and meta-analyses. A third investigator (CH) resolved any difference in the extracted data between the two researchers. For each meta-analysis, the following details were collected: first author, journal, year of publication, populations, number of studies, study design, funding sources, outcome(s) of interest, type(s) of refractive error, relative risks or mean difference and corresponding CIs, the measure of association, numbers of events and non-events, follow-up years (cohort studies), and type of effect model (random or fixed). We also extracted estimates of the proportion of variance (*I*^2^) and measures of publication bias.

We did not review the original studies of the published systematic reviews and meta-analyses. When the primary data were available in sufficient detail by the systematic reviews and meta-analyses, we reanalyzed each meta-analysis of the extracted data (Supplementary Material 3). A random-effects model (DerSimonian-Laird method) was used to conduct the meta-analyses. When the existed systematic reviews and meta-analyses did not provide the original data, we used summary data as extracted from the meta-analysis article directly. In this case, we obtained the measure of heterogeneity or publication bias, if any was available. We first selected multivariable-adjusted relative risks and used the crude relative risks if no adjusted relative risks were provided. We directly extracted the crude relative risks from meta-analyses or calculated them based on exposure and outcome data. We assessed the heterogeneity of each meta-analysis through the *I*^2^ statistic, with *I*^2^ > 50% indicating moderate-to-severe heterogeneity. If the primary data in the meta-analyses were insufficient for reanalysis, we used the extracted *I*^2^ values and effects models. Additionally, we produced estimates of publication bias using Egger's test for the studies that included more than 10 studies (10). A *P*-value of < 0.1 was considered significant for Egger's test. When a meta-analysis included cross-sectional, case-control, and cohort studies, we separately reanalyzed the cross-sectional and case-control studies, the cohort studies, and the combination of all three study designs. Cohort studies and RCTs are a higher form of evidence, and their relative risks can be hazard ratios, risk ratios, or odds ratios. Therefore, we extracted cohort studies or RCTs to obtain relative risks of incident events and used case-control or cross-sectional studies to calculate the relative risks of prevalent events. The statistical analysis was conducted by Stata/SE 14.0 for Windows (StataCorp LP, College Station, TX, USA).

### Assessment of Methodological Quality and Quality of Evidence

Two authors (YT and JZ) independently evaluated the eligible meta-analyses. Disagreements were resolved by consensus. We assessed the methodological quality of the meta-analyses through A Measurement Tool to Assess Systematic Reviews (AMSTAR), which has been proven to be reliable and valid for systematic reviews based on both interventional and observational studies (11, 12). The AMSTAR tool includes 11 items, with each item having four answers (“cannot answer,” “yes,” “no,” and “not applicable”). We awarded each “yes” item 1 point and summed these to calculate a total score. We used the Grading of Recommendations, Assessment, Development, and Evaluation (GRADE) working group classification to assess the quality of evidence for each outcome included in the umbrella review. The quality of evidence is categorized into “high,” “moderate,” “low,” or “very low” quality. High quality stands for further research is very unlikely to change our confidence in the estimate of effect; moderate quality: further research is likely to have an important impact on our confidence in the estimate of effect and may change the estimate; low quality: further research is very likely to have an important impact on our confidence in the estimate of the effect and is likely to change the estimate; and very low quality: we are very uncertain about the estimate. An evidence map was plotted according to the quality of the evidence.

## Results

**Figure 1:**
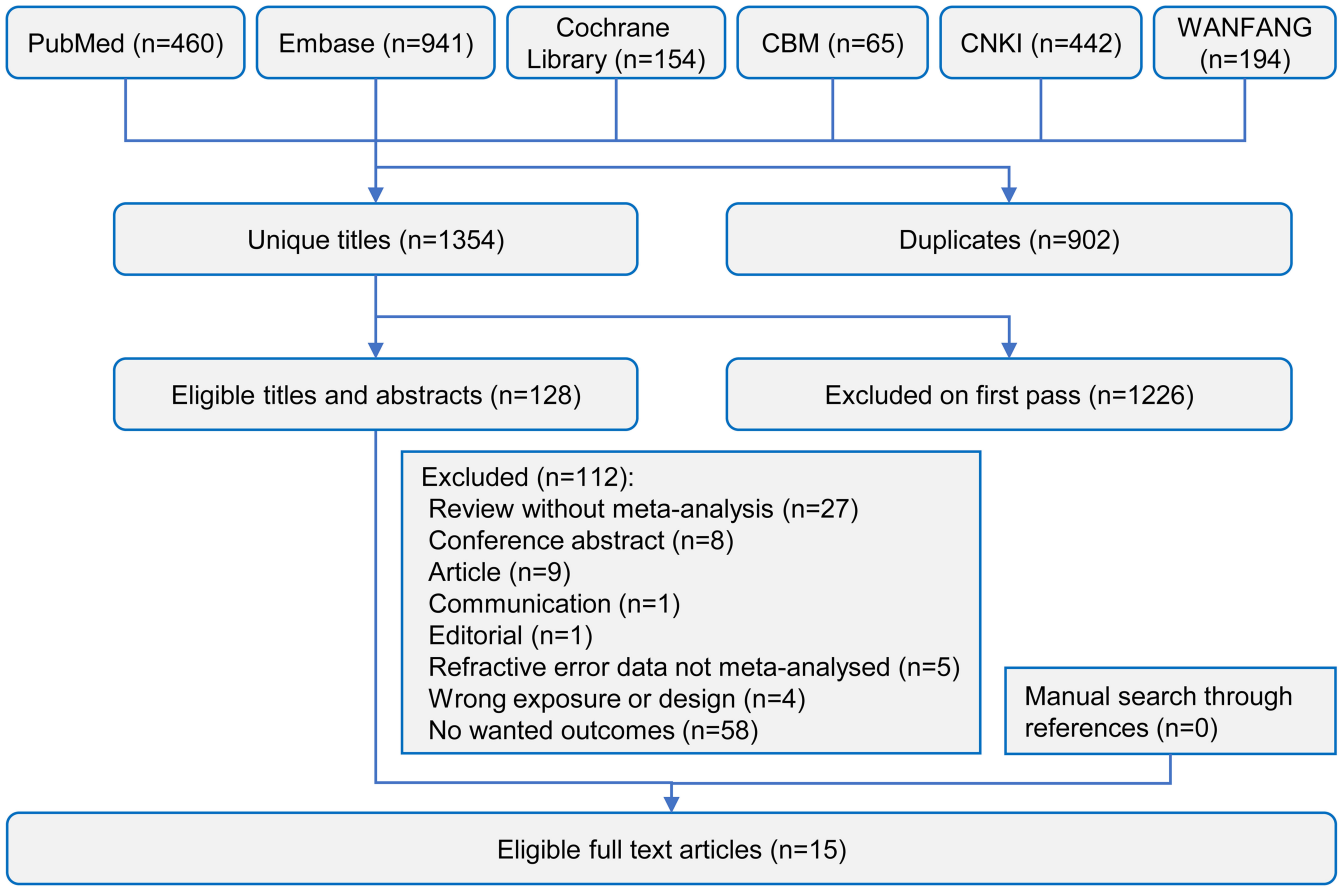
Flowchart of the study selection for the meta-analysis and systematic review.

Figure 1 shows the process of the systematic search and selection of eligible articles. The study retrieved 2,256 records through the literature search. After reviewing the title and abstract, we included 128 articles for full-text review. Finally, the study included 15 systematic reviews with 18 unique eye outcomes comprising 84 meta-analyses (13–27). We separated 27 of the 84 meta-analyses by study type and re-analyzed them. Supplementary Table 1 shows the characteristics of the included studies. Only one study included one RCT in the meta-analysis (22), while the other studies were observational studies.

**Figure 2:**
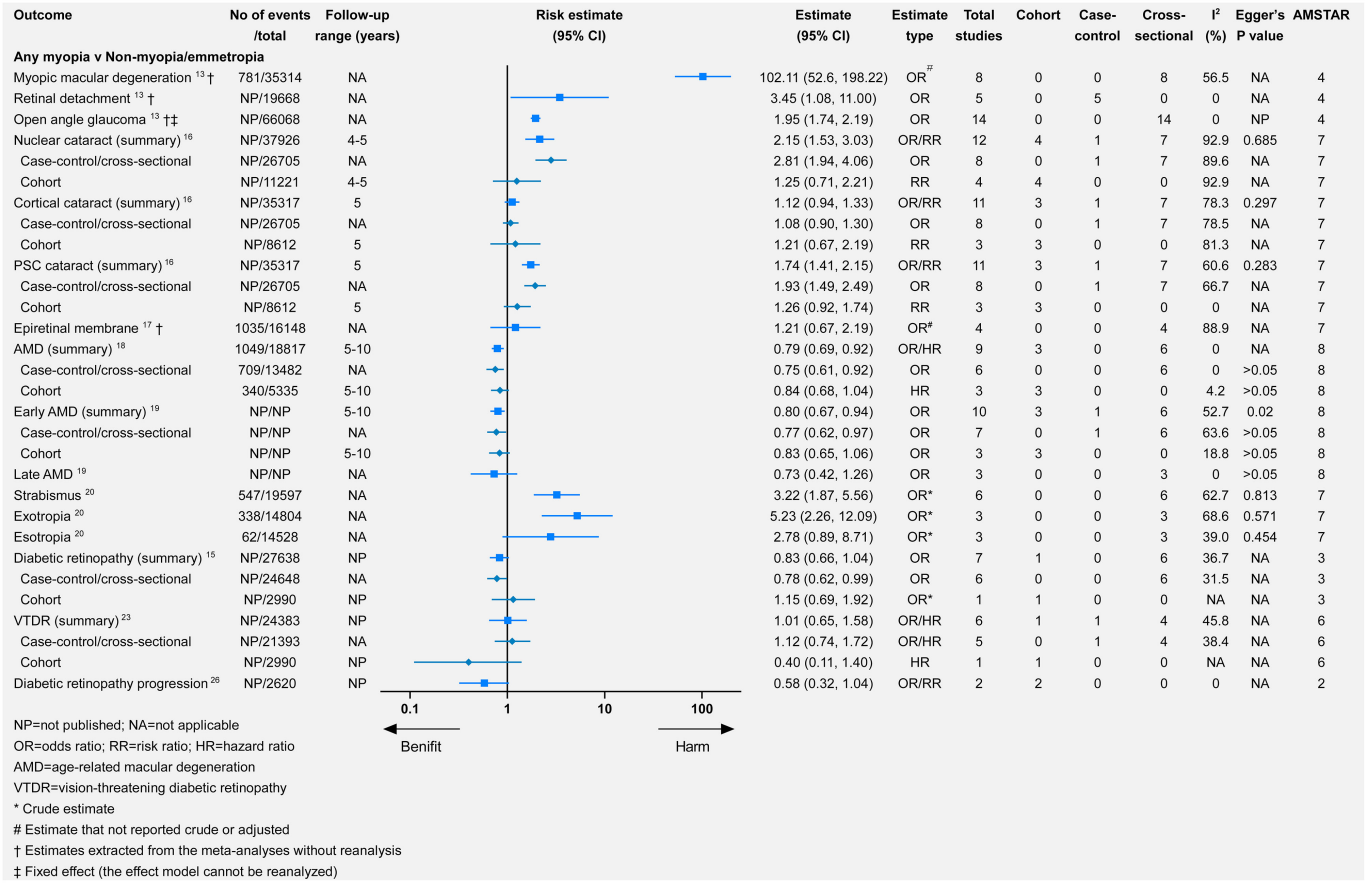
Associations of any myopia with multiple eye health outcomes.

Myopia was the most commonly studied refractive error, followed by hyperopia. Only one study investigated the association between astigmatism or anisometropia and eye health outcomes (20). The study categorized myopia into mild, moderate, or high myopia. Comparing any myopia with no myopia or emmetropia (Figure 2), there was significance for harmful associations with seven outcomes and for beneficial associations with two outcomes. Seven outcomes were not significantly associated with any myopia. For the comparison of mild, moderate, and high myopia with no myopia or emmetropia, 5, 4, and 5 outcomes demonstrated significantly harmful associations, respectively, whereas only 1, 1, and 0 outcomes showed significant beneficial associations. Additionally, we assessed the influences of axial length (per millimeter increase) and spherical equivalent (SE, per D increase) for AMD, early AMD, late AMD, DR, and vision-threatening DR (VTDR). In the comparison of hyperopia with emmetropia, there were significantly harmful associations with three outcomes, and for beneficial associations with one outcome, there were harmful but non-significant associations with the four remaining outcomes. Finally, for both astigmatism and anisometropia, there were significantly harmful associations with all three outcomes.

### Myopic Macular Degeneration

**Figure 3:**
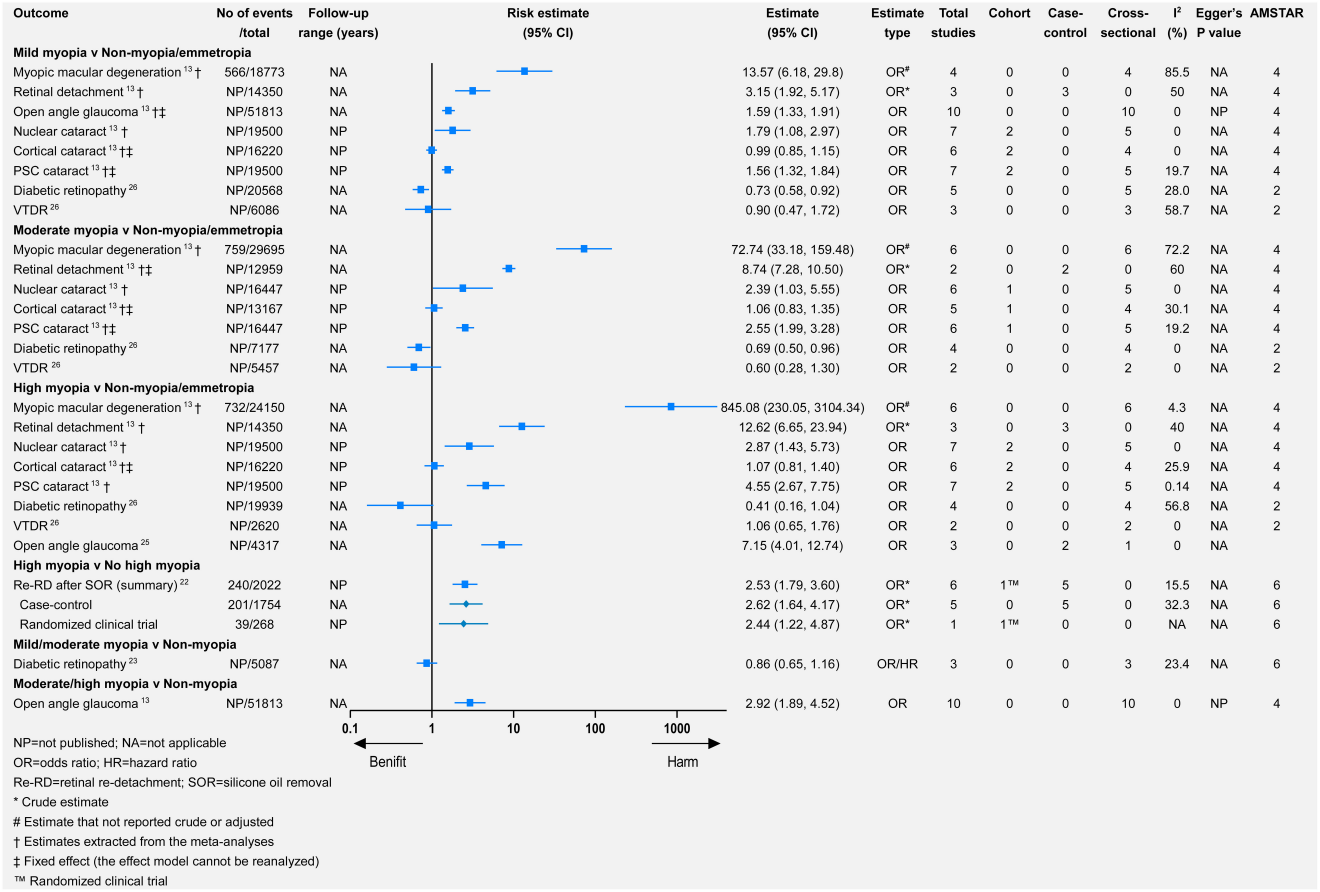
Associations of different myopia types with multiple eye health outcomes.

**Figure 4:**
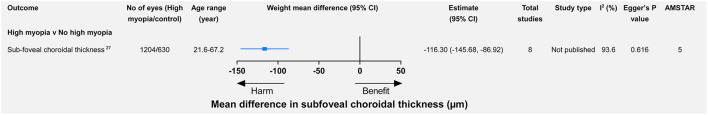
High myopia vs. non-high myopia and changes (mean difference) in subfoveal choroidal thickness.

Myopic macular degeneration was defined slightly differently across studies (see Supplementary Table 1). Compared with non-myopia, myopia was associated with a higher risk of MMD (relative risk = 102.11, 95% CI 52.6–198.22) (13). From the mild-to-high myopia population, the risk of MMD increased from 13.57 to 845.08 times that of the non-myopia population (Figure 3). Wang et al. meta-analyzed the difference in subfoveal choroidal thickness between high myopia and non-high myopia and reported that high myopia had a significantly thinner subfoveal choroidal thickness (Figure 4).

### Retinal Detachment

Myopia was associated with a significantly higher risk of retinal detachment (RD) (relative risk = 3.45, 95% CI 1.08–11.00) (13). The annual incidence of RD increased from 3 in 100,000 persons with hyperopia (>0 D) to 102 in 100,000 persons with high myopia (< −5 D) (28). Likewise, from mild-to-high myopia, the risk of RD increased (relative risk = 3.15, 95% CI 1.92–5.17 for mild myopia; relative risk = 8.74, 95% CI 7.28–10.50 for moderate myopia; relative risk = 12.62, 95% CI 6.65–23.94 for high myopia). Additionally, compared with non-high myopia, high myopia promoted the risk of retinal (re-RD) after silicone oil removal (relative risk = 2.53, 95% CI 1.79–3.60).

### Open-Angle Glaucoma

The most frequent definitions of open-angle glaucoma (OAG) were based on glaucomatous visual field loss and optic disc abnormality (13, 21, 24, 25). Myopia was significantly associated with a higher risk of OAG (relative risk = 1.95, 95% CI 1.74–2.19) (13), which was consistent in other meta-analyses (21, 24, 25). Moderate/high myopia had a higher risk of OAG than mild myopia (Figure 3). Xiang et al. stratified the Chinese myopia patient and reported that high myopia was significantly associated with a higher risk of non-high myopia (relative risk = 7.15, 95% CI 4.01–12.74) (25).

### Cataract

According to a summary meta-analysis that combined cohort, case-control, and cross-sectional studies, myopia was significantly associated with a higher rate of nuclear and posterior subcapsular (PSC) cataract (16). However, the association of myopia with nuclear cataracts or PSC cataracts was not significant in cohort studies. For cortical cataracts, there was no significant association in any observational, case-control/cross-sectional, or cohort study (16). Similar results were provided by another meta-analysis through a random-effects model (relative risk = 2.51, 95% CI 1.53–4.13 for nuclear cataract; relative risk = 2.09, 95% CI 1.60–2.74 for PSC cataract; relative risk = 1.15, 95% CI 0.94–1.40 for cortical cataract).

From mild myopia to high myopia, there was an increasing risk of nuclear or PSC cataract (13). However, no significant association was found between cortical cataracts and mild, moderate, or high myopia (13).

### Epiretinal Membrane

People with myopia or hyperopia had a higher but non-significant risk of having an epiretinal membrane (17).

### AMD

**Figure 5:**
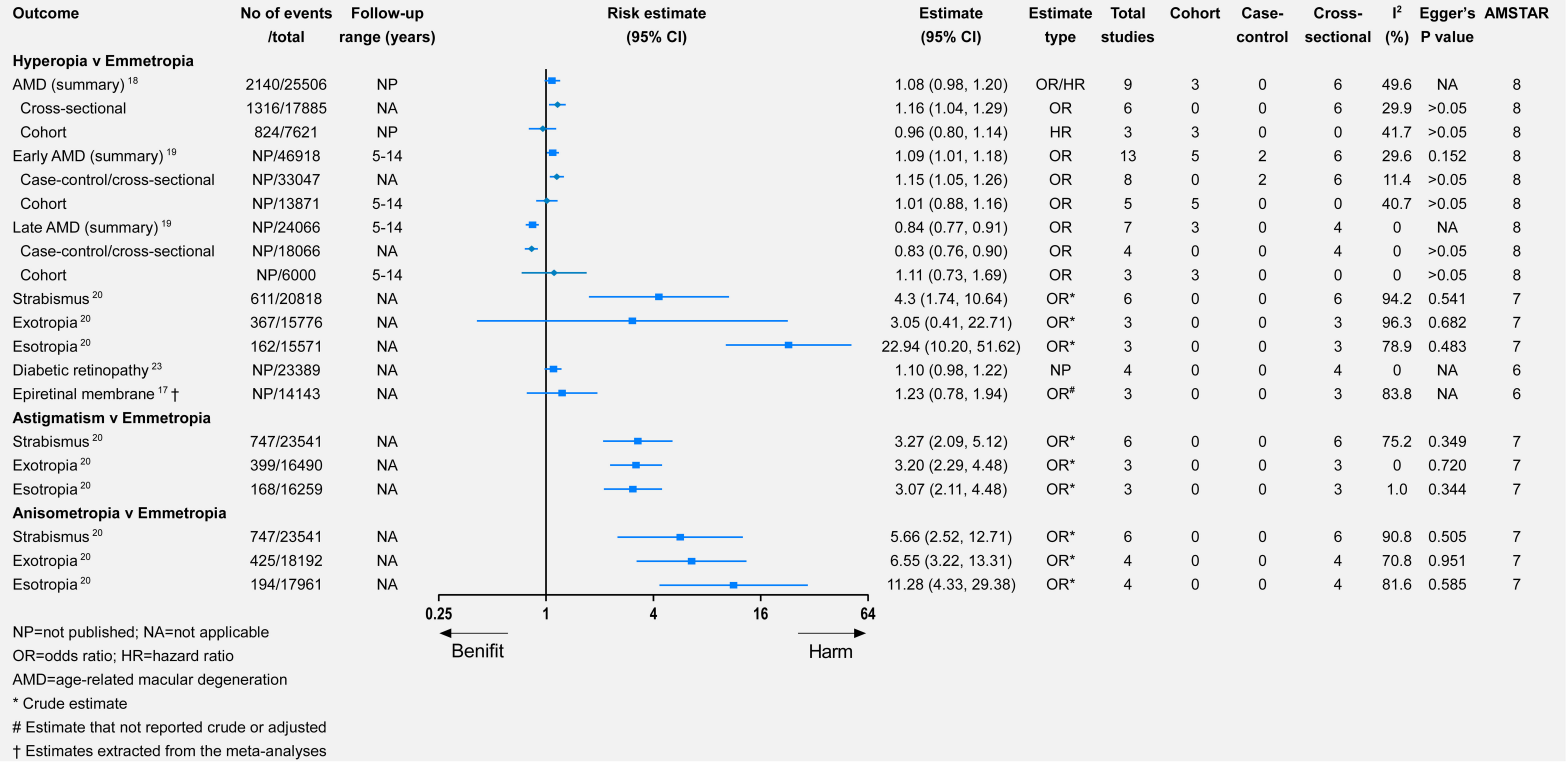
Associations of hyperopia, astigmatism, and anisometropia with multiple eye health outcomes.

The diagnosis and classification of AMD were based on the Wisconsin Grading System or the International AMD Classification (18, 19). Myopia was significantly associated with a lower risk of AMD (relative risk = 0.79, 95% CI 0.69–0.92). By contrast, the hyperopia population had a higher but non-significant risk of AMD (relative risk = 1.08, 95% CI 0.98–1.20). Regarding the associations between refractive error and early AMD, they were similar to those between refractive error and AMD (Figures 2, 5). For late AMD, myopia was associated with a lower but non-significant risk (relative risk = 0.73, 95% CI 0.42–1.26), while hyperopia was significantly associated with a lower risk of late AMD (relative risk = 0.84, 95% CI 0.77–0.91). However, when we focused on cohort studies, there were no significant associations between refractive error and incident AMD, incident early AMD, or incident late AMD (Figures 2, 5).

**Figure 6:**
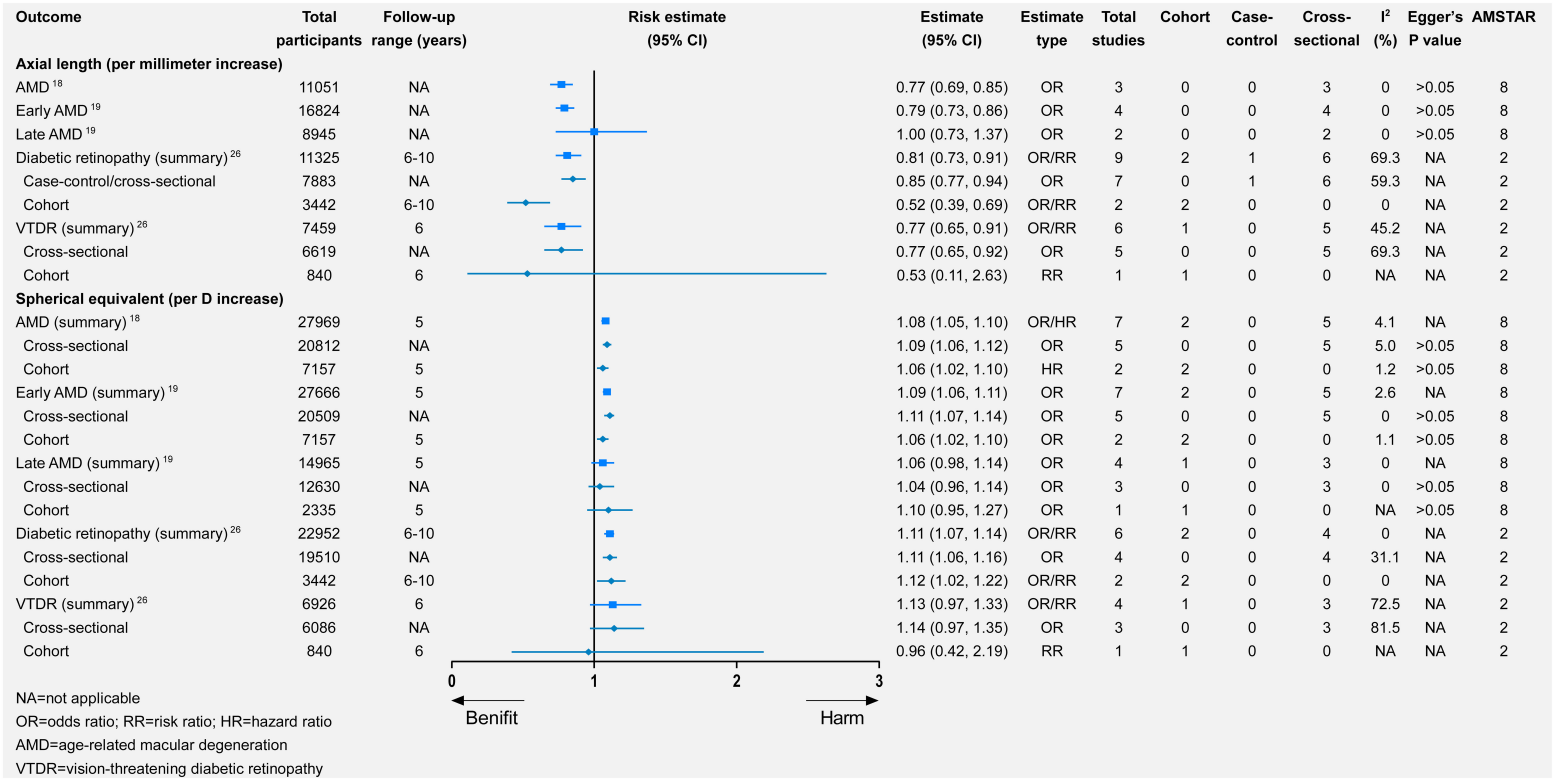
Associations of changes in axial length and spherical equivalent with multiple eye health outcomes.

Per-diopter increase in SE toward hyperopia was significantly associated with a higher risk of AMD, which was consistent in both prevalent and incident AMD (Figure 6). Likewise, per-diopter increase in SE toward hyperopia was associated with early AMD (with significance) or late AMD (without significance; Figure 6). The data for the association between the per-millimeter increase in axial length and AMD were available in cross-sectional studies. After the meta-analysis, a per-millimeter increase in axial length was associated with a lower risk of both AMD and early AMD but was unrelated to late AMD (Figure 6).

### DR and VTDR

There were consistently associations between myopia and DR, such as any (relative risk = 0.83, 95% CI 0.66–1.04), mild (relative risk = 0.73, 95% CI 0.58–0.92), moderate (relative risk = 0.69, 95% CI 0.50–0.96), and high (relative risk = 0.41, 95% CI 0.16–1.04) myopia. The protective effect increased from mild-to-high myopia, although there was no significance for the association between any/high myopia and DR. The associations between different types of myopia and VTDR were not significant or consistent (Figures 2, 3).

Per-diopter increase in SE toward hyperopia was significantly associated with a higher risk of DR (relative risk = 1.11, 95% CI 1.07–1.14). There was also a beneficial but non-significant association between per-diopter increase and VTDR. Furthermore, a per-millimeter increase in axial length was associated with a lower risk of DR and VTDR (Figure 6).

### Strabismus

Only one study meta-analyzed the association between refractive error and strabismus (20). All four types of refractive error were significantly associated with strabismus, of which anisometropia had the highest risk of strabismus (relative risk = 5.66, 95% CI 2.52–12.71), while myopia had the lowest risk (relative risk = 3.22, 95% CI 1.87–5.56). Myopia was a significant risk factor for exotropia, whereas hyperopia was significantly associated with a higher risk of esotropia. Astigmatism and anisometropia were significantly associated with a higher risk of exotropia or esotropia.

### Heterogeneity

We reanalyzed 58 meta-analyses using a random-effect model. Thirty-nine of the reanalyzed meta-analyses had an *I*^2^ <50%. For the 26 meta-analyses that we were unable to reanalyze, 18 had an *I*^2^ <50%. Additionally, 18 meta-analyses used the random-effects model.

### Publication Bias

We performed Egger's test in only seven meta-analyses in our reanalysis. For the remaining 77 meta-analyses, 92% contained an insufficient number of studies, while 8% did not provide the primary data. In those, we reanalyzed that about 29% had significant publication bias, such as any myopia vs. no myopia/emmetropia for OAG (*P* = 0.089) (21) and early AMD (*P* = 0.02) (19). For meta-analyses that we were unable to reanalyze, none exhibited significant publication bias, or they did not conduct publication bias analysis. However, four studies reported that all *P*-values of Egger's test were larger than 0.05 instead of providing the exact *P*-values (15, 16, 18, 19). Three studies reported *P*-values of specific comparisons for specific outcomes (21, 23, 24). In these three studies, two studies considered *P* < 0.05 as significant, and the remaining did not set the criteria.

### AMSATAR and GRADE Classification

**Figure 7:**
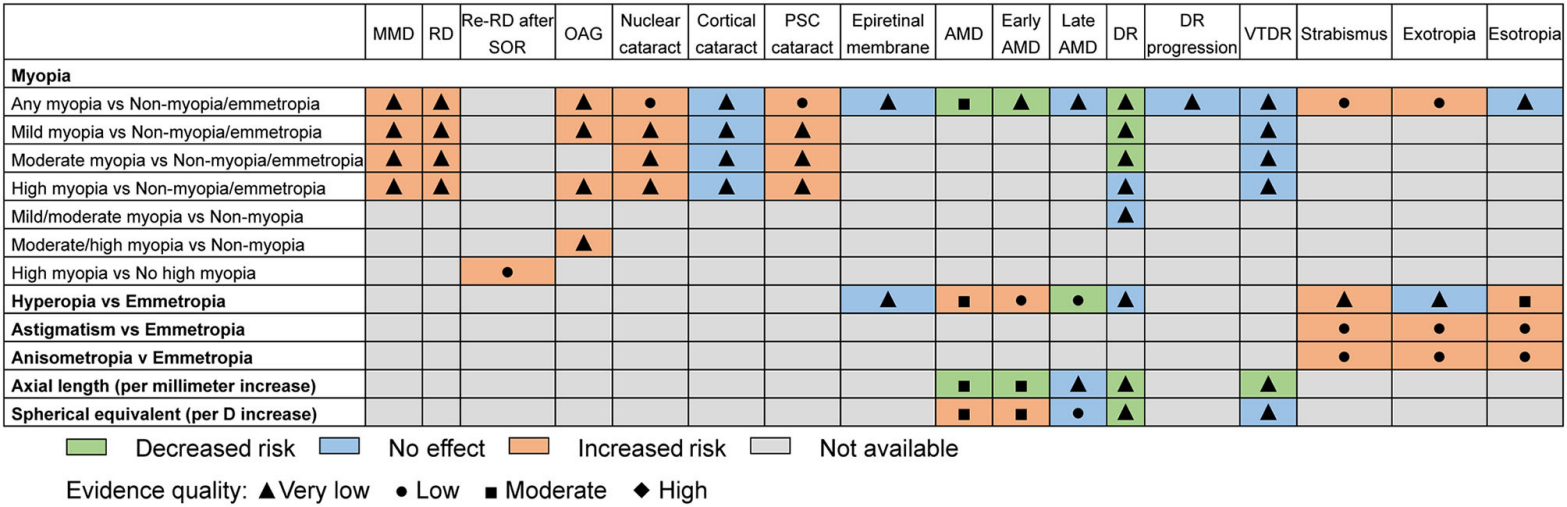
Evidence map of refractive error with regard to eye health outcomes. MMD, myopic macular degeneration; RD, retinal detachment; Re-RD, retinal re-detachment; SOR, silicone oil removal; OAG, open angle glaucoma; PSC, posterior subcapsular; AMD, age-related macular degeneration; DR, diabetic retinopathy; VTDR, vision-threatening diabetic retinopathy.

The median AMSTAR score was 7 across all included studies (range 2–8). The most frequent item downgrading the AMSTAR score was “Was a list of studies (included and excluded) provided?” (14 out of 15), followed by “Was an a priori design provided?” and “Was the status of the publication (i.e., gray literature) used as an inclusion criterion?” (13 out of 15). Supplementary Table 2 provides the breakdown of AMSTAR scores for each study. Figure 7 shows an evidence map based on the quality of evidence. For GRADE classification of the outcomes included in the umbrella review, about 67.2% (*n* = 45) were rated as “very low,” 22.4% (*n* = 15) as “low,” and 10.4% (*n* = 7) as “moderate.” Supplementary Material 4 provides the breakdown of GRADE scores for each included outcome.

## Discussion

Because refractive errors, especially myopia, are a global problem, studies have focused on their influence on eye health outcomes and vision impairment (2, 4, 13). While previous systematic reviews and meta-analyses have separately explored the associations between refractive errors and various eye health outcomes (13–15, 17, 18, 20, 22), an overall summary of these associations is still unavailable. This umbrella review provides a comprehensive overview of reported associations between refractive errors and multiple eye health outcomes by extracting and recalculating the data from systematic reviews and meta-analyses.

Myopia was strongly associated with a higher risk of MMD, and this association was increased with myopia progression. Studies have reported that the prevalence of MMD ranges from 1.2 to 21.7% in mild myopia, 7.1 to 49.1% in moderate myopia, and 61.3 to 76.4% in high myopia (29–32). These prevalence values are consistent with our results. Axial elongation in myopic eyes, stretching force on the wall of the eyeball, and subsequent staphyloma contribute to irreversible retinal photoreceptor damage (33). These changes in myopia may induce myopic maculopathies, such as chorioretinal atrophy, choroidal neovascularization, myopic macular hole, foveoschisis, dome-shaped macula, and macular Bruch membrane holes (33).

Our meta-analysis demonstrated a higher risk of RD in the myopia group, and the risk was increased for those with more severe myopia. Furthermore, patients with high myopia also had a 2.53-fold risk of re-RD. Han et al. reported a significantly negative association between a per-diopter increase in SE and RD (OR = 0.72, 95% CI 0.69–0.76) (34). For each 6D decrease in SE (from emmetropia to high myopia), the risk of RD increases 7.2-fold (95% CI 5.19–9.27) (34), which was lower than that in our meta-analysis. Myopia, especially high myopia, is associated with vitreous liquefaction, PVD, and lattice degeneration (35–37). As myopia alters collagen and hyaluronan association causing gel liquefaction and fibrous degeneration (38), abnormal vitreous liquefaction, and PVD occur earlier than normal. Compared with non-high myopic eyes, myopic eyes are prone to have asymmetrical PVDs, multiple PVDs, and multilayered PVDs (37, 39). Moreover, a thickened vitreous accompanied by para-vascular lamellar holes was also observed to adhere to the retinal vessel at multiple points (39). These vitreous and retinal changes are risk factors for rhegmatogenous RD. Moreover, axial elongation leads to retinal thinning to maintain its coverage of the inner surface of the choroid-scleral shell (40), which contributes to RD. Additionally, the myopic maculopathies mentioned above, such as posterior scleral staphyloma, macular hole, and macular retinoschisis, are associated with foveal RD (41, 42). All these myopic changes above contribute to RD.

The prevalence of OAG was higher in the myopia group than in the emmetropia group. The risk of OAG was higher in the more severe myopia group. Hollands et al. reported that from a myopia population with diopter ≤ −0.5 D to ≤ −8 D, the prevalence of OAG increased from 3.5 to 10% non-linearly (43). The mechanism of OAG caused by myopia is still unclear, but it is probably associated with the increased stretching force in longer axial length eyes (44). The lamina cribrosa and peripapillary scleral flange are stretched and thinned in myopic eyes caused by axial elongation (45). Moreover, scleral rigidity decreases in longer eyes, and the subsequent loss of biomechanical support at and around the lamina cribrosa exposes retinal ganglion cell axons to mechanical strain as they traverse the porous lamina cribrosa (46–48). This mechanical force may have a pathologic role in glaucoma. In addition, the misdiagnosis of myopic retinopathy and glaucoma may increase the relative risk of OAG.

Myopia was associated with prevalent nuclear and PSC cataracts but not with cortical cataracts. With myopia progression, the risk of nuclear and PSC cataracts increased. Nuclear cataract results from an aging process that is associated with lens fiber accumulation and abnormal lens protein aggregation (49, 50), which can be induced by oxidative stress (51). The oxidative stress level increases and antioxidant capacity decreases in the myopic eyes (52), which may partly accelerate the nuclear cataract. However, the myopic shift (53, 54) in nuclear cataracts probably exaggerates the association between myopia and prevalent nuclear cataract, which is supported by the result that no significant association was found between myopia and incident nuclear cataract. A previous study reported that a myopic refractive shift occurred only in persons with nuclear opacity levels of four or higher (55). Consequently, the influence of myopic shift is especially obvious for advanced nuclear cataracts. People without nuclear cataracts had an annual mean hyperopic shift of 0.05 diopters (55), which could also exaggerate the effect of myopia on nuclear cataracts. Besides, the possible bias in considering some high myopes as patients with advanced nuclear cataracts in the elders should also be noted and carefully explained. Additionally, a 4–5-year follow-up may be insufficient for cataract formation. Vitreous environment change, as occurs with vitrectomy, may play an important role in PSC cataract (56). A high level of malondialdehyde in the lens and the vitreous of myopic eyes (57) may play a role in myopia-associated PSC cataracts. This hypothesis is supported by an animal study where the injection of peroxidative substances into the vitreous resulted in the formation of PSC cataract (58).

The risk of prevalent AMD was significantly lower in the myopia group and higher in the hyperopia group. This association primarily existed in early AMD but not in late AMD. The association between incident AMD and refractive error was not significant. Dose-response analyses also indicated that myopia progression (per-millimeter increase in axial length and per D decrease in SE) was associated with a lower risk of early AMD but not for late AMD. However, our re-analysis showed that hyperopia was associated with a lower risk of late AMD unlike noted in a previous meta-analysis (19). Therefore, the association between refractive error and late AMD requires further investigation. The precise mechanism underlying the association between refractive error and AMD is unclear. Previous studies reported that scleral thickness and rigidity were increased in shorter eyes (48, 59, 60), leading to increased choroidal vascular resistance. The subsequently impeded choroidal blood inflow and outflow reduce the transfer of oxygen and nutrients and ultimately impair the retinal pigment epithelium. The hypoxic conditions in hyperopic eyes may cause increased vascular endothelial growth factor (VEGF) production, consequently promoting neovascularization (61, 62). However, most previous studies reported that myopic eyes, especially highly myopic eyes, had reduced ocular blood flow, such as decreased peripapillary vessel density, lower superficial and deep parafoveal vessel density, larger avascular zone, and narrowed choroidal vessel diameter (63–67). Accompany with the reduced ocular blood supply, the retinal arteriolar oxygen saturation was also reduced (63, 68). Therefore, the exact mechanism for the association of refractive error and AMD needs further exploration. Furthermore, the larger ocular volume in myopic eyes probably dilutes intraocular VEGF and results in decreased angiogenesis. The findings in our umbrella review are in accordance with regard to these mechanisms.

Myopia was significantly associated with a lower risk of prevalent DR but was not significantly associated with incident DR and VTDR. Dose-response analysis showed that a per-millimeter increase in axial length was protective for prevalent VTDR but not for incident VTDR. However, the study number and sample size of incident VTDR were small; thus, caution is necessary while interpreting the results. A contralateral eye study reported that DR was significantly less frequent in the high myopic eye (relative risk = 0.28, 95% CI 0.21–0.37) (69). Similar significant trends were reported for the incidence of non-proliferative (27.6 vs. 69%, *P* < 0.001) and proliferative DR (0 vs. 31%, *P* < 0.001) (69). These findings are consistent with ours. Several theories have attempted to explain the association. One is the decreased retinal blood flow in eyes with a longer axial length (70). Retinal blood flow increases in DR progression (71–73), which may result in increased retinal capillary pressure and subsequently, capillary wall dilatation, leakage, and rupture. However, Cuypers et al. reported that compared with non-proliferative and pre-proliferative DR, the retinal blood flow was reduced in proliferative DR (74). Therefore, retinal blood flow may not be a suitable parameter for interpreting the associations between myopia and DR. Another theory is that the retinal hypoxia in myopic eyes is reduced. Retinal hypoxia is regarded to play a vital role in DR progression (75–77). As mentioned above, hypoxia induces VEGF production and consequently promotes angiogenesis (61, 62), which causes the development of DR. Decreased scleral thickness and rigidity in myopic eyes (60) reduce retinal vascular resistance and retinal hypoxia, leading to the prevention of DR progression. In addition, the dilution effect of the increased volume and the lowered metabolic demand of elongated eyes are also potential protective factors for DR (78).

Our findings showed that myopia was a risk factor for exotropia, while hyperopia increased the risk of esotropia, and astigmatism and anisometropia were associated with a higher risk of concomitant strabismus. A possible mechanism underlying these associations is convergence stimulation. For myopia, the less accommodative effort is required for clear images because of a larger mean lag of accommodation (79). These fewer accommodations results in the less accommodative convergence stimulation (80), which probably breaks down the fusional control and leads to exotropia. In contrast, the more accommodative effort is needed for hyperopia eyes at a distance of 100 or 33 cm (6). The subsequent persistent convergence stimulation predisposes the eyes to esotropia. However, the precise mechanism is undetermined.

However, we should notice that the possible role of misclassification in these analyses. For OAG, it is difficult to clearly distinguish the fundus changes of myopia. Posterior scleral staphyloma and increased stretching force in eyes with longer axial length may increase the C/D ratio and reduce the retinal nerve fiber layer thickness. Besides fundus changes, abnormal OCT RNFL and visual field defects due to peripapillary atrophy and choroidal thinning can also occur in myopes (Supplementary Material 7), making the diagnosis difficult. These would exaggerate the risk of OAG in myopia. Additionally, it is possible that macular degeneration may be influenced by knowledge of myopia. MMD is defined as macular degeneration that occurs in people with severe myopia. Therefore, any macular degeneration that occurs in people without severe myopia or even myopia is unlikely to be called MMD. This macular degeneration may be attributed to other causes such as AMD, which may explain the apparent protective associations between myopia and AMD.

Our study is the first umbrella review to systematically summarize the association between refractive errors and eye health. We extracted the data in our study from meta-analyses alone. Our study also provides dose-response analyses, which could help the investigator better assess the relationship between refractive error and different eye health outcomes.

There are some limitations to our study. First, except for one RCT (the intervention was not for myopia) (81), all the primary studies in the meta-analyses were observational studies, which reduces the evidence level. However, to our knowledge, it is generally infeasible to conduct an RCT for refractive error. Second, some outcomes lacked dose-response analyses, making it difficult to determine the effect of different refractive conditions. Third, because the focus of our study was to provide broad-based evidence for refractive error-related eye health from existing meta-analyses, we did not conduct subgroup analyses with regard to variables such as sex, age group, and race. Fourth, we did not update the data by combining the primary studies from previously published meta-analyses with studies published after these meta-analyses were conducted.

This umbrella review comprehensively analyzed the association between refractive errors and various eye health outcomes. Myopia had harmful associations with most eye health outcomes, such as MMD, RD, OAG, nuclear and PSC cataracts, and exotropia. These associations were more obvious in eyes with high myopia. A decreased risk of DR or early AMD was associated with myopia. The association between myopia and VTDR or late AMD remains uncertain but warrants further investigations. Hyperopia was a risk factor for early AMD and esotropia but was associated with a lower risk of late AMD. Astigmatism and anisometropia showed harmful associations with both exotropia and esotropia. Our results provide higher-level evidence for investigators to understand refractive errors. With the bidirectional effects of refractive errors, especially myopia, the optimal refractive error status should be reconsidered and investigated. Finally, the findings of this umbrella review can be of great clinical interest. The risk of myopia is usually remembered by most clinicians, however, besides the risks of angle closure in hyperopes, the increased risk of AMD in hyperopes should be noted and kept in mind in a routine clinical setting.

## Data Availability Statement

The raw data supporting the conclusions of this article will be made available by the authors, without undue reservation.

## Author Contributions

Y-hW: conceptualization, methodology, software, formal analysis, resources, data curation, writing-original draft, and visualization. CH: conceptualization, validation, resources, investigation, writing—review, and editing. Y-lT: validation, formal analysis, investigation, writing—review, and editing. JZ: investigation, writing—review, and editing. X-mL: conceptualization, validation, writing—review and editing, supervision, project administration, and funding acquisition. All authors contributed to the article and approved the submitted version.

## Funding

This study was supported by the National Science Foundation of Beijing Municipality [grant number: 7202229].

## Conflict of Interest

The authors declare that the research was conducted in the absence of any commercial or financial relationships that could be construed as a potential conflict of interest.

## Publisher's Note

All claims expressed in this article are solely those of the authors and do not necessarily represent those of their affiliated organizations, or those of the publisher, the editors and the reviewers. Any product that may be evaluated in this article, or claim that may be made by its manufacturer, is not guaranteed or endorsed by the publisher.
