# Supplementary material for: Refractive Error and Eye Health: An Umbrella Review of Meta-Analyses
Source: Front Med (Lausanne). 2021 Nov 4;8:759767. doi: 10.3389/fmed.2021.759767 (PMC8599990; doi:10.3389/fmed.2021.759767)
Supplement: Supplementary file 1 [file Data_Sheet_1.zip › 759767_Li_Supplementary1.docx]

**Supplementary Table 1. Characteristics of the included meta-analyses**

| **Study** | **Search time** | **Refractive error and outcome definition** | **Outcomes** | **Type of data** | **Database (n)** | **Guideline** | **Quality** | **Publication bias** | **Total study (n)** |
| --- | --- | --- | --- | --- | --- | --- | --- | --- | --- |
| Haarman 2020 | 2019/6/1 | **No myopia:** SE > -0.5 D  **Any myopia:** SE ≤ -0.5 D  **Mild myopia:** SE < -0.5 to > -3 D  **Moderate myopia:** SE ≤-3 to > -6 D  **High myopia:** SE ≤ -6.00 D  **MMD:** a [excluding tessellation (n=3)]; b [(≥M3; excluding tessellation) (n=1)]; c [excluding tessellation (n=3)]; d [excluding tessellation (n=1)]  **RD:** not mentioned  **Cataract:** not mentioned  **OAG:** IOP (n=3); GVFL (n=13); optic disc abnormalities (n=8); CD-ratio increase or asymmetry (n=7); reduced NRRW (n=2); family history of glaucoma (n=1); history of treated glaucoma (n=1) | MMD | 8 cross-sectional | 1 | PRISMA | Sanderson et al^1^ | ND | 46 |
|  |  |  | RD | 5 case-control |  |  |  |  |  |
|  |  |  | PSC cataract | 3 cohort  1 case-control  7 cross-sectional |  |  |  |  |  |
|  |  |  | Nuclear cataract | 3 cohort  1 case-control  7 cross-sectional |  |  |  |  |  |
|  |  |  | Cortical cataract | 3 cohort  1 case-control  7 cross-sectional |  |  |  |  |  |
|  |  |  | OAG | 14 cross-sectional |  |  |  |  |  |
| Fu 2016 | 2015/4 | **Myopia:** SE≤-0.05 D (n=2); SE≤-0.75 D (n=1); SE≤-1 D (n=1); SE≤-2 D (n=2); not mentioned (n=2); not applicable (n=3)  **DR**=NPDR+PDR  NPDR: microaneurysms, hard exudates, and retinal hemorrhages  PDR: the presence of newly formed blood vessels, fibrous proliferations, and vitreous hemorrhage | DR | 2 cohort  9 cross-sectional | 6 | MOOSE | NOS | Begg’s and Egger’s tests | 11 |
| Wang 2016 | 2015/3 | **Myopia:** SE < -0.5 D  **Any DR:** the presence of NPDR, PDR, DME, or any combination  **VTDR:** the presence of severe NPDR, PDR, and/or DME | DR | 1 cohort  6 cross-sectional | 2 | - | Sanderson et al^1^ | Begg’s and Egger’s tests | 9 |
|  |  |  | VTDR | 5 cross-sectional |  |  |  |  |  |
| Pan 2013a | 2013/3 | **Myopia:** SE<-0.5 D (n=5); SE≤-0.5 D (n=1); SE<-1 D (n=1); SE≤-1 D (n=4); SE≤-1.5 D (n=1)  **Cataract grading system**:  Wisconsin Grading System (n=5); Lens Opacities Classification System II (n=2); Lens Opacities Classification System III (n=3); Wilmer Grading System (n=2) | Nuclear cataract | 4 cohort  1 case-control  7 cross-sectional | 2 | MOOSE | Sanderson et al^1^ | Begg’s and Egger’s tests | 12 |
|  |  |  | Cortical cataract | 3 cohort  1 case-control  7 cross-sectional |  |  |  |  |  |
|  |  |  | PSC cataract | 3 cohort  1 case-control  7 cross-sectional |  |  |  |  |  |
| Xiao 2017 | 2016/7 | **Refractive error:** no definition  **Epiretinal membrane diagnosis:** fundus photography (n=2); fundus photography & OCT (n=2) | Epiretinal membrane | 4 cross-sectional | 3 | PRISMA | de Weerd et al^2^  Rogers et al^3^ | ND | 4 |
| Pan 2013b | 2012/7 | **Myopia:** SE<-0.5 D (n=4); SE≤-0.5 D (n=2); SE<-1 D (n=1); SE≤-1 D (n=2)  **Hyperopia:** SE >0.5 D (n=4); SE≥0.5 D (n=2); SE >1 D (n=1); SE≥1 D (n=2)  **AMD diagnosis:** fundus photographs based on standardized protocol (Wisconsin Grading System, or International AMD Classification) | AMD | 3 cohort  6 cross-sectional | 2 | - | Sanderson et al^1^ | Begg’s and Egger’s tests | 9 |
| Li 2014 | 2013/7/27 | **Myopia cutoff** (n=13): -0.5 D (n=6); -0.75 D (n=1); -1 D (n=3); Not mentioned (n=3)  **Hyperopia cutoff** (n=15): 0.5 D (n=6); 0.75 D (n=1); 1 D (n=5); Not mentioned (n=3)  **AMD diagnosis:**  Wisconsin Grading System (n=10)  International AMD Classification (n=5) | Early AMD | 5 cohort  2 case-control  6 cross-sectional | 3 | - | NOS | Begg’s and Egger’s tests | 15 |
|  |  |  | Late AMD | 3 cohort  4 cross-sectional |  |  |  |  |  |
| Tang 2016 | 2016/4/1 | **Myopia:** SE≤-0.5 D (n=4); SE≤-1 D (n=2); not applicable (n=1)  **Hyperopia:** SE≥0.5 D (n=2); SE≥2 D (n=4); not applicable (n=1)  **Astigmatism:** cylinder≥ 0.5 D (n=1); cylinder≥ 1 D (n=5); not applicable (n=1)  **Anisometropia:** SE difference≥1 D (n=7)  **Strabismus diagnosis:** cover and uncover test | Strabismus | 6 cross-sectional | 2 | - | Modified Estabrooks’ Quality Assessment and Validity Tool | Egger’s test | 7 |
|  |  |  | Exotropia | 4 cross-sectional |  |  |  |  |  |
|  |  |  | Esotropia | 4 cross-sectional |  |  |  |  |  |
| Marcus 2011 | 2010/10/27 | **Myopia:** SE≤-0.01 D (n=1); SE<-0.5 D (n=5); SE≤-0.5 D (n=2); SE<-1 D (n=1); SE≤-1 D (n=3); SE<-1.5 D (n=1)  **Mild myopia:** SE up to >-3 D  **High myopia:** SE≤-3 D  **Definition of glaucoma:** IOP (n=3); history of treated glaucoma (n=2); GVFL (n=12); optic disc abnormalities (n=6); CD-ratio increase or asymmetry (n=6); reduced NRRW (n=2); family history of glaucoma (n=1) | OAG | 1 cohort  1 case-control  11 cross-sectional | 2 | - | Sanderson et al^1^ | Begg’s and Egger’s tests | 13 |
| He 2018 | 2017/3/30 | **High myopia:** SE cutoff=-6 D (n=5); Axial length>27 mm (n=1)  **Re-RD:** no definition or diagnosis | Re-RD | 1 RCT  5 case-control | 5 | - | NOS  OCEBM | Inverted funnel plot. | 6 |
| Guo 2015 | 2014/11 | **Myopia:** SE<-1 D  **Mild/moderate myopia:** SE<-1 D to >-5 D  **High myopia:** SE≤-5 D  **Outcomes:** no definition or diagnosis | DR | 1 cohort  6 cross-sectional | 3 | - | Sanderson et al^1^ | Begg’s and Egger’s tests | 8 |
|  |  |  | VTDR | 1 cohort  1 case-control  4 cross-sectional |  |  |  |  |  |
| Xiong 2014 | 2013/5 | **Mild myopia:** SE>-3 D  **Moderate myopia:** SE≤-3 D to >-6 D  **High myopia:** SE≤-6 D  **Definition of glaucoma:** IOP (n=2); GVFL (n=10); optic disc abnormalities (n=4); CD-ratio increase or asymmetry (n=8); reduced NRRW (n=1); family history of glaucoma (n=2) | OAG | 11 cross-sectional | 5 | - | ND | Funnel plot and Egger’s test | 11 |
| Xiang 2014 | 2013/7/20 | **Myopia:** SE≤1 D (n=2); SE ≤1.5 D (n=1); not mentioned (n=2)  **High myopia:** SE<-6 D (n=3); not mentioned (n=2)  **Definition of glaucoma:** IOP>21 mmHg, glaucoma associated optic disc abnormalities, GVFL, and open anterior chamber angle | OAG | 3 case control  2 cross-sectional | 2 | - | ND | ND | 5 |
| He 2021 | NP | - | DR | 2 cohort  1 case-control  6 cross-sectional | 4 | - | ND | ND | 9 |
|  |  |  | VTDR | 1 cohort  5 cross-sectional |  |  |  |  |  |
|  |  |  | DR progression | 2 cohort |  |  |  |  |  |
| Wang 2015 | NP | **High myopia:** SE <-6 D  **Choroidal thickness** was measured by OCT [Topcon 3D-2000 (n=1); Heidelberg Spectralis (n=4); Zeiss Cirrus (n=2); Nidek RS-3000 (n=1)] | Choroidal thickness | Cross-sectional or case-control | 6 | - | ND | Begg’s and Egger’s tests | 7 |

ND=not done; NP=not published

MMD=myopic macular degeneration; RD=retinal detachment; PSC=posterior subcapsular; OAG=open-angle glaucoma; DR=diabetic retinopathy; PDR=proliferative diabetic retinopathy; NPDR=non-proliferative diabetic retinopathy; VTDR=vision-threatening diabetic retinopathy; AMD=age-related macular degeneration; DME=diabetic macular edema; SE=spherical equivalent; IOP=intraocular pressure; GVFL=glaucomatous visual field loss; NRRW=neuroretinal rim width; OCT=optical coherence tomography; Re-RD=retinal re-detachment; PRISMA=Preferred Reporting Items for Systematic reviews and Meta-Analyses; MOOSE=Meta-Analysis of Observational Studies in Epidemiology; NOS= Newcastle-Ottawa Quality Assessment Scale; OCEBM=Oxford Centre for Evidence-Based Medicine

**a:** At least 1 of the following features: staphyloma, lacquer cracks, Fuchs’ spot, and chorioretinal atrophy

**b:** M0, normal-appearing posterior pole; M1, tessellation and choroidal pallor pattern; M2, posterior staphyloma; M3, lacquer cracks; M4, choroidal atrophy; M5, geographic atrophy and choroidal neovascularization (CNV)

**c:** 1: Tessellated fundus, 2: Diffuse chorioretinal atrophy, 3: Patchy chorioretinal atrophy, 4: Macular atrophy, and ‘plus’ lesions: lacquer cracks, myopic CNV and/or Fuchs spot. MMD was defined as ≥2.

**d:** At least 1 of the following features: diffuse chorioretinal atrophy at the posterior pole, patchy chorioretinal atrophy, lacquer cracks, or macular atrophy

**Note:** If there were definitions of the refractive errors or outcomes in the systematic review and meta-analysis, it was used; if not, we directly extracted the number of the different definitions of the primary studies that were provided by the systematic reviews and meta-analyses.

1. Sanderson S, Tatt ID, Higgins JP. Tools for assessing quality and susceptibility to bias in observational studies in epidemiology: a systematic review and annotated bibliography. *Int J Epidemiol*. 2007;36:666-676.

2. de Weerd M, Greving JP, de Jong AW, et al. Prevalence of asymptomatic carotid artery stenosis according to age and sex: systematic review and metaregression analysis. *Stroke*. 2009;40:1105-1113.

3. Rogers S, McIntosh RL, Cheung N, et al. The prevalence of retinal vein occlusion: pooled data from population studies from the United States, Europe, Asia, and Australia. *Ophthalmology*. 2010;117:313-319.e311.
