# Supplementary material for: Refractive Error and Eye Health: An Umbrella Review of Meta-Analyses
Source: Front Med (Lausanne). 2021 Nov 4;8:759767. doi: 10.3389/fmed.2021.759767 (PMC8599990; doi:10.3389/fmed.2021.759767)
Supplement: Supplementary file 1 [file Data_Sheet_1.zip › 759767_Li_Supplementary2.docx]

**Supplementary Table 2. AMSTAR items for individual studies included in the umbrella review**

| Study | A priori design provided | Duplicate study selection and data extraction | A comprehensive literature search | Status of publication used as an inclusion criterion | A list of included and excluded studies provided | Characteristics of the included studies provided | Scientific quality assessed and documented | Scientific quality of the included studies used appropriately in formulating conclusions | Methods used to combine the findings of studies appropriate | Publication bias assessed | Potential conflicts of interest included | Total AMSTAR score |
| --- | --- | --- | --- | --- | --- | --- | --- | --- | --- | --- | --- | --- |
| Haarman 2020 | 1 | 1 | 0 | 0 | 0 | 1 | 0 | 0 | 1 | 0 | 0 | 4 |
| Fu 2016 | 0 | 1 | 1 | 0 | 0 | 1 | 1 | 1 | 1 | 1 | 0 | 7 |
| Wang 2016 | 0 | 1 | 1 | 0 | 0 | 1 | 1 | 1 | 1 | 1 | 1 | 8 |
| Pan 2013a | 0 | 1 | 1 | 0 | 0 | 1 | 1 | 1 | 0 | 1 | 1 | 7 |
| Xiao 2017 | 1 | 1 | 1 | 0 | 0 | 1 | 1 | 1 | 1 | 0 | 0 | 7 |
| Pan 2013b | 0 | 1 | 1 | 0 | 0 | 1 | 1 | 1 | 1 | 1 | 1 | 8 |
| Li 2014 | 0 | 1 | 1 | 0 | 1 | 1 | 1 | 1 | 1 | 1 | 0 | 8 |
| Tang 2016 | 0 | 1 | 1 | 0 | 0 | 1 | 1 | 1 | 1 | 1 | 0 | 7 |
| Marcus 2011 | 0 | 1 | 1 | 0 | 0 | 1 | 1 | 1 | 1 | 1 | 1 | 8 |
| He 2018 | 0 | 1 | 0 | 0 | 0 | 1 | 1 | 1 | 1 | 1 | 0 | 6 |
| Guo 2015 | 0 | 1 | 0 | 0 | 0 | 1 | 1 | 1 | 1 | 1 | 0 | 6 |
| Xiong 2014 | 0 | 1 | 0 | 0 | 0 | 1 | 0 | 0 | 1 | 1 | 0 | 4 |
| Xiang 2014 | 0 | 1 | 0 | 1 | 0 | 1 | 0 | 0 | 1 | 0 | 0 | 4 |
| He 2021 | 0 | 0 | 0 | 0 | 0 | 1 | 0 | 0 | 1 | 0 | 0 | 2 |
| Wang 2015 | 0 | 1 | 1 | 1 | 0 | 1 | 0 | 0 | 0 | 1 | 0 | 5 |

1= “yes”; 0= “No”, “Cannot answer”, or “Not applicable”
