# Supplementary material for: Refractive Error and Eye Health: An Umbrella Review of Meta-Analyses
Source: Front Med (Lausanne). 2021 Nov 4;8:759767. doi: 10.3389/fmed.2021.759767 (PMC8599990; doi:10.3389/fmed.2021.759767)

**Supplementary 3. Re-meta-analyses based on the data extracted from systematical reviews and meta-analyses**

eFigures 1.1~1.3 were based on the data extracted from article 1; eFigures 2.1~2.3 were based on article 2; and so on.

Articles:

1. Fu Y, Geng D, Liu H, et al. Myopia and/or longer axial length are protective against diabetic retinopathy: A meta-analysis. Acta Ophthalmologica. 2016;94:346-352.
2. Wang X, Tang L, Gao L, et al. Myopia and diabetic retinopathy: A systematic review and meta-analysis. Diabetes Res Clin Pract. 2016;111:1-9.
3. Pan C-W, Cheng C-y, Saw S-M, et al. Myopia and Age-Related Cataract: A Systematic Review and Meta-analysis. Am J Ophthalmol. 2013;156.
4. Pan C-W, Ikram MK, Cheung CY, et al. Refractive errors and age-related macular degeneration: A systematic review and meta-analysis. Ophthalmology. 2013;120.
5. Li Y, Wang J, Zhong X, et al. Refractive error and risk of early or late age-related macular degeneration: A systematic review and meta-analysis. PLoS ONE. 2014;9.
6. Tang SM, Chan RYT, Lin SB, et al. Refractive errors and concomitant strabismus: A systematic review and meta-analysis. Sci Rep. 2016;6.
7. Marcus MW, Vries MMd, Montolio FGJ, et al. Myopia as a risk factor for open-angle glaucoma: A systematic review and meta-analysis. Ophthalmology. 2011;118.
8. Yueqing H, Shaoxue Z, Yunni Z, et al. Risk factors for retinal redetachment after silicone oil removal: A systematic review and meta-analysis. Ophthalmic Surg Lasers Imaging Retina. 2018;49.
9. Guo Y. Relationship Between Refractive Error and Diabetic Retinopathy: a Systematic Review and Meta-analysis [Master], ChongQing Medical University; 2015.
10. Xiong Q, Zeng XT, Cai XJ, et al. Association between myopia and open-angle glaucoma: A meta-analysis. Chinese Journal of Evidence-Based Medicine. 2014;14:618-624.
11. Xiang Y, Xia L, Zhang Y, et al. Meta-analysis of association between myopia and primary open angle glaucoma. J Clin Ophthalmol. 2014;22:259-262.
12. He M, Chen H, Wang W. Refractive Errors, Ocular Biometry and Diabetic Retinopathy: A Comprehensive Review. Curr Eye Res. 2021;46:151-158.

eFigure 1.1. Any myopia vs no myopia on diabetic retinopathy.


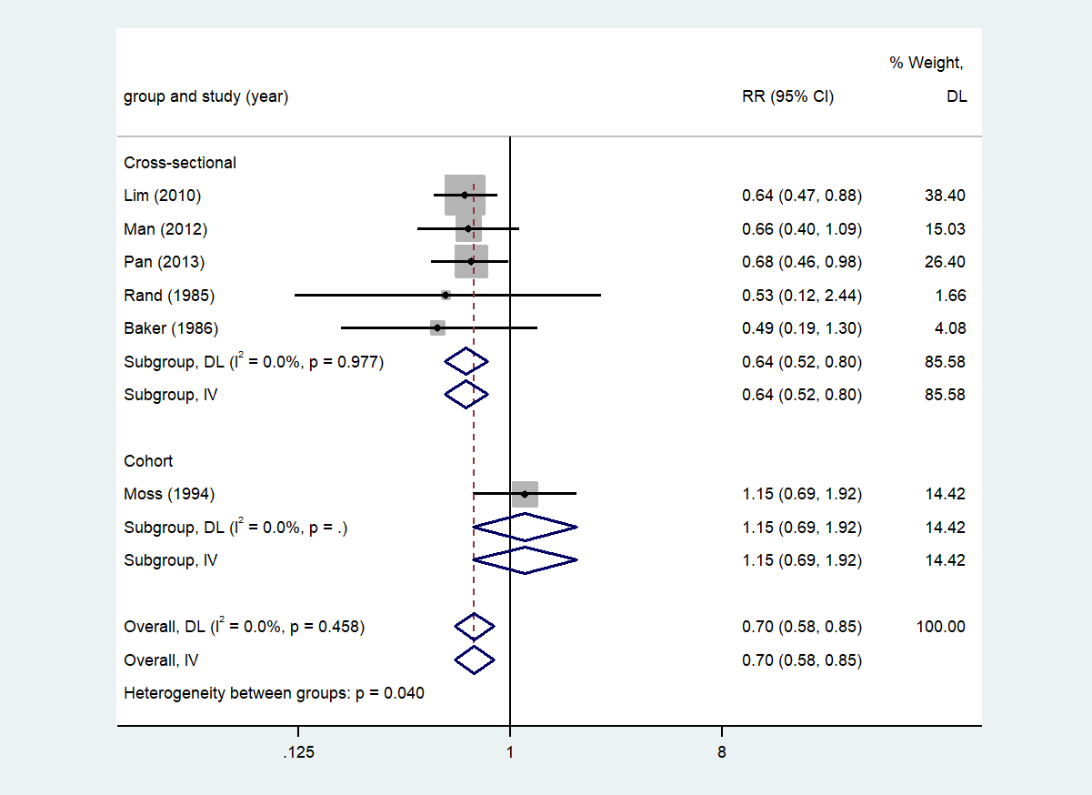


eFigure 1.2. Axial length (per millimeter increase) on diabetic retinopathy.


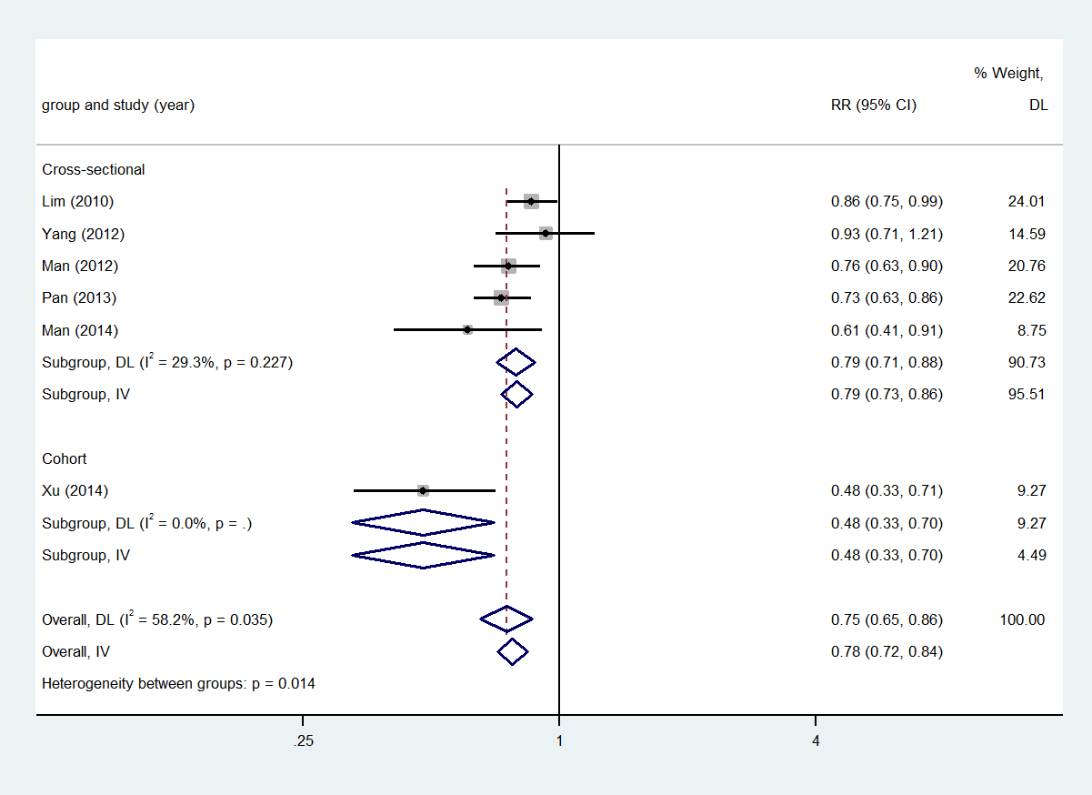


eFigure 1.3. Spherical equivalent (per diopter decrease) on diabetic retinopathy.


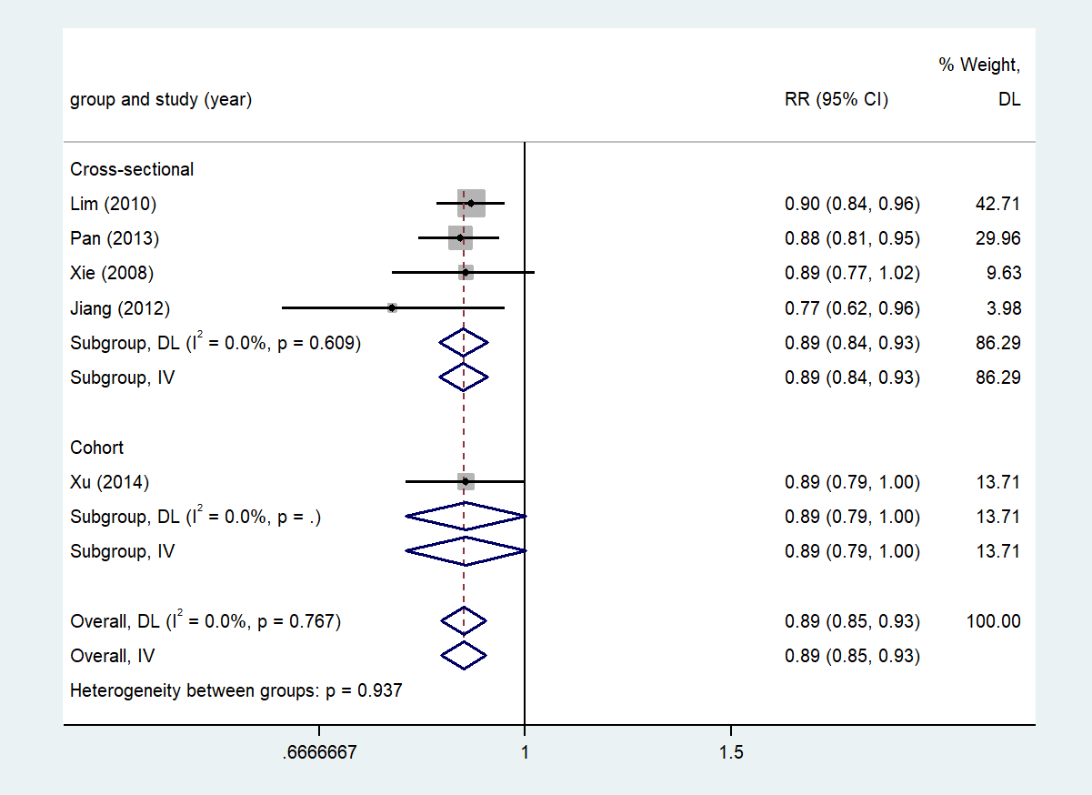


eFigure 2.1. Any myopia vs emmetropia on diabetic retinopathy.


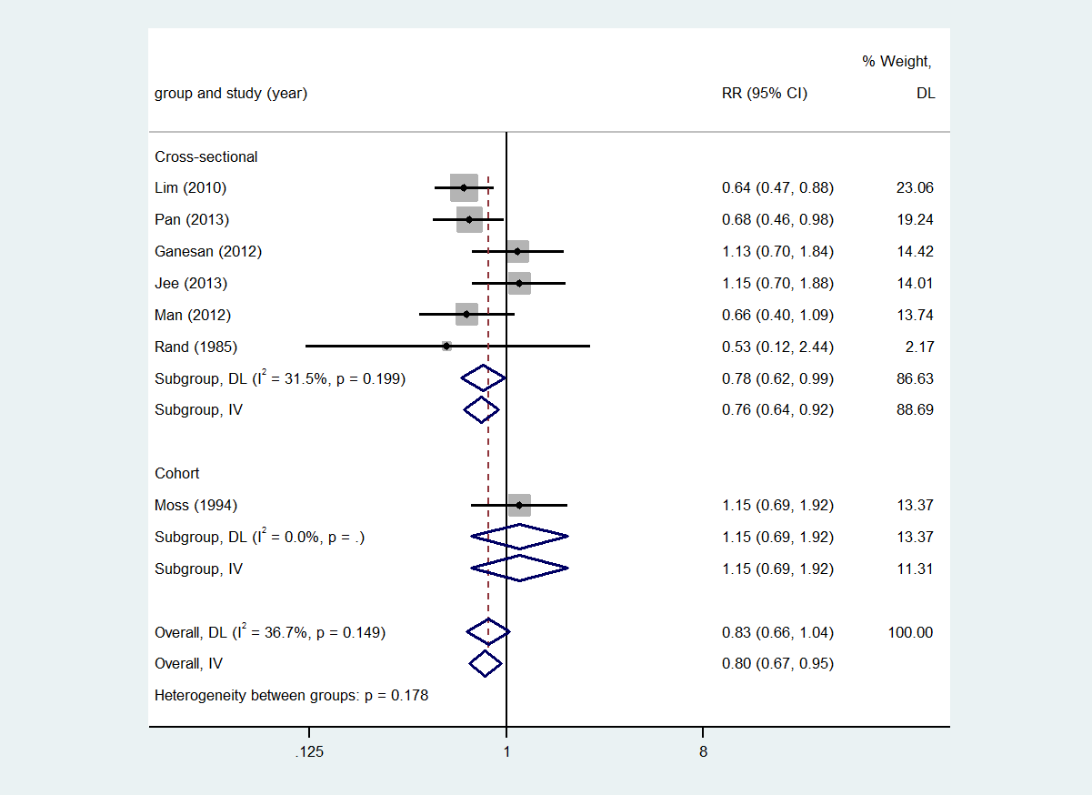


eFigure 2.2. Axial length (per millimeter increase) on diabetic retinopathy.


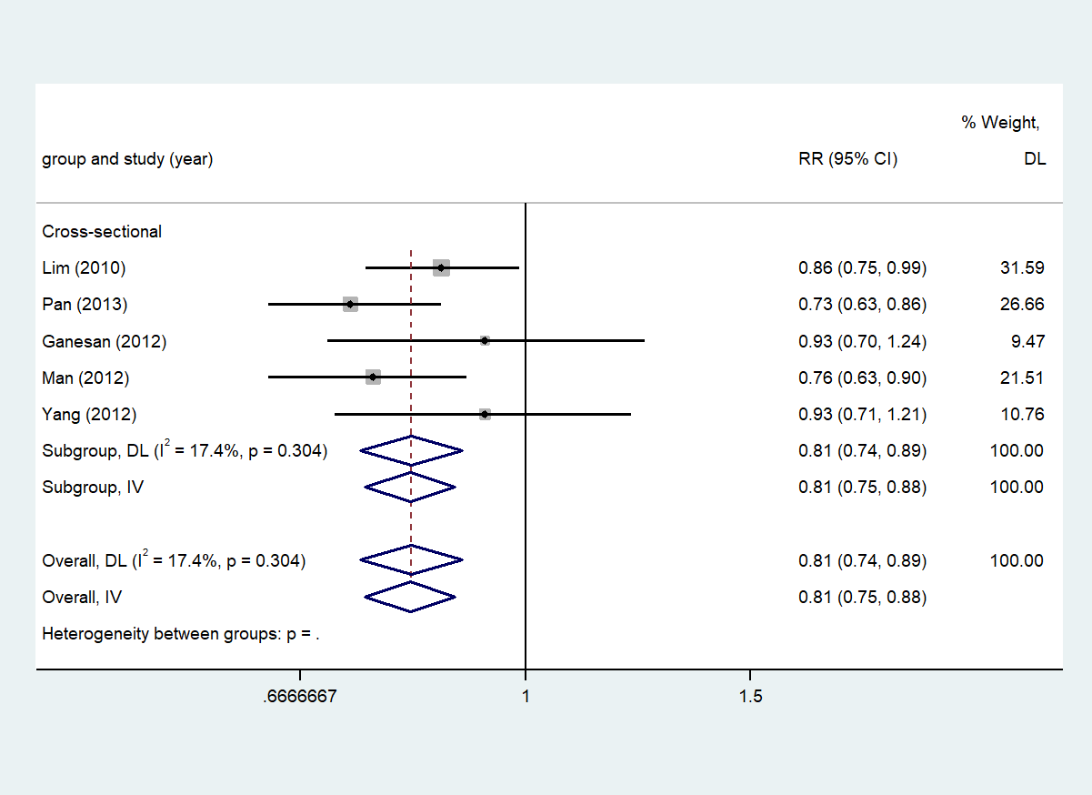


eFigure 2.3. Axial length (per millimeter increase) on vision-threatening diabetic retinopathy.


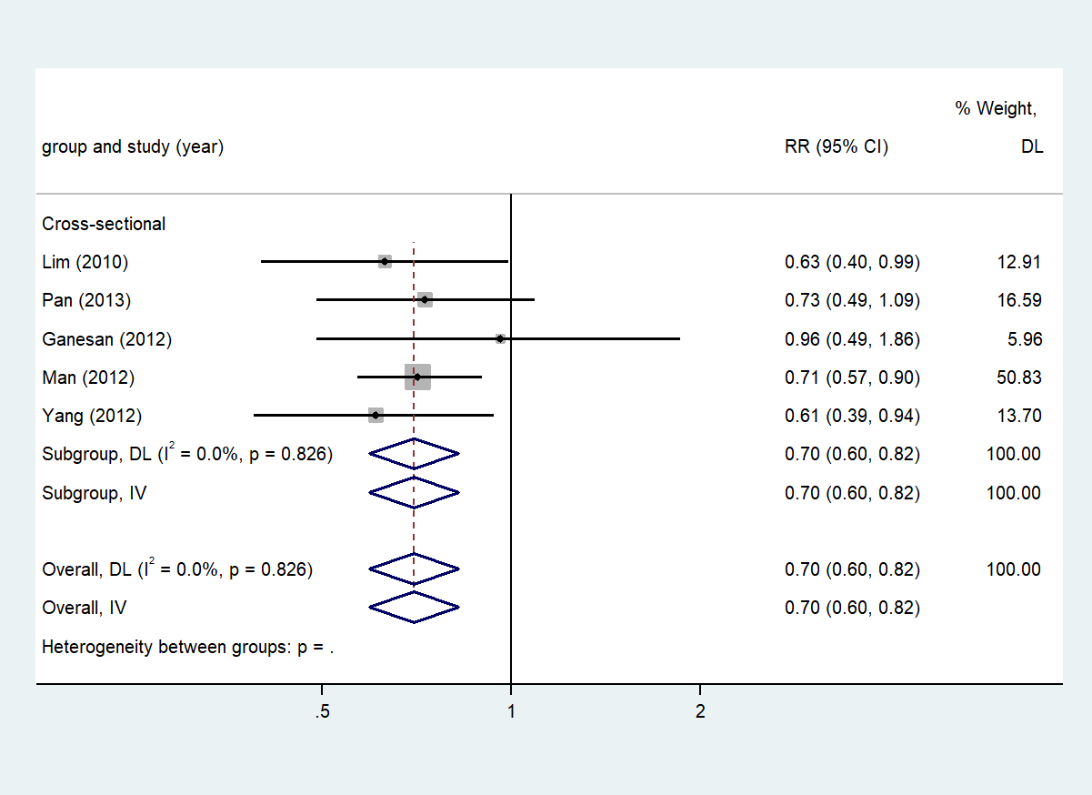


eFigure 3.1. Any myopia vs no myopia on nuclear cataract.


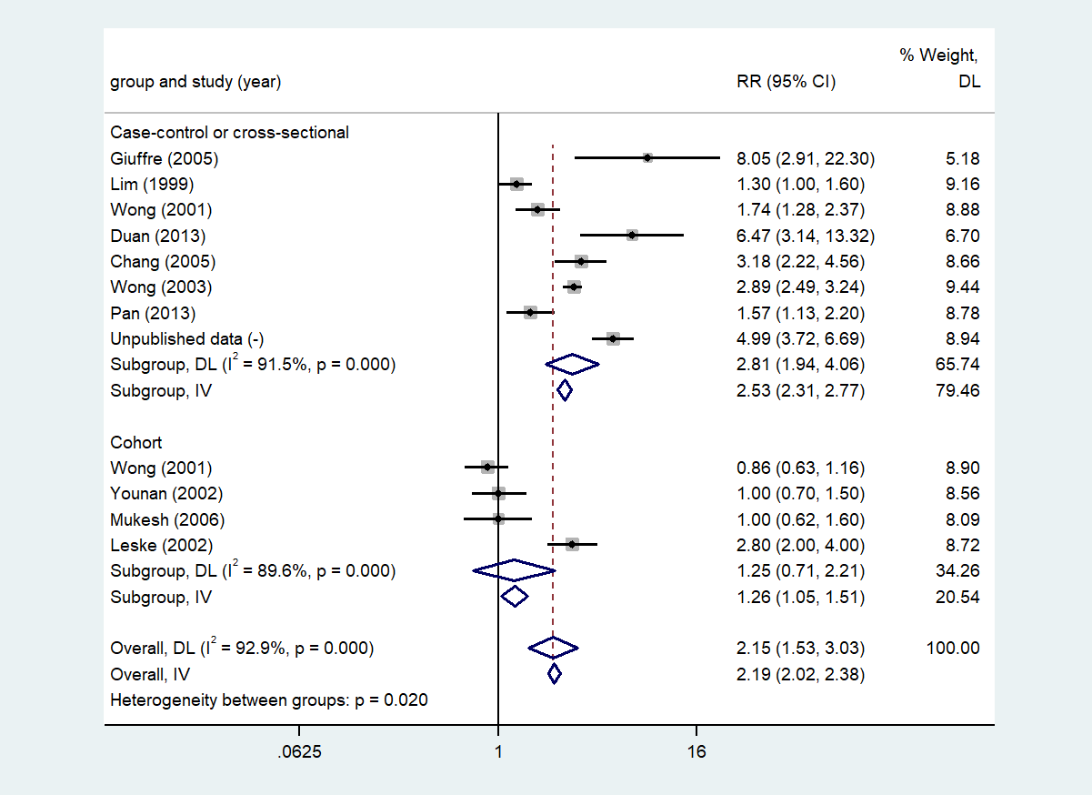


eFigure 3.2. Any myopia vs no myopia on cortical cataract.


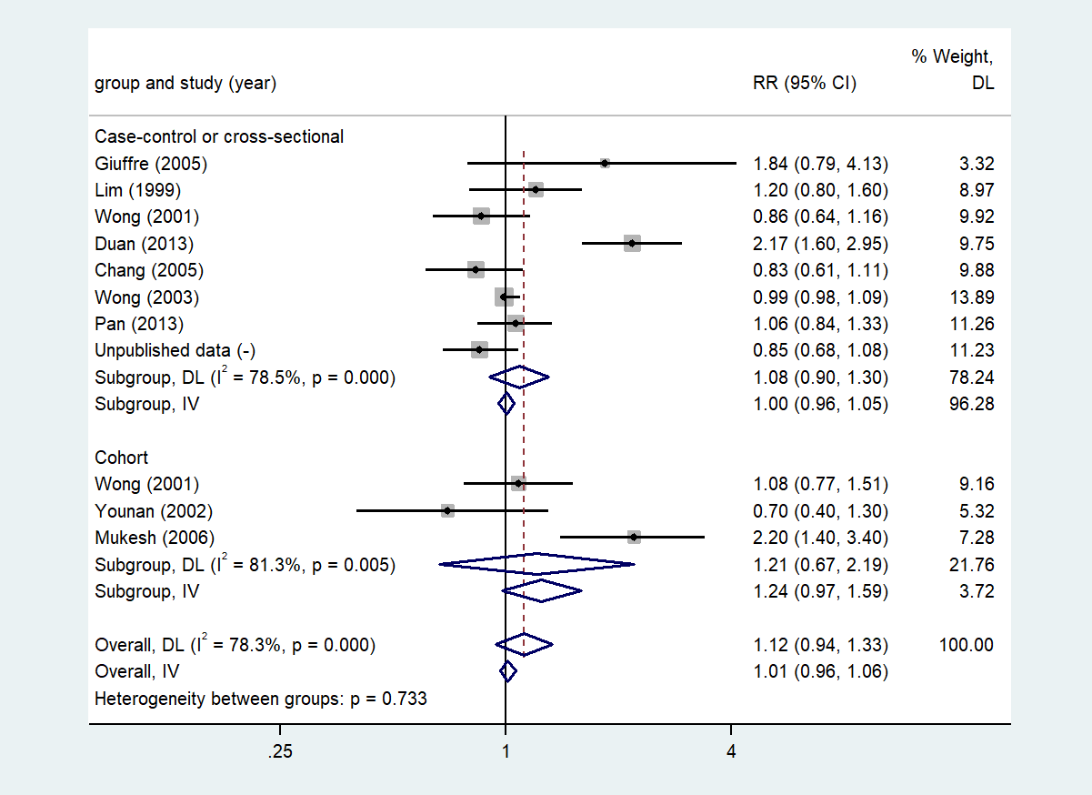


eFigure 3.3. Any myopia vs no myopia on posterior capsular cataract.


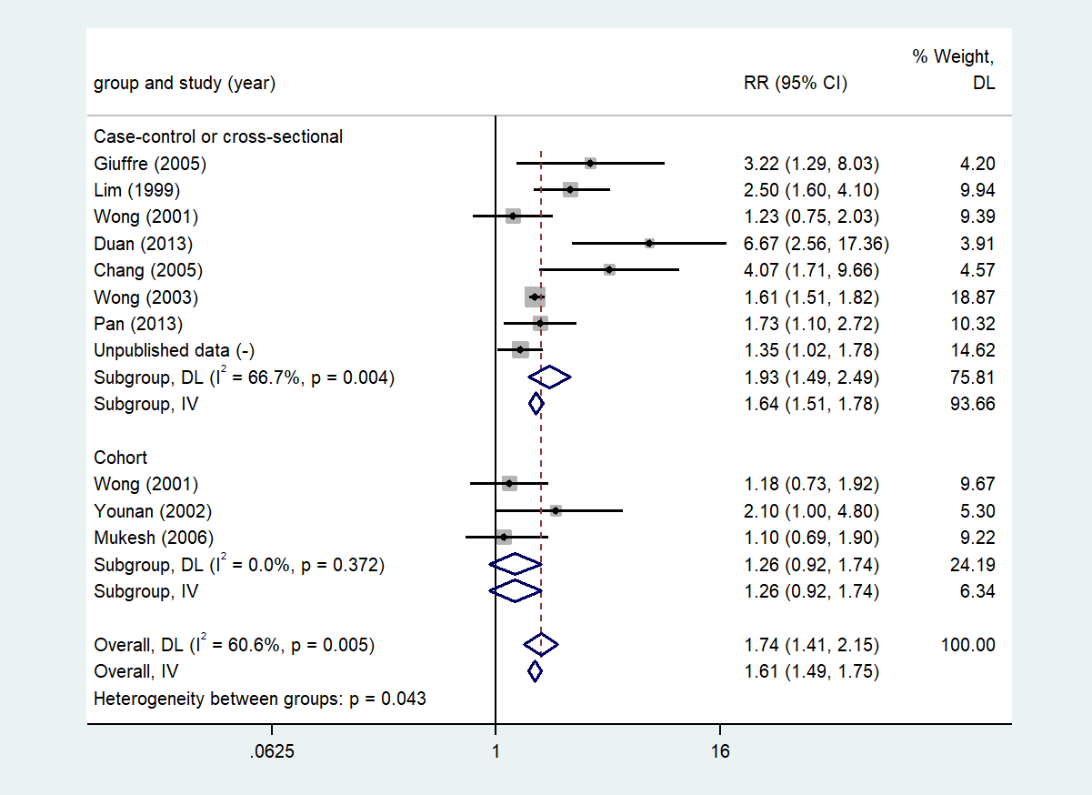


eFigure 4.1. Any myopia vs emmetropia on age-related macular degeneration.


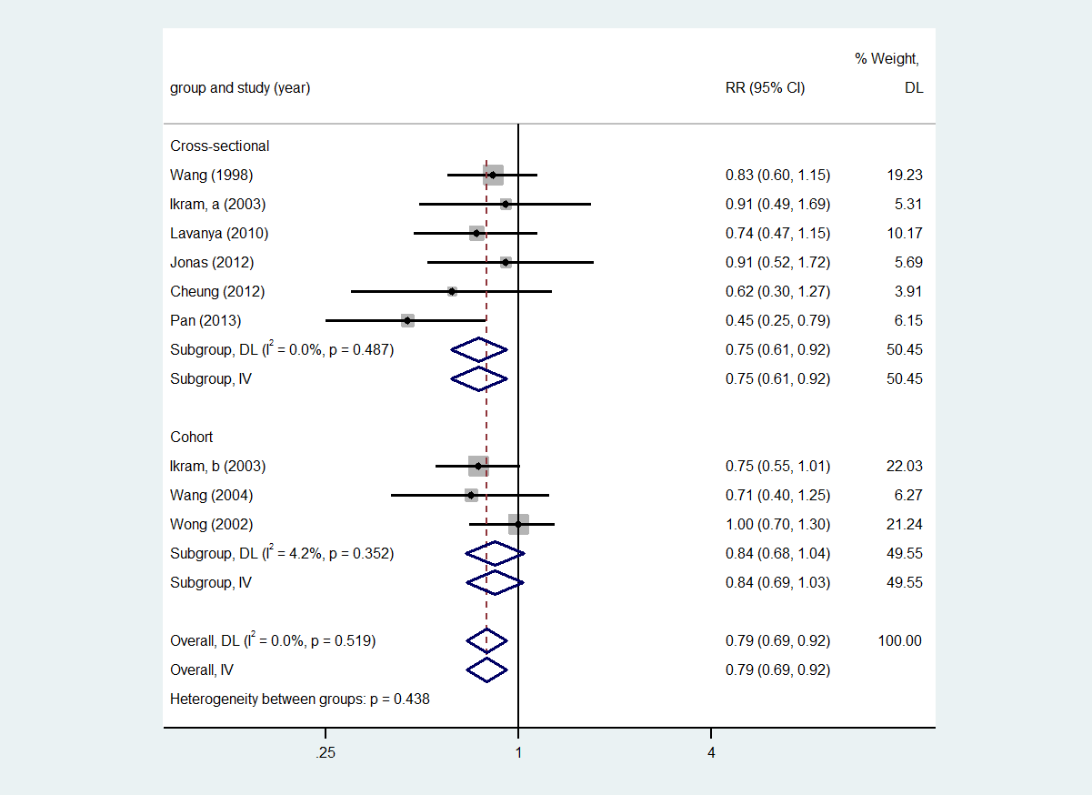


eFigure 4.2. Hyperopia vs emmetropia on age-related macular degeneration.


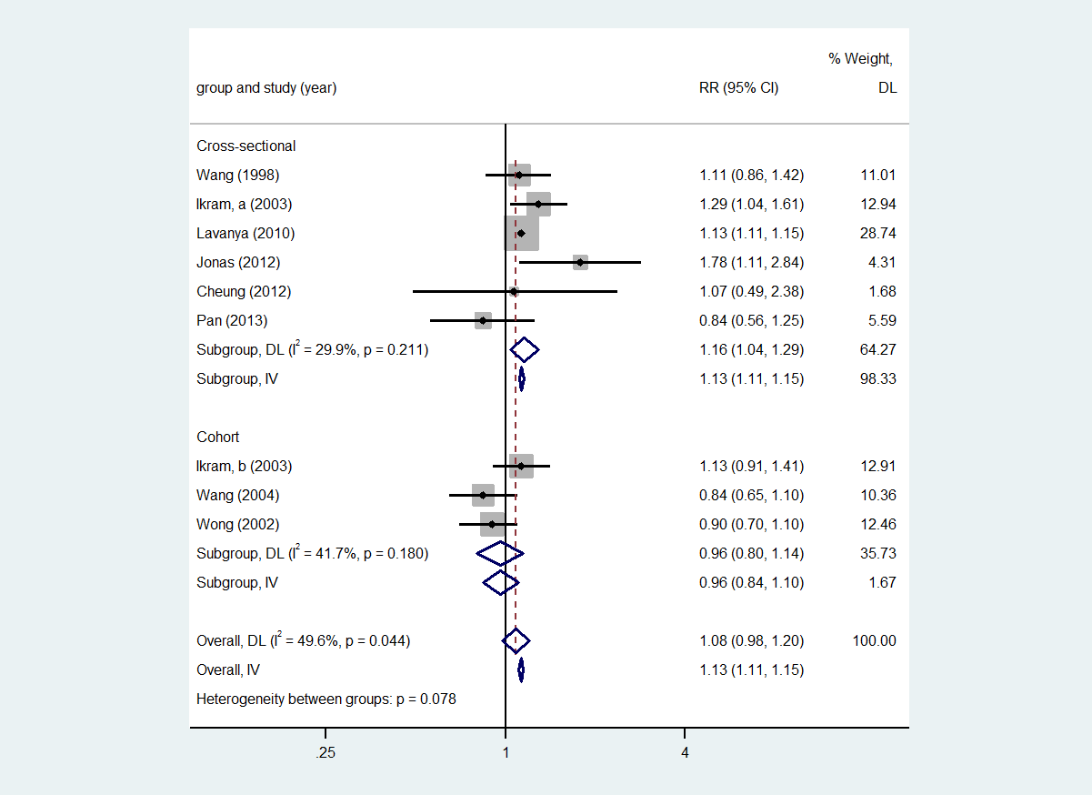


eFigure 4.3. Axial length (per millimeter increase) on age-related macular degeneration.


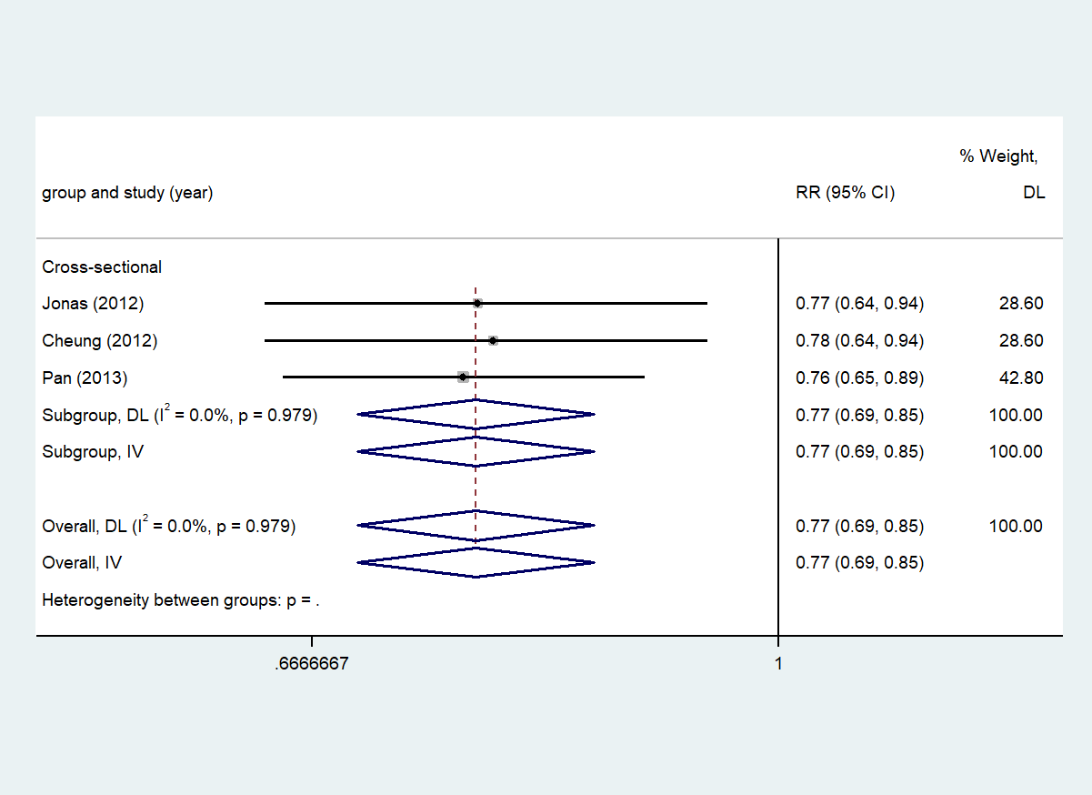


eFigure 4.4. Spherical equivalent (per diopter increase) on age-related macular degeneration.


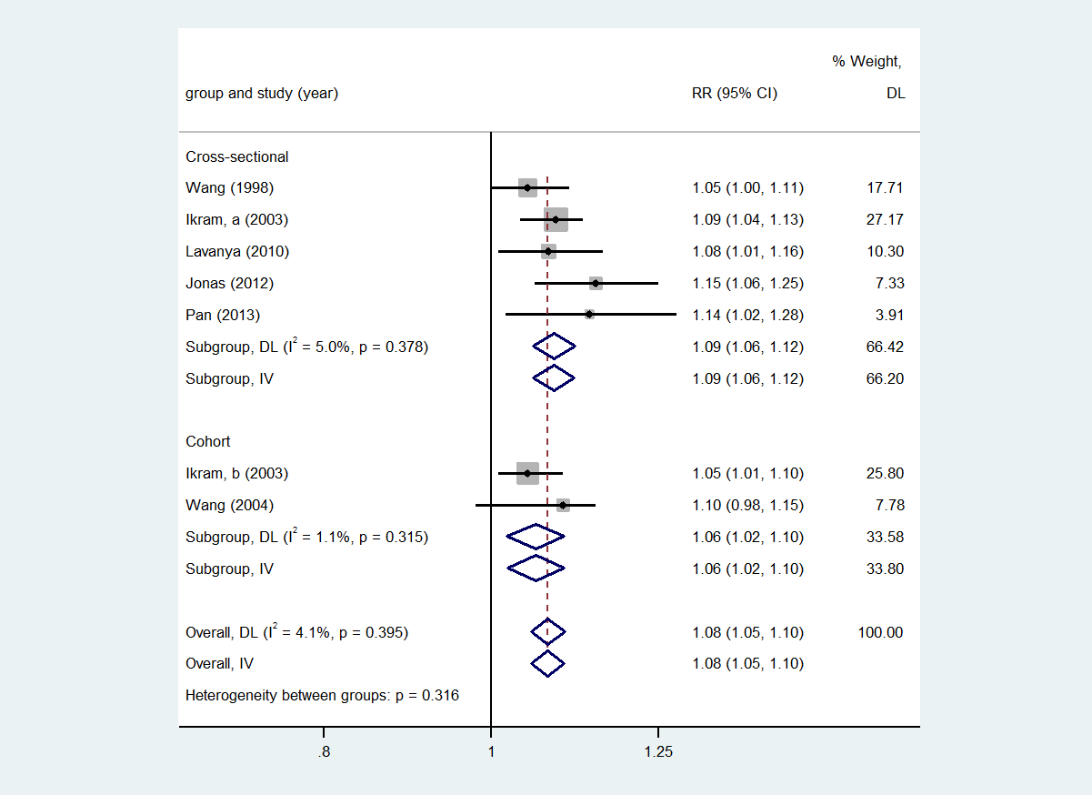


eFigure 5.1. Any myopia vs emmetropia on early age-related macular degeneration.


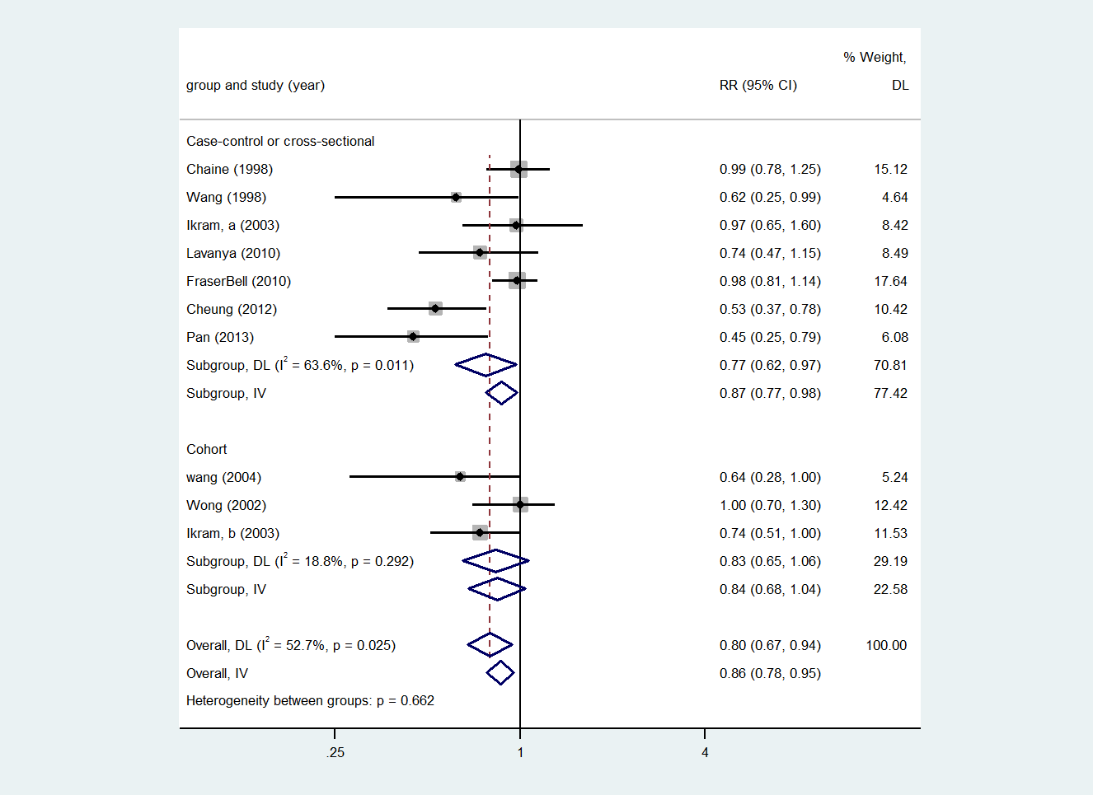


eFigure 5.2. Hyperopia vs emmetropia on early age-related macular degeneration.


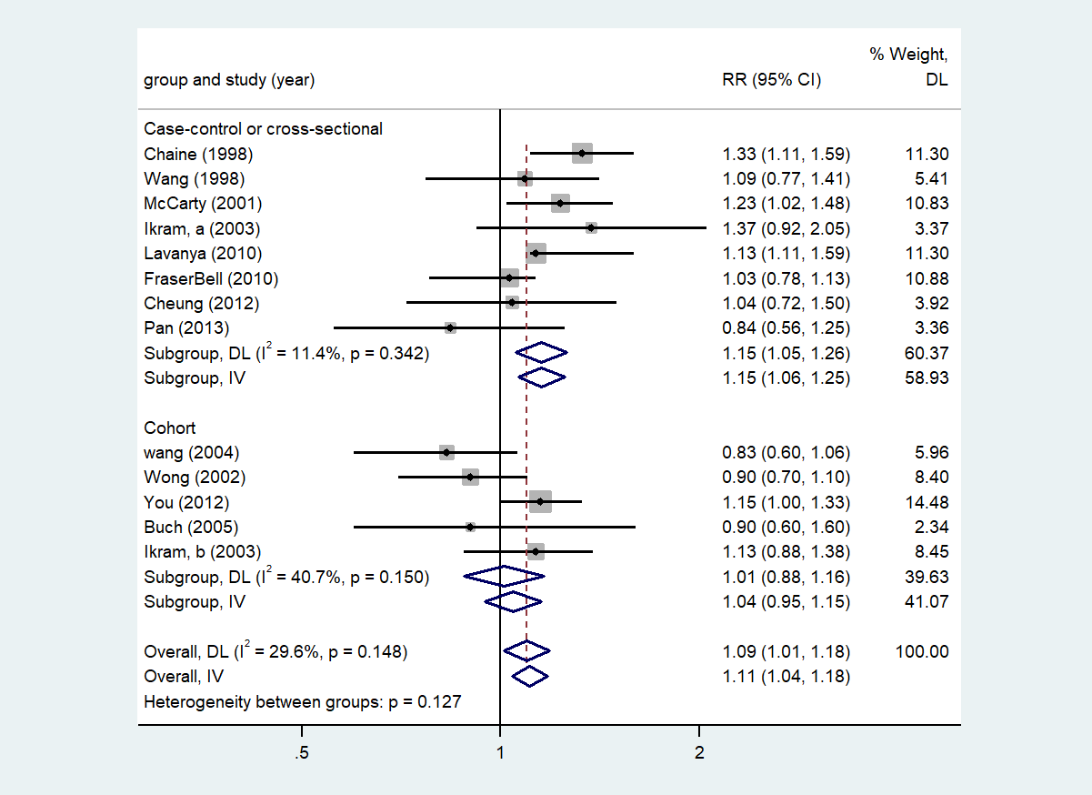


eFigure 5.3. Axial length (per millimeter increase) on early age-related macular degeneration.


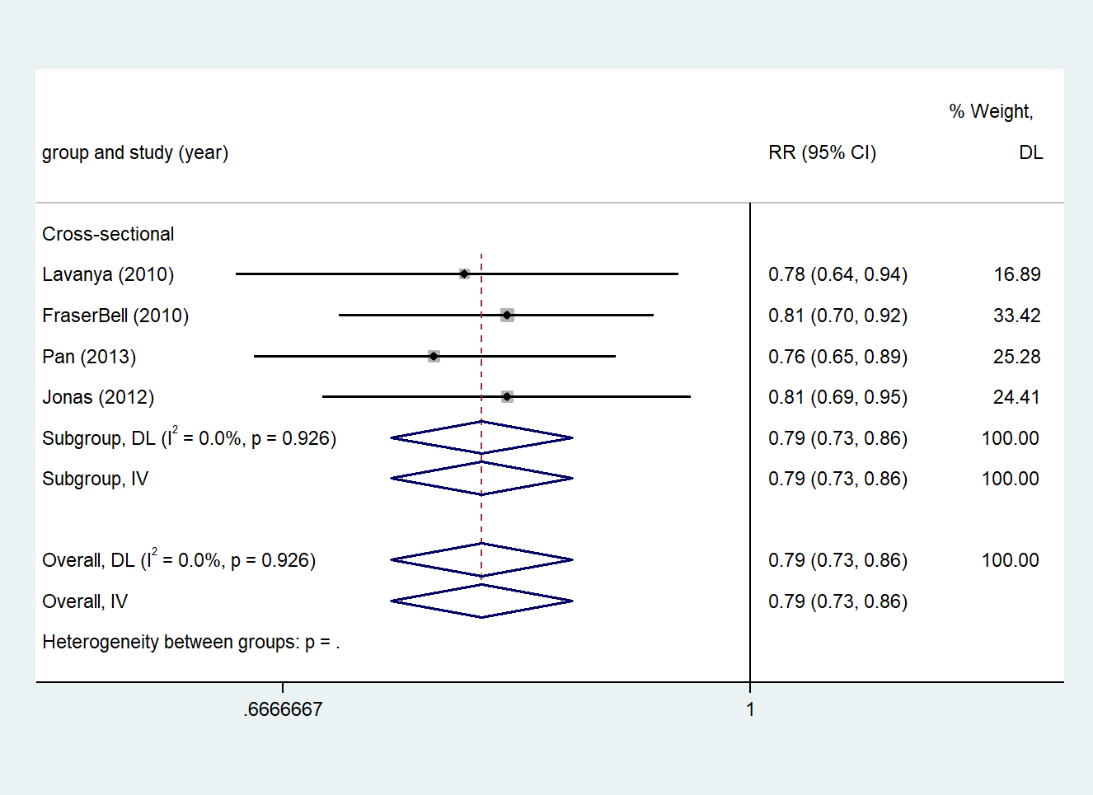


eFigure 5.4. Spherical equivalent (per diopter increase) on early age-related macular degeneration.


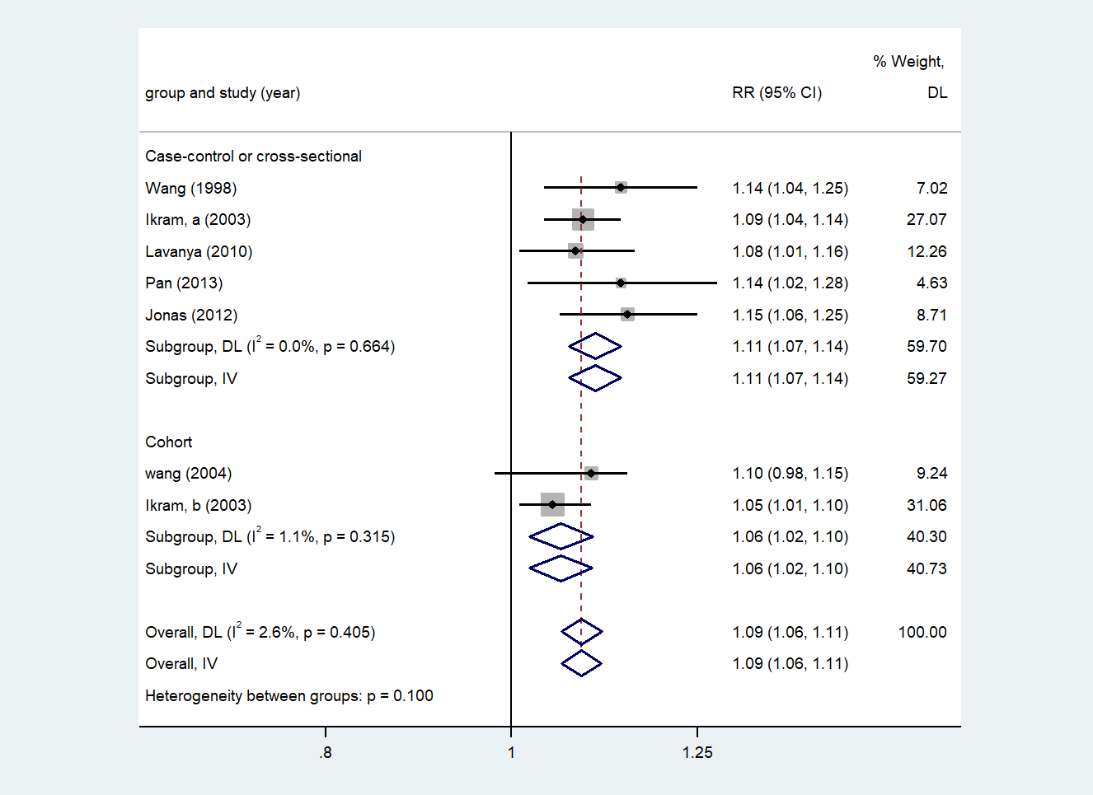


eFigure 5.5. Any myopia vs emmetropia on late age-related macular degeneration.


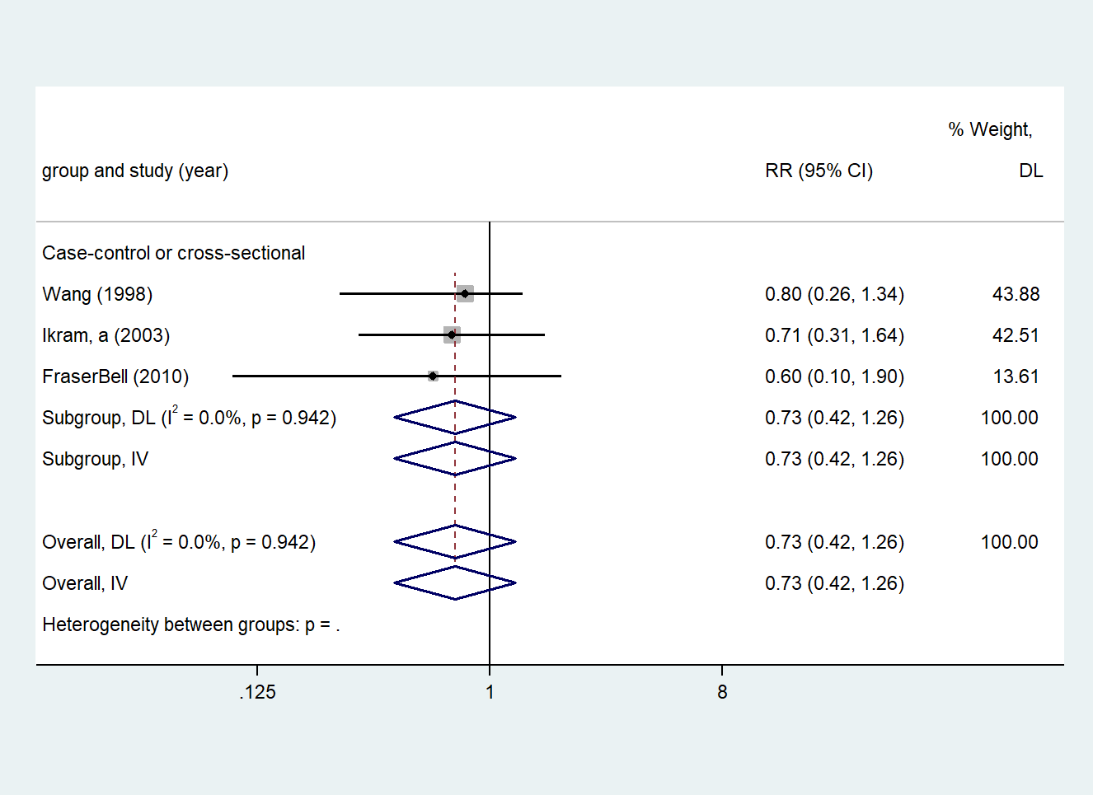


eFigure 5.6. Hyperopia vs emmetropia on late age-related macular degeneration.


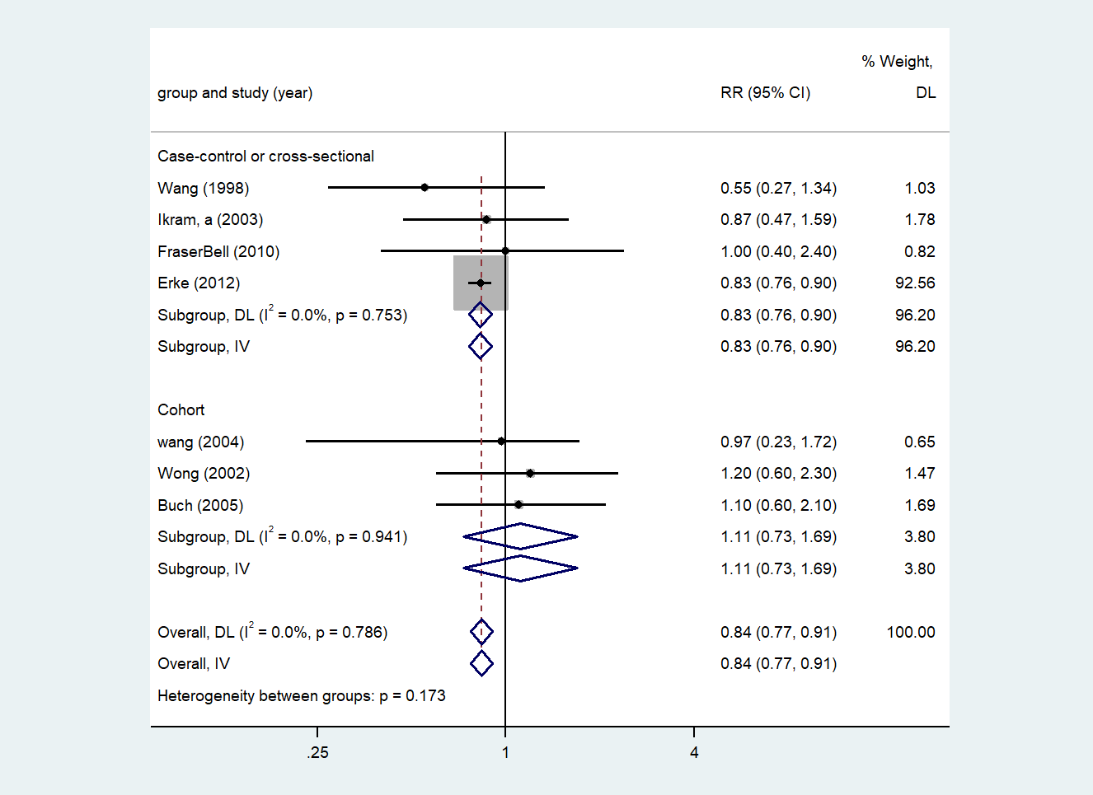


eFigure 5.7. Axial length (per millimeter increase) on late age-related macular degeneration.


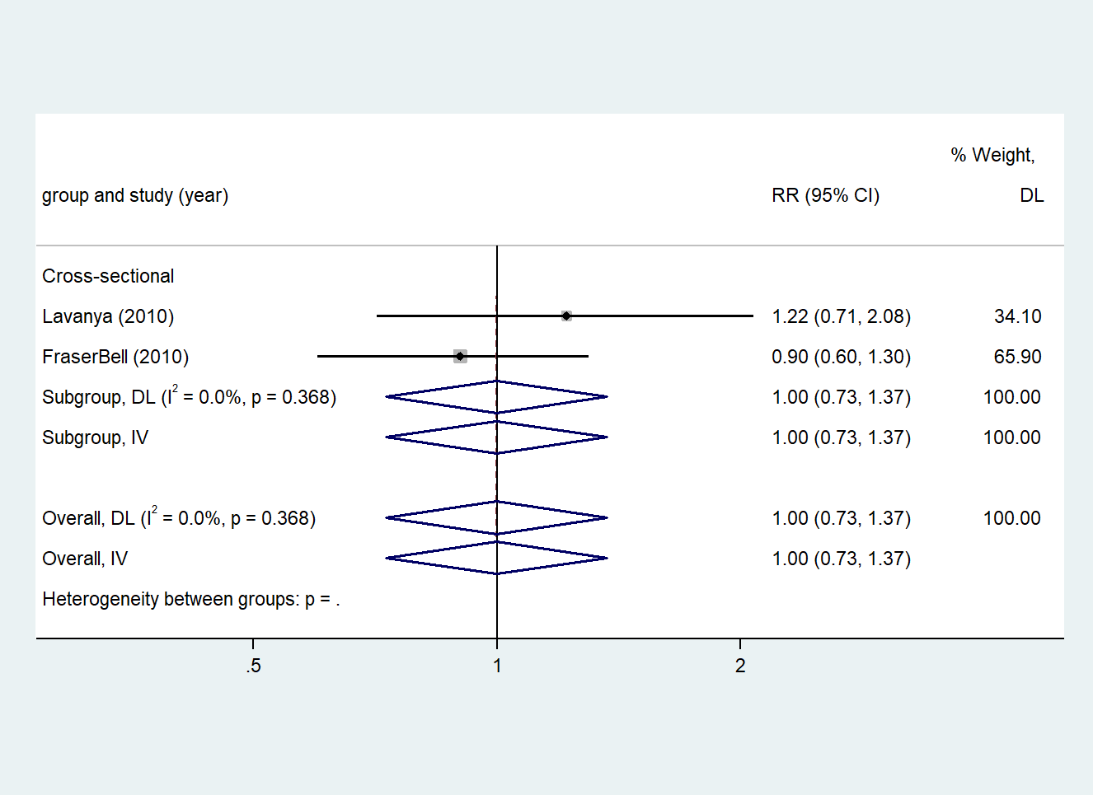


eFigure 5.8. Spherical equivalent (per diopter increase) on late age-related macular degeneration.


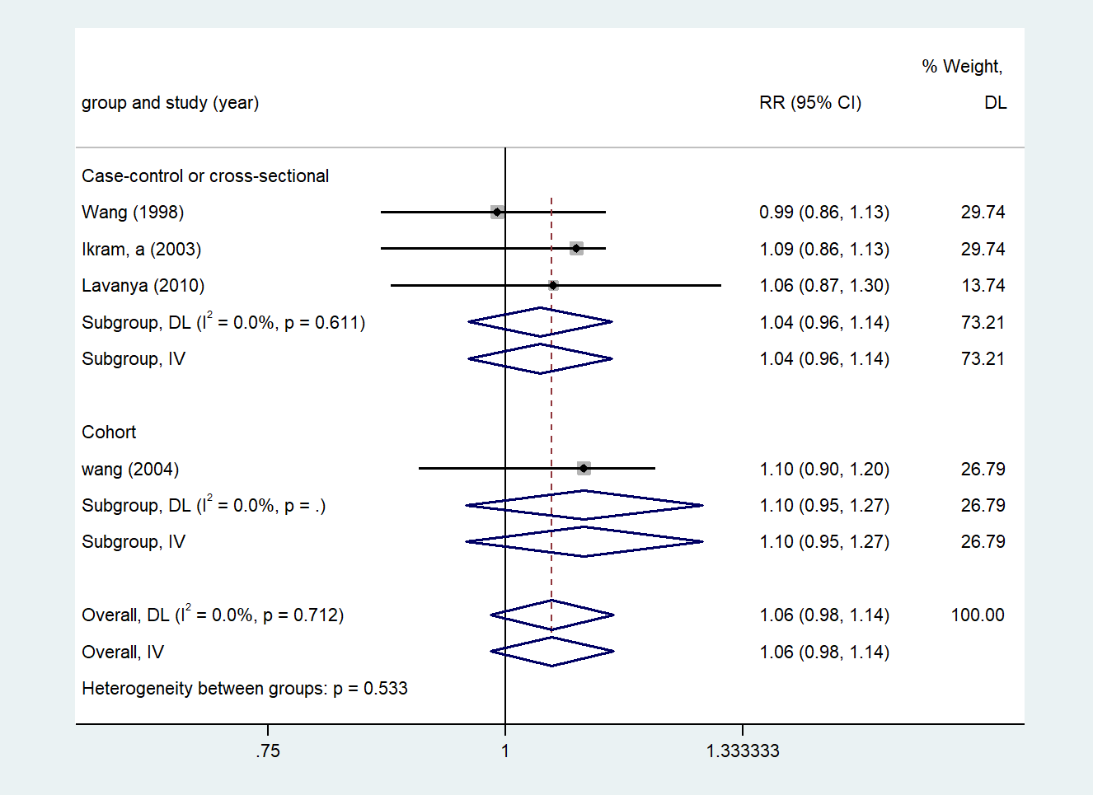


eFigure 6.1. Any myopia vs emmetropia on strabismus.


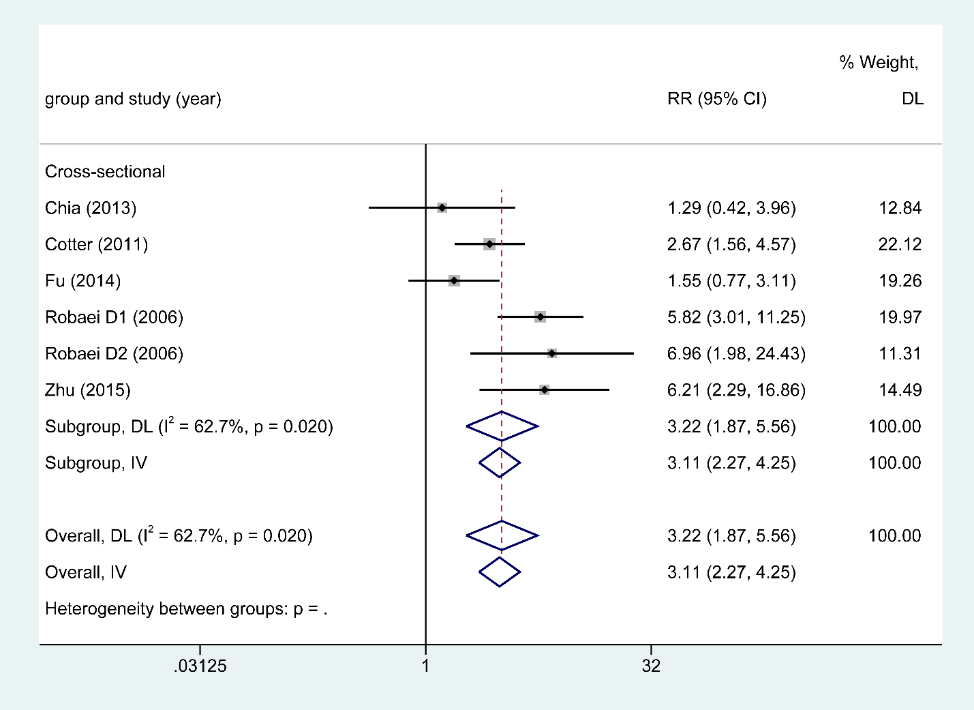


eFigure 6.2. Any myopia vs emmetropia on exotropia.


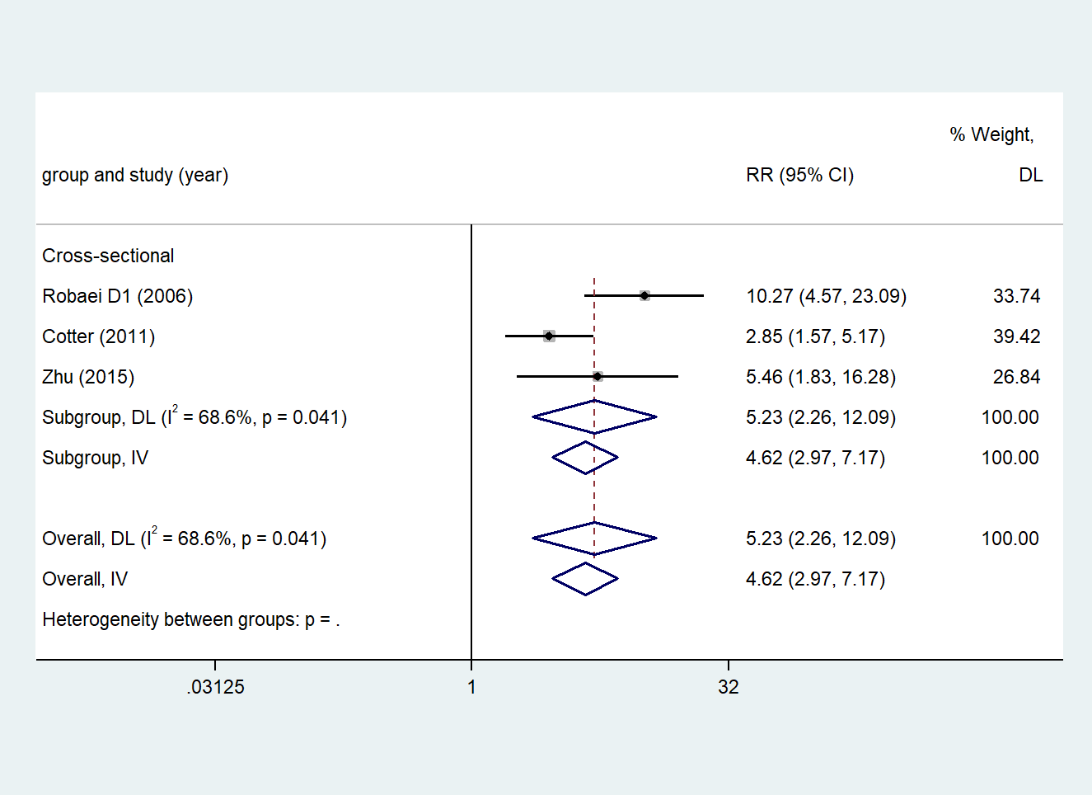


eFigure 6.3. Any myopia vs emmetropia on esotropia.


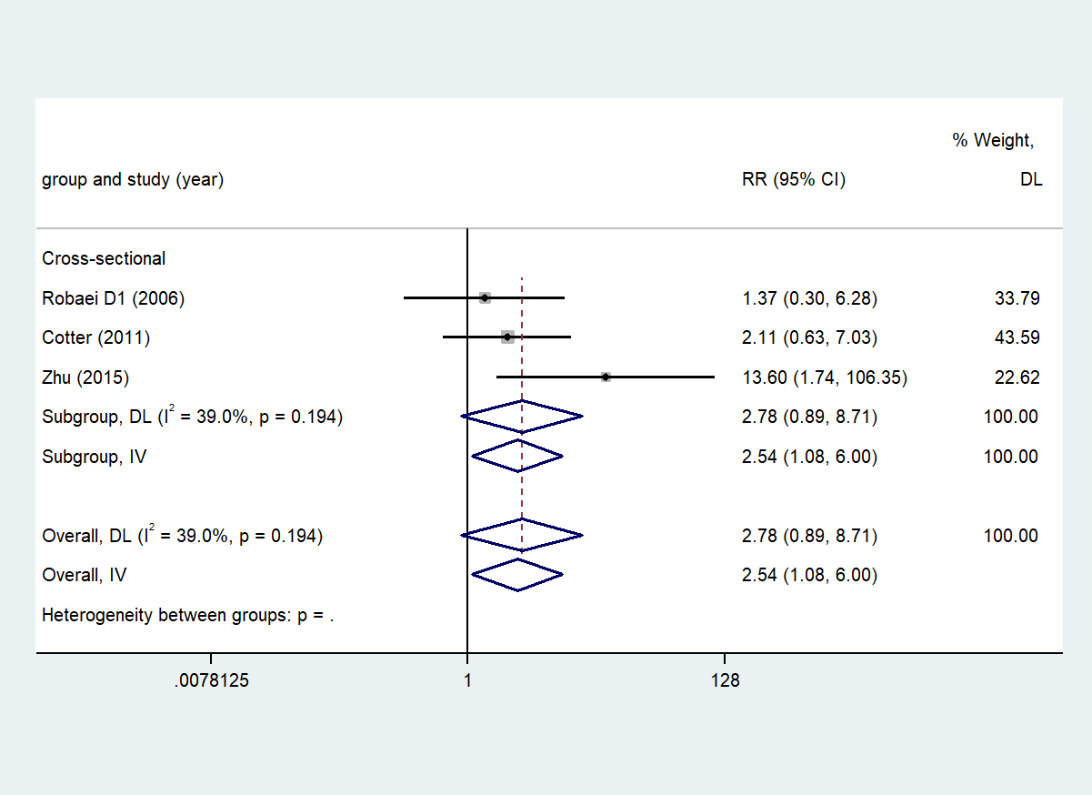


eFigure 6.4. Hyperopia vs emmetropia on strabismus.


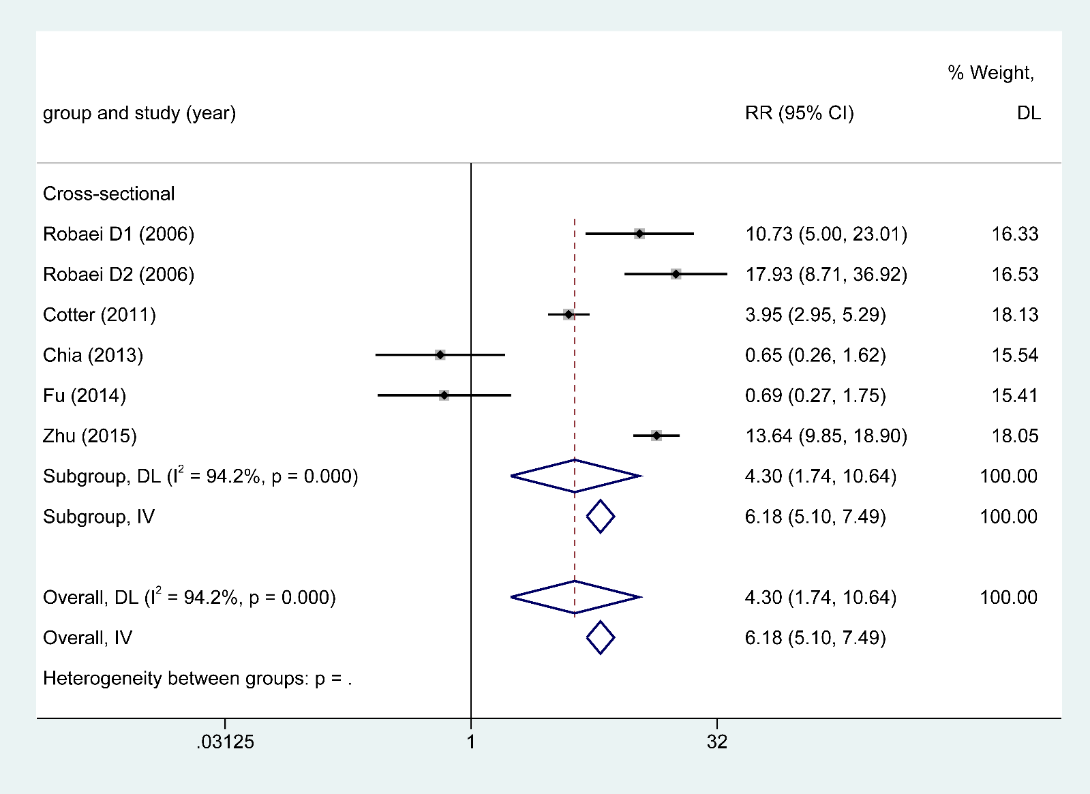


eFigure 6.5. Hyperopia vs emmetropia on exotropia.


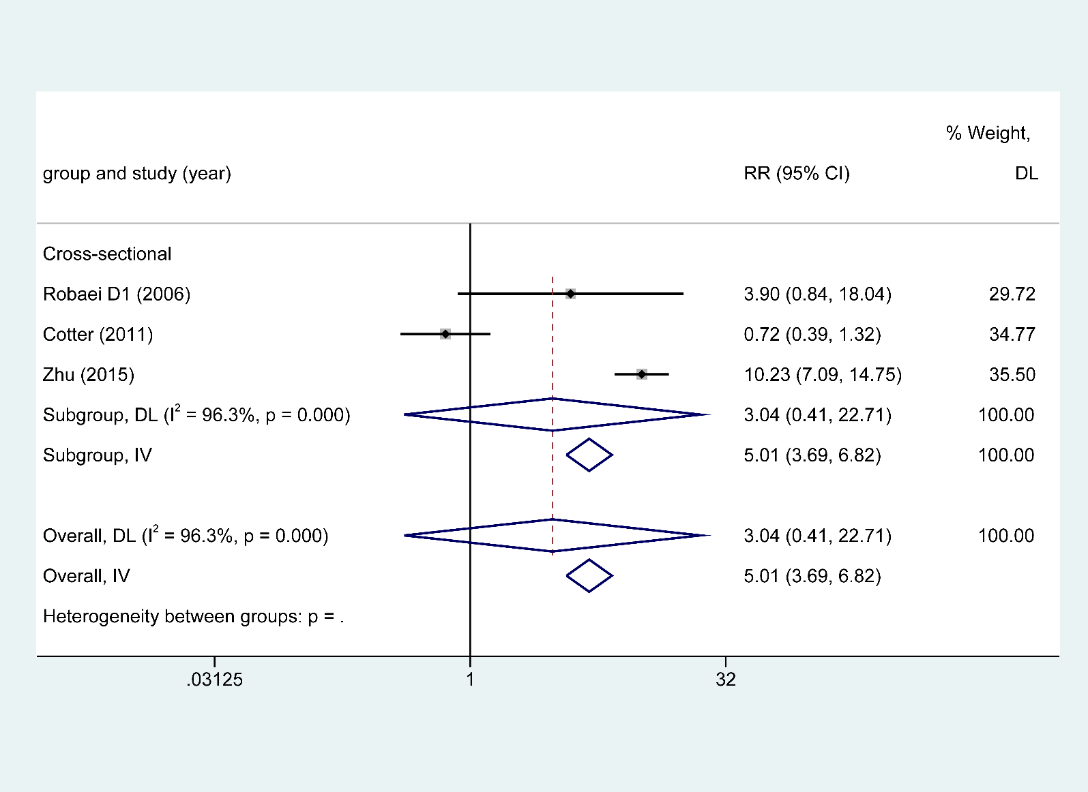


eFigure 6.6. Hyperopia vs emmetropia on esotropia.


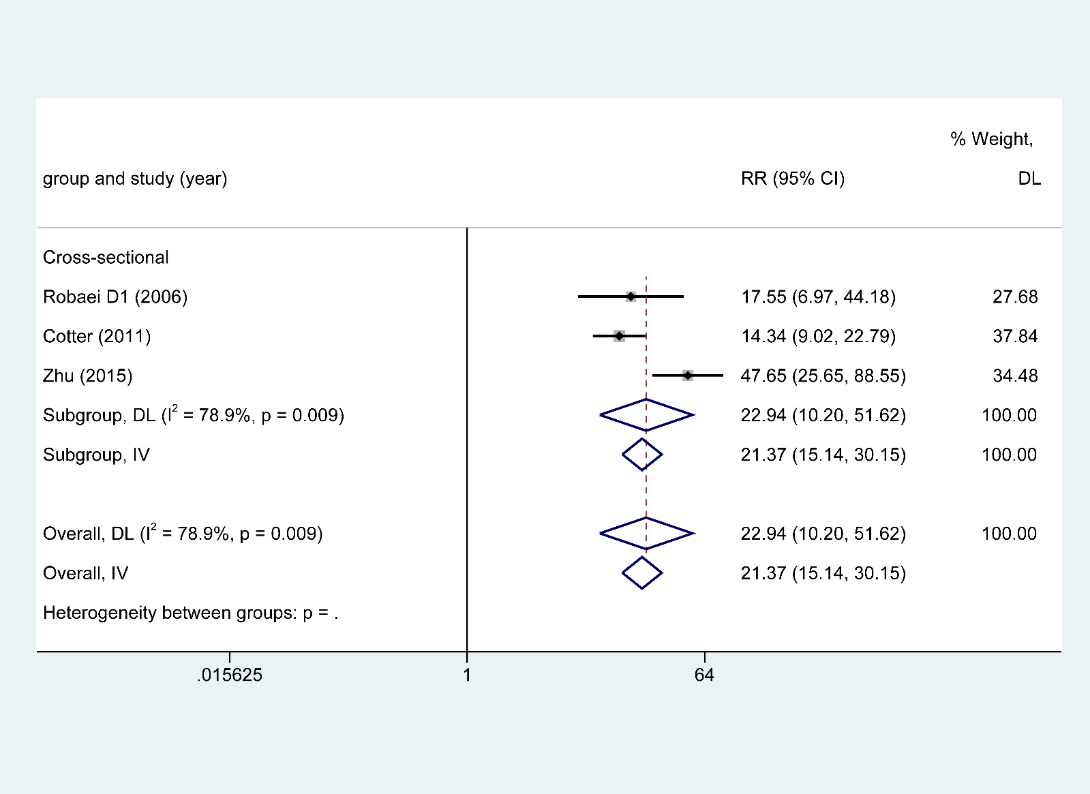


eFigure 6.7. Astigmatism vs emmetropia on strabismus.


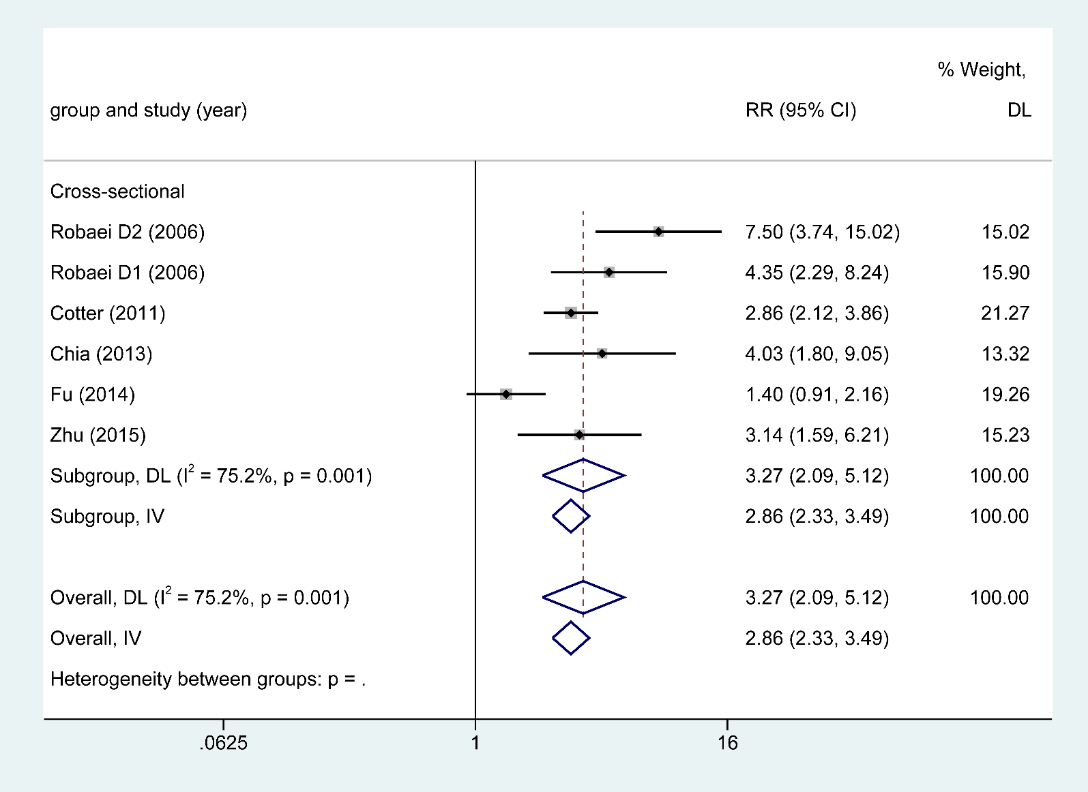


eFigure 6.8. Astigmatism vs emmetropia on exotropia.


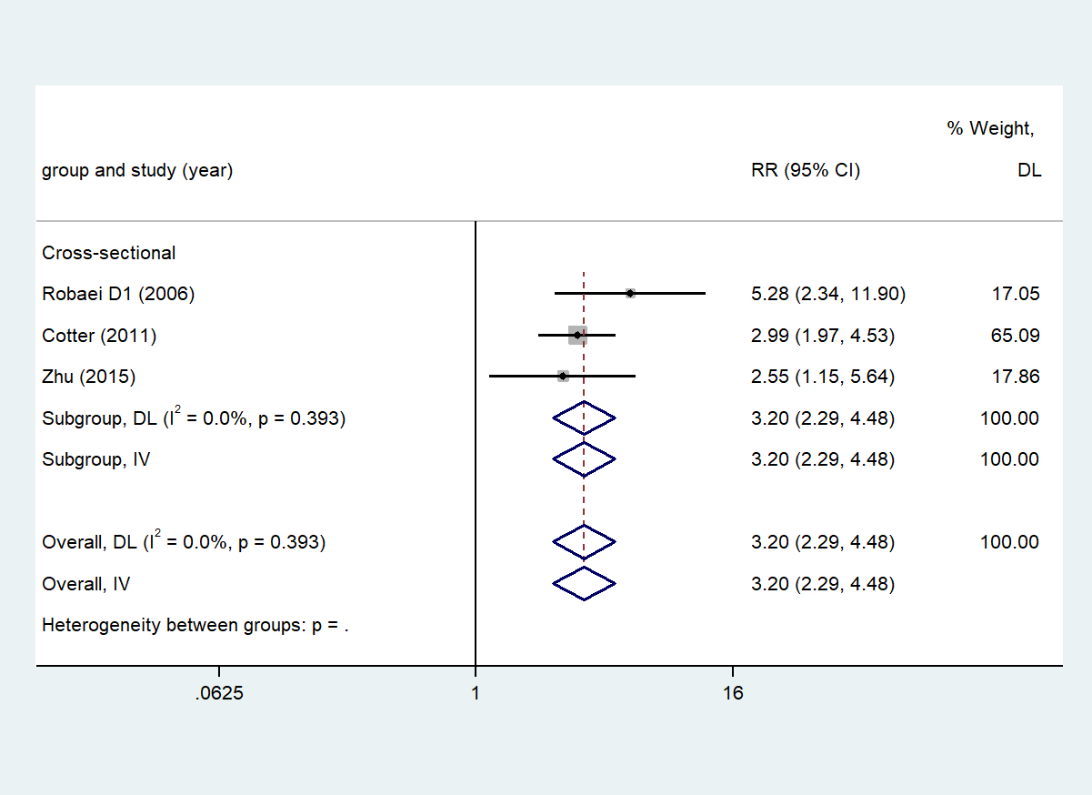


eFigure 6.9. Astigmatism vs emmetropia on esotropia.


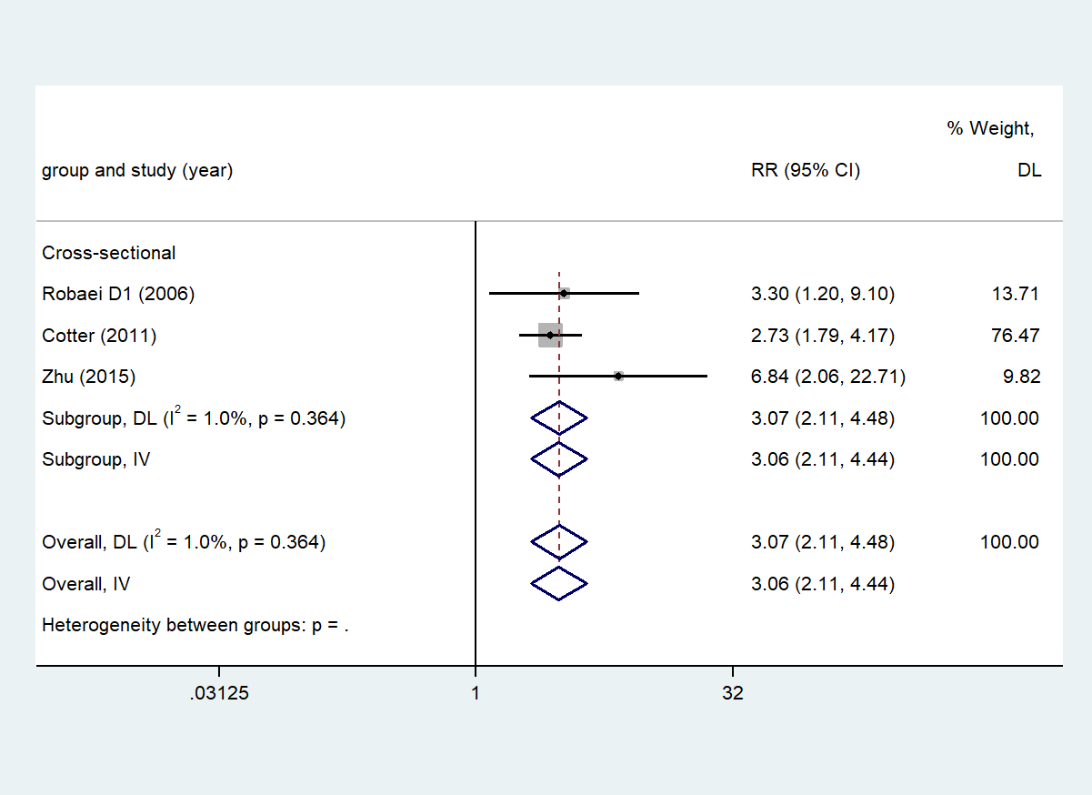


eFigure 6.10. Anisometropia vs emmetropia on strabismus.


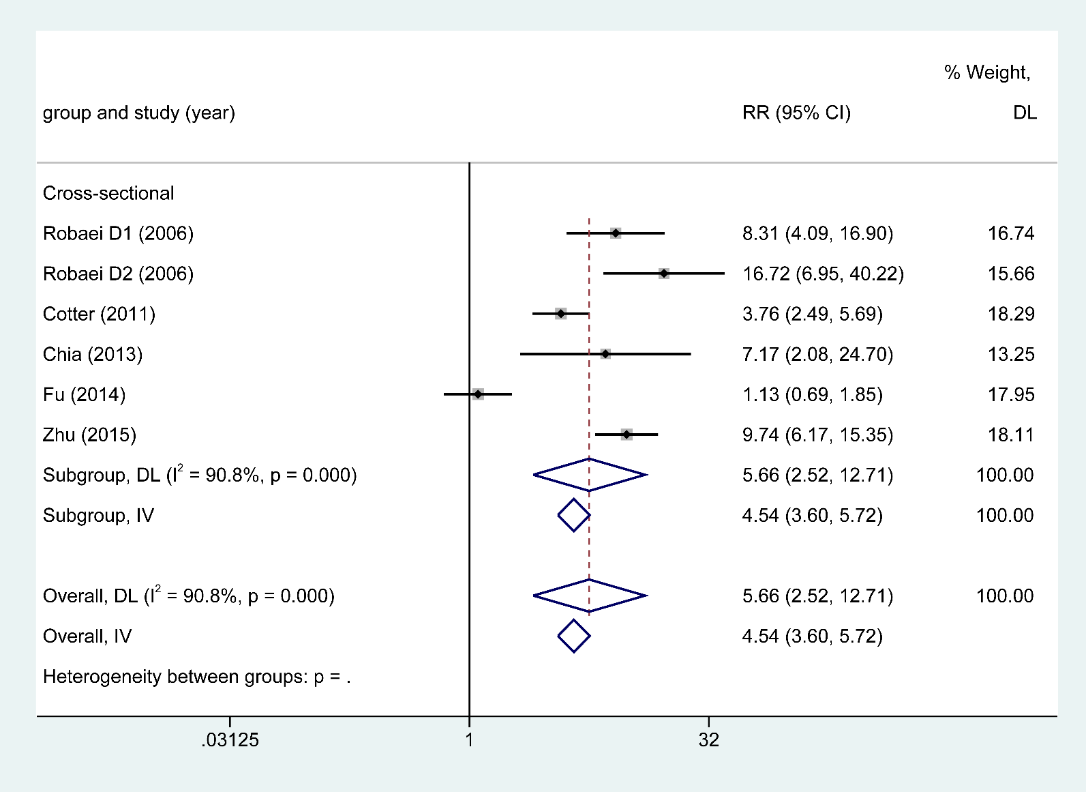


eFigure 6.11. Anisometropia vs emmetropia on exotropia.


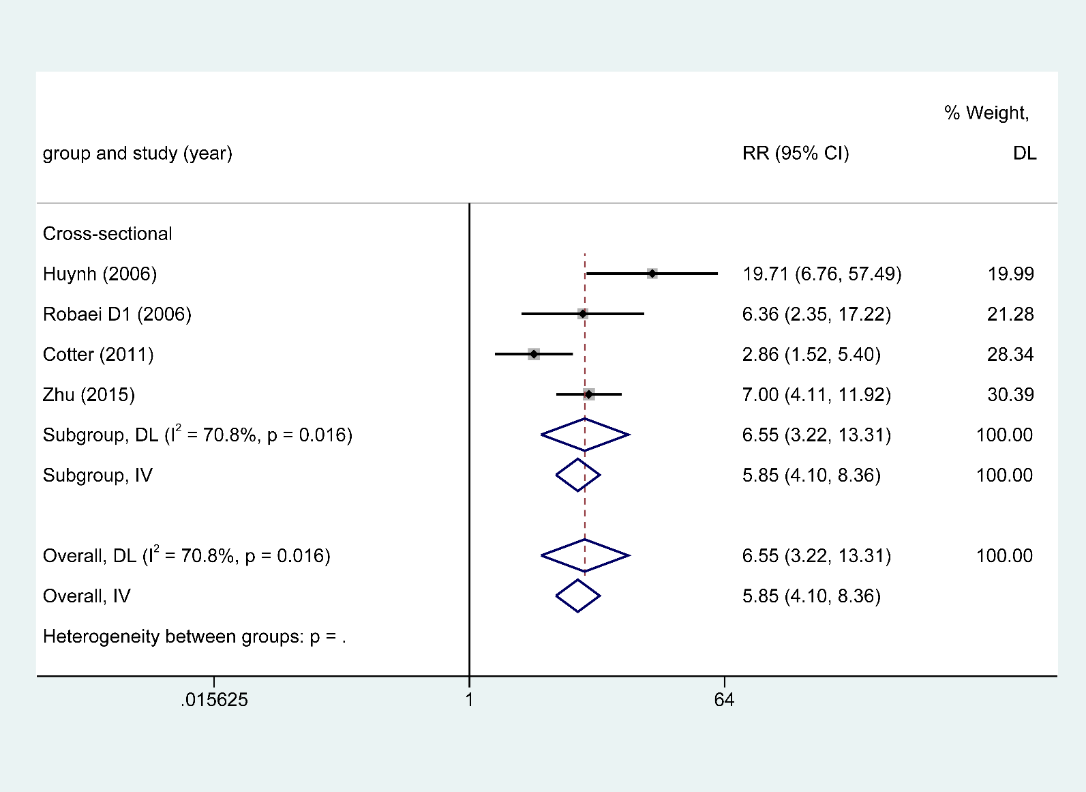


eFigure 6.12. Anisometropia vs emmetropia on esotropia.


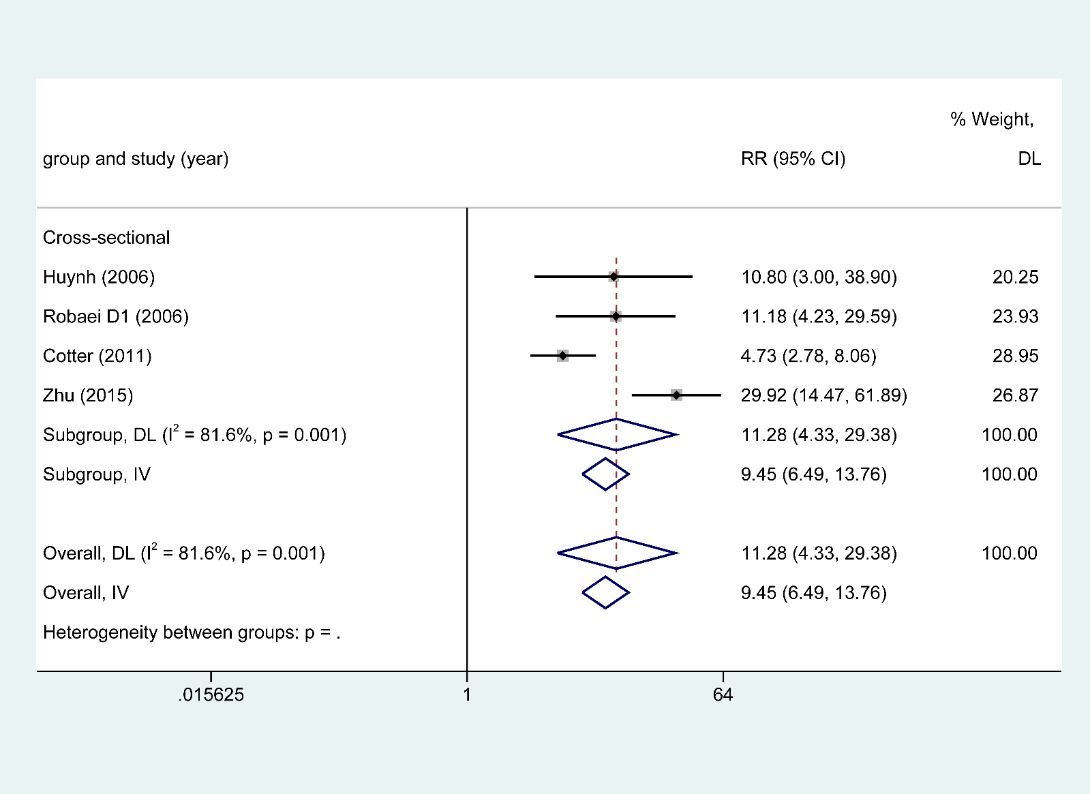


eFigure 7.1. Any myopia vs emmetropia on open-angle glaucoma.


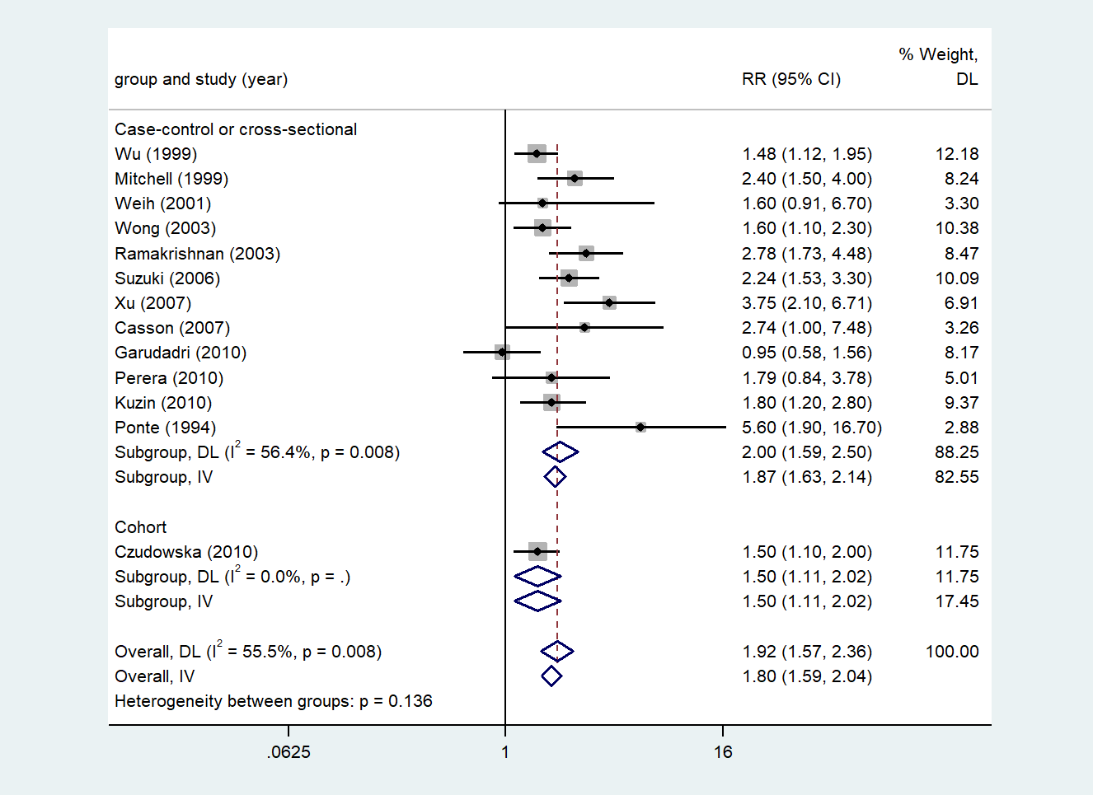


eFigure 7.2. Mild myopia vs emmetropia on open-angle glaucoma.


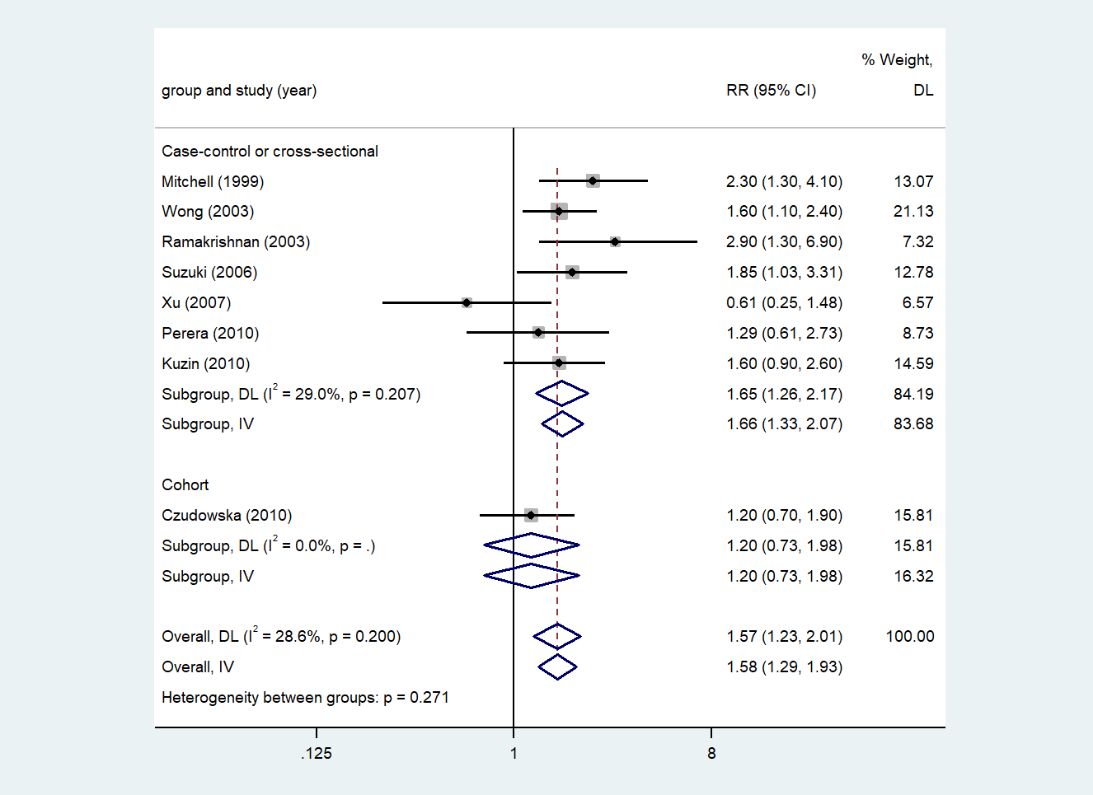


eFigure 7.3. Moderate/high myopia vs emmetropia on open-angle glaucoma.


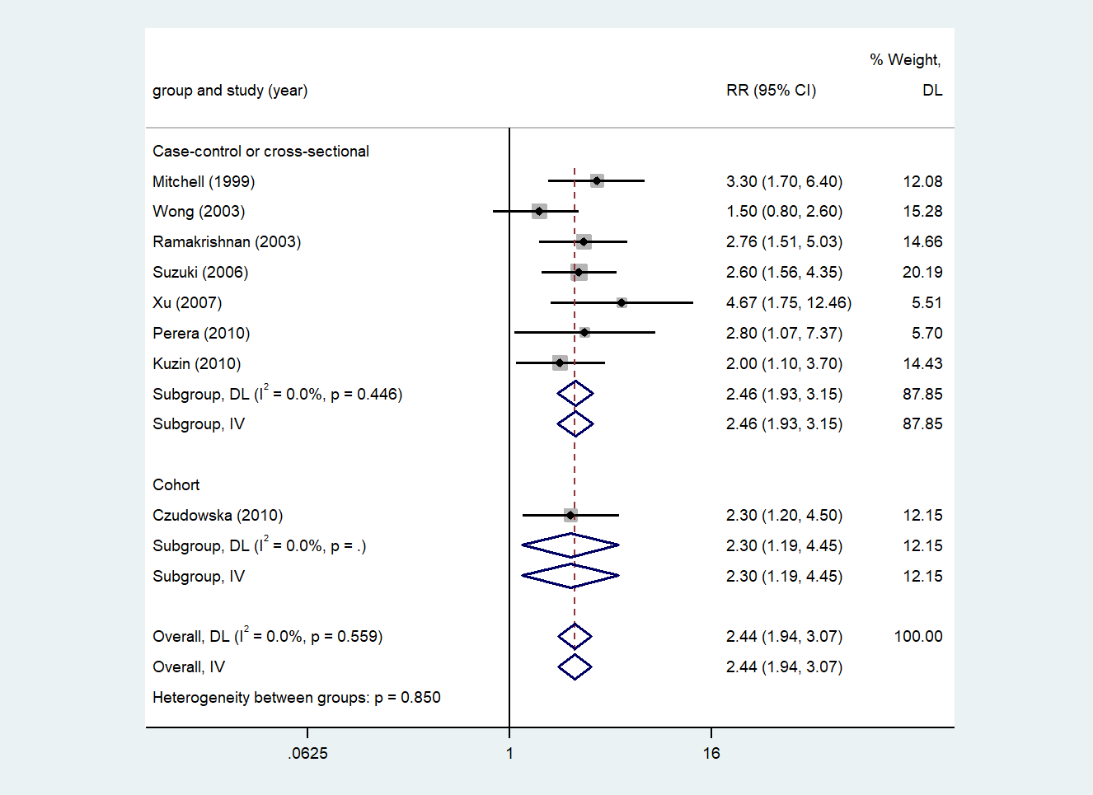


eFigure 8. High myopia vs no high myopia on retinal re-detachment after silicon oil removal.


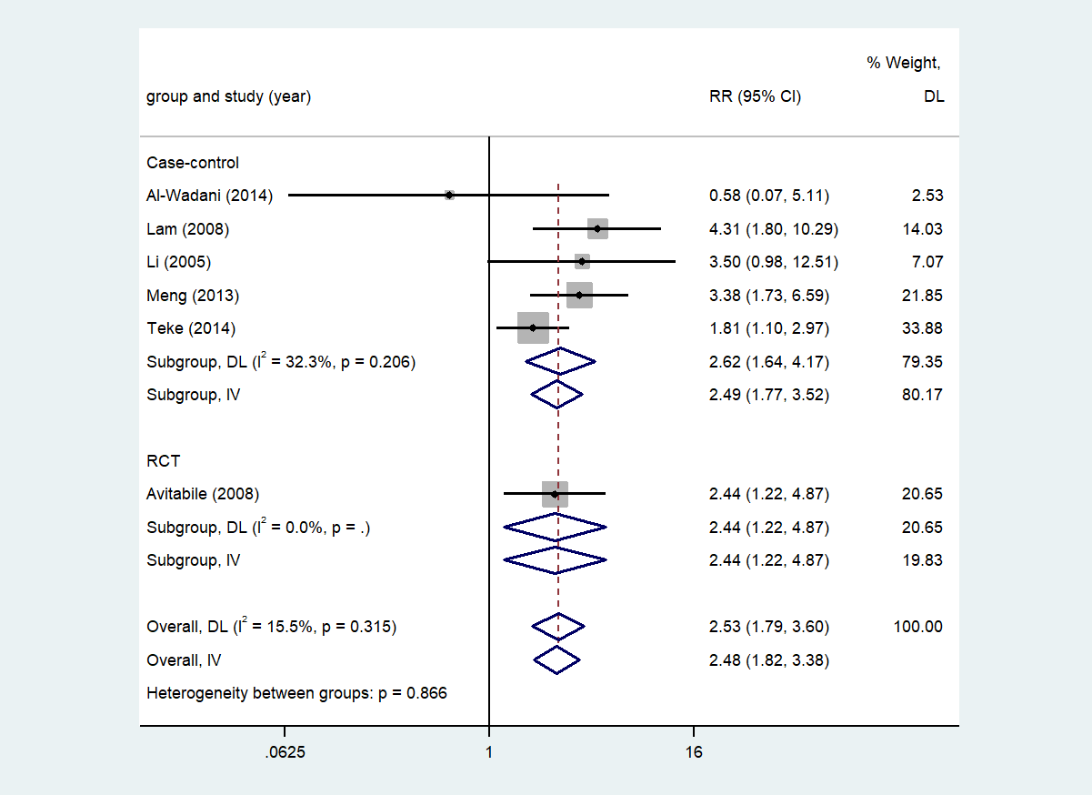


eFigure 9.1. Any myopia vs emmetropia on diabetic retinopathy.


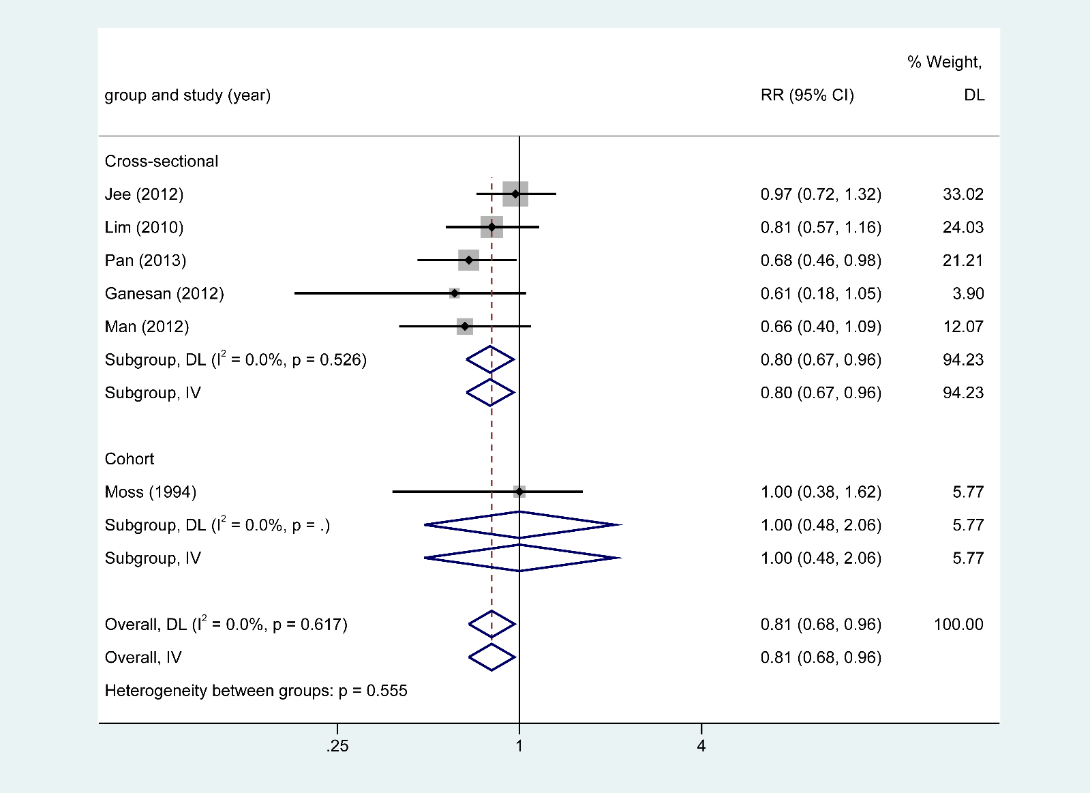


eFigure 9.2. Mild/moderate myopia vs emmetropia on diabetic retinopathy.


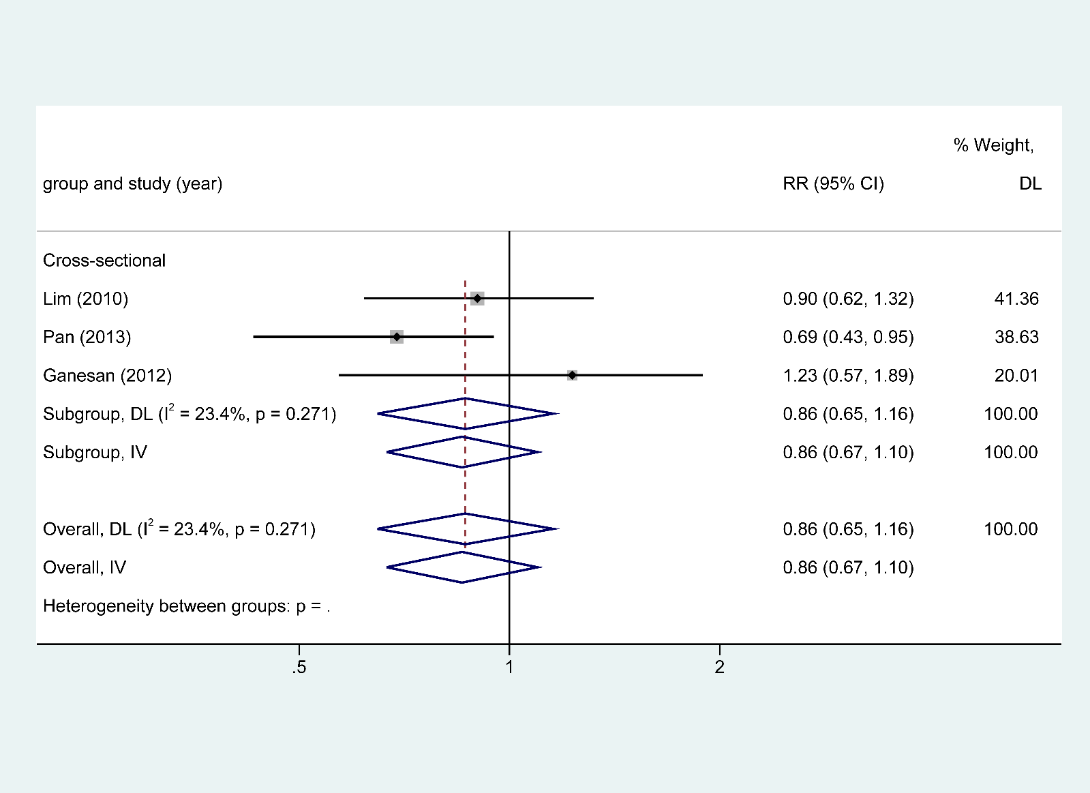


eFigure 9.3. High myopia vs emmetropia on diabetic retinopathy.


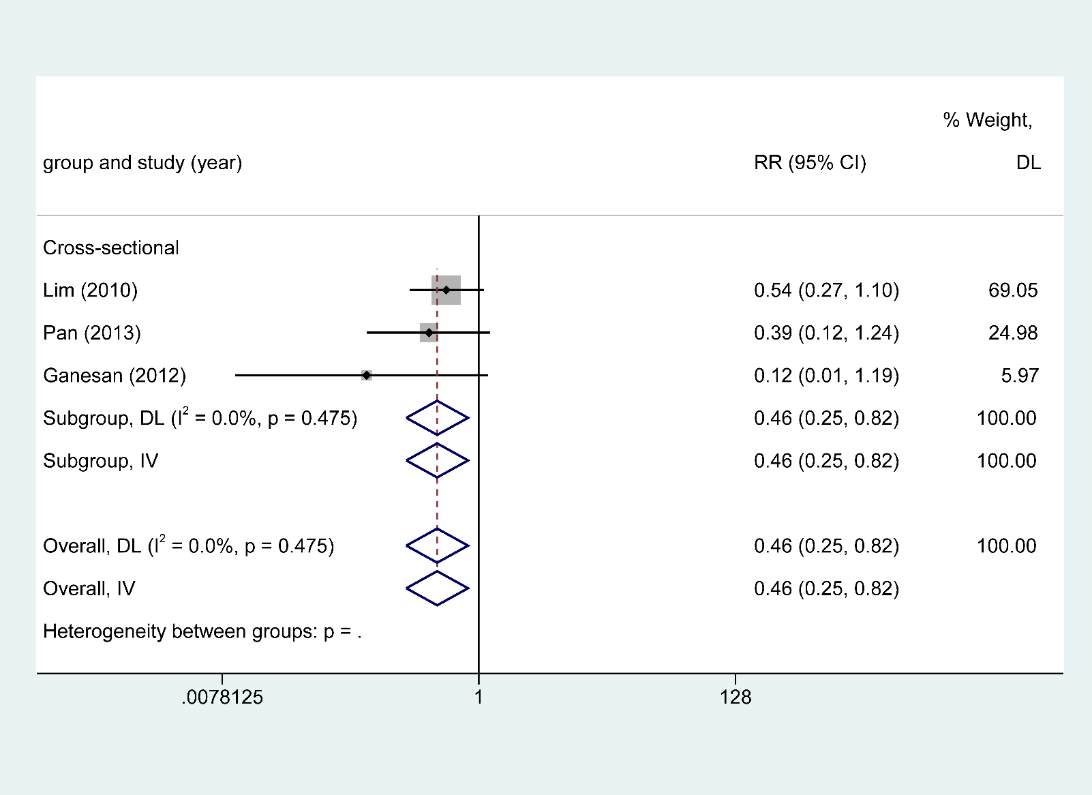


eFigure 9.4. Hyperopia vs emmetropia on diabetic retinopathy.


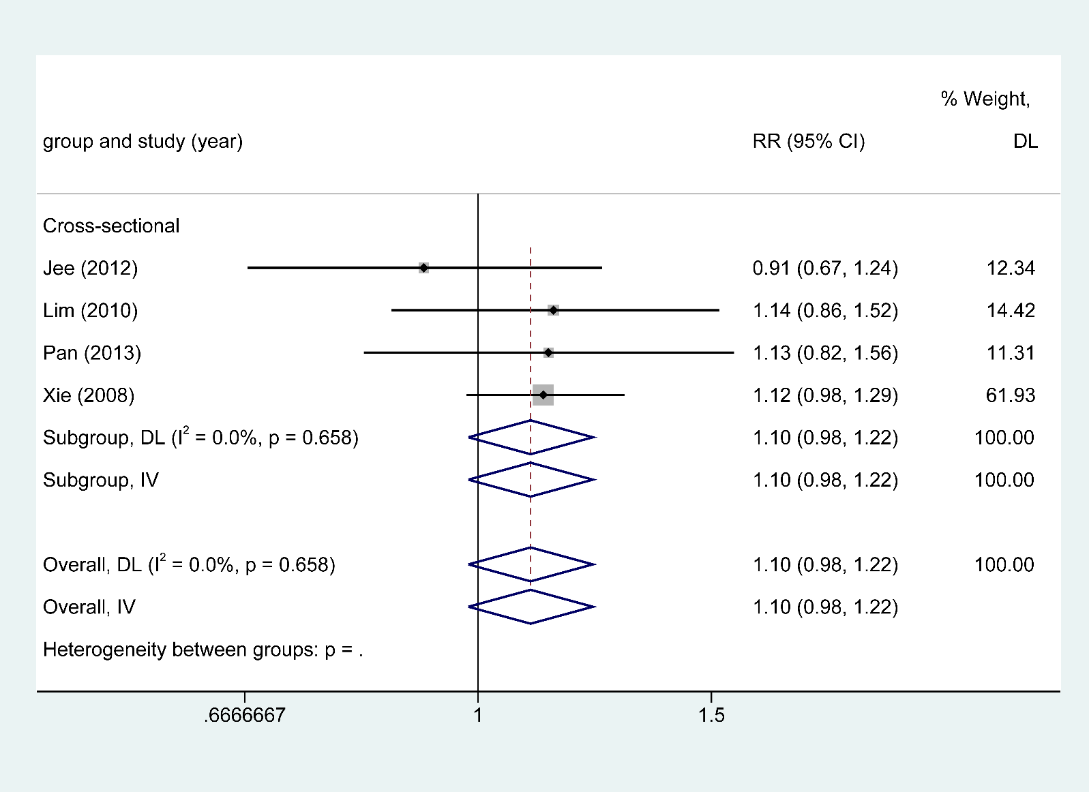


eFigure 9.5. Any myopia vs emmetropia on vision-threatening diabetic retinopathy.


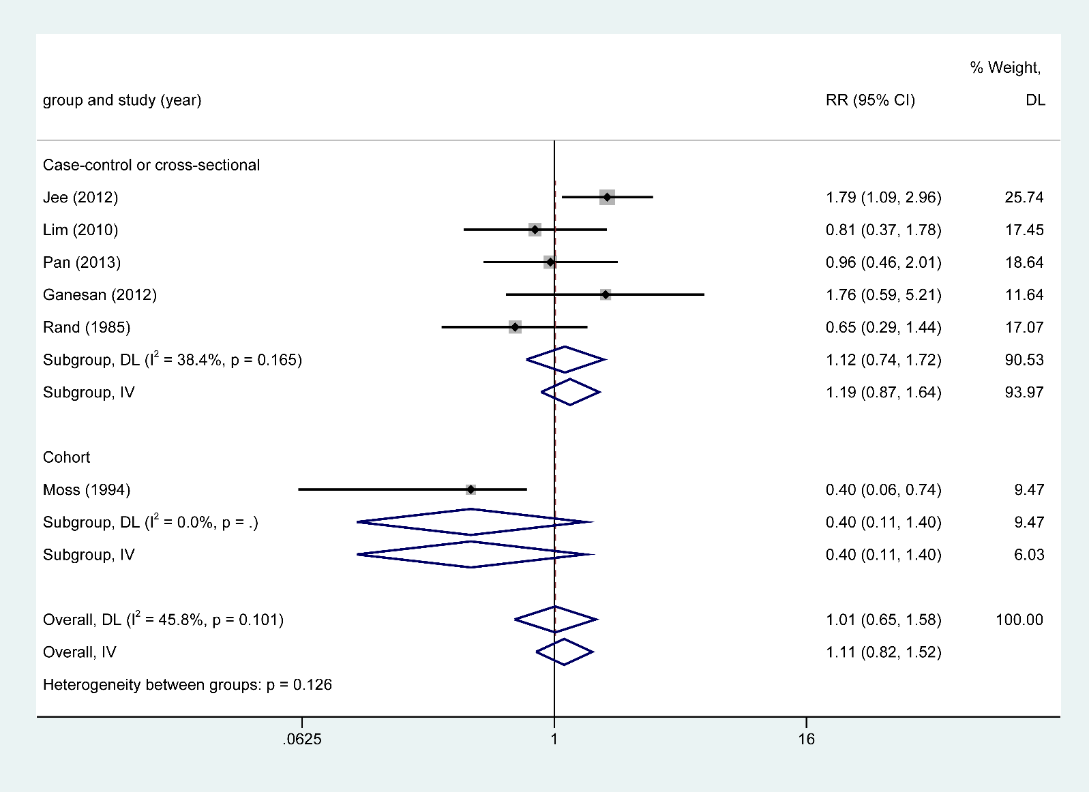


eFigure 10.1. Any myopia vs no myopia/emmetropia on open-angle glaucoma.


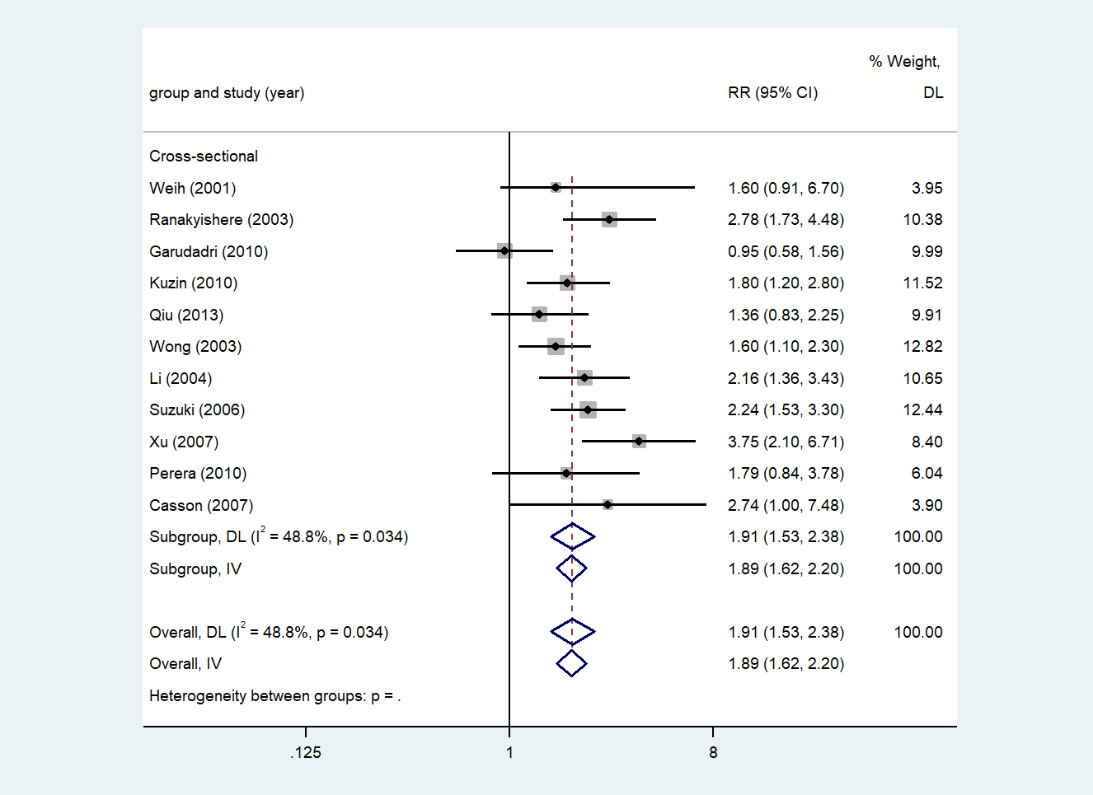


eFigure 10.2. Mild myopia vs no myopia/emmetropia on open-angle glaucoma.


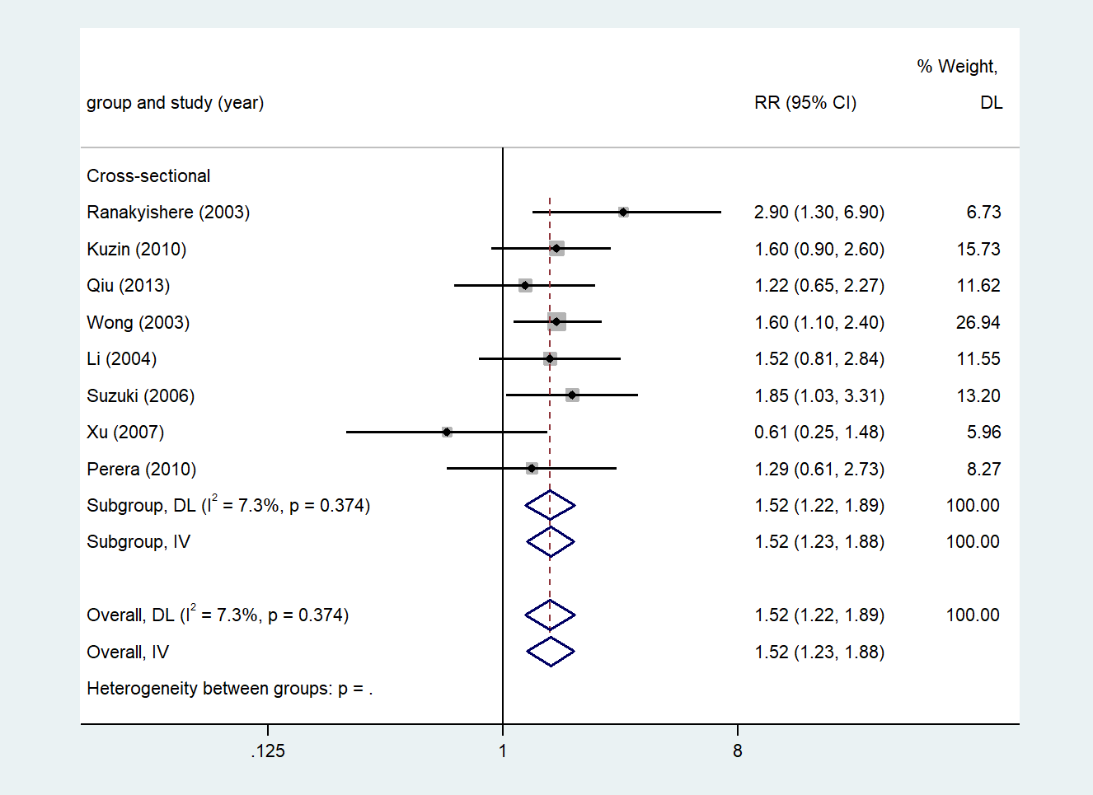


eFigure 10.3. Moderate/high myopia vs no myopia/emmetropia on open-angle glaucoma.


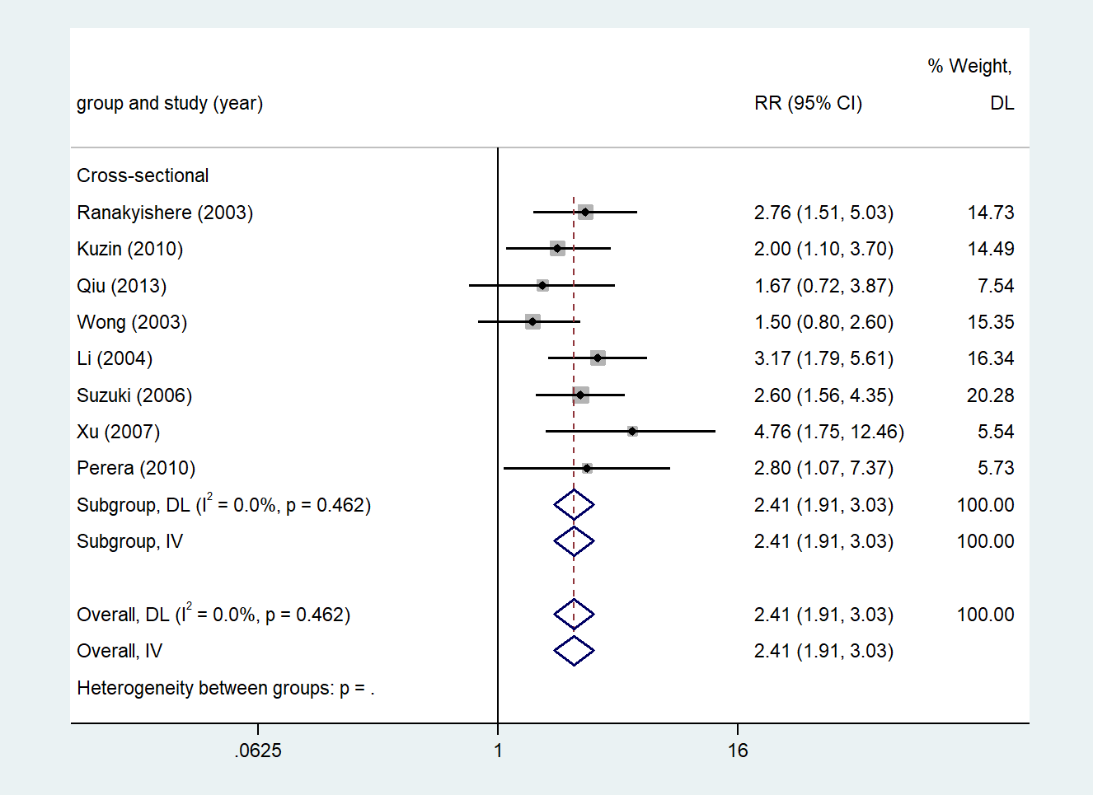


eFigure 11.1. Any myopia vs no myopia on open-angle glaucoma.


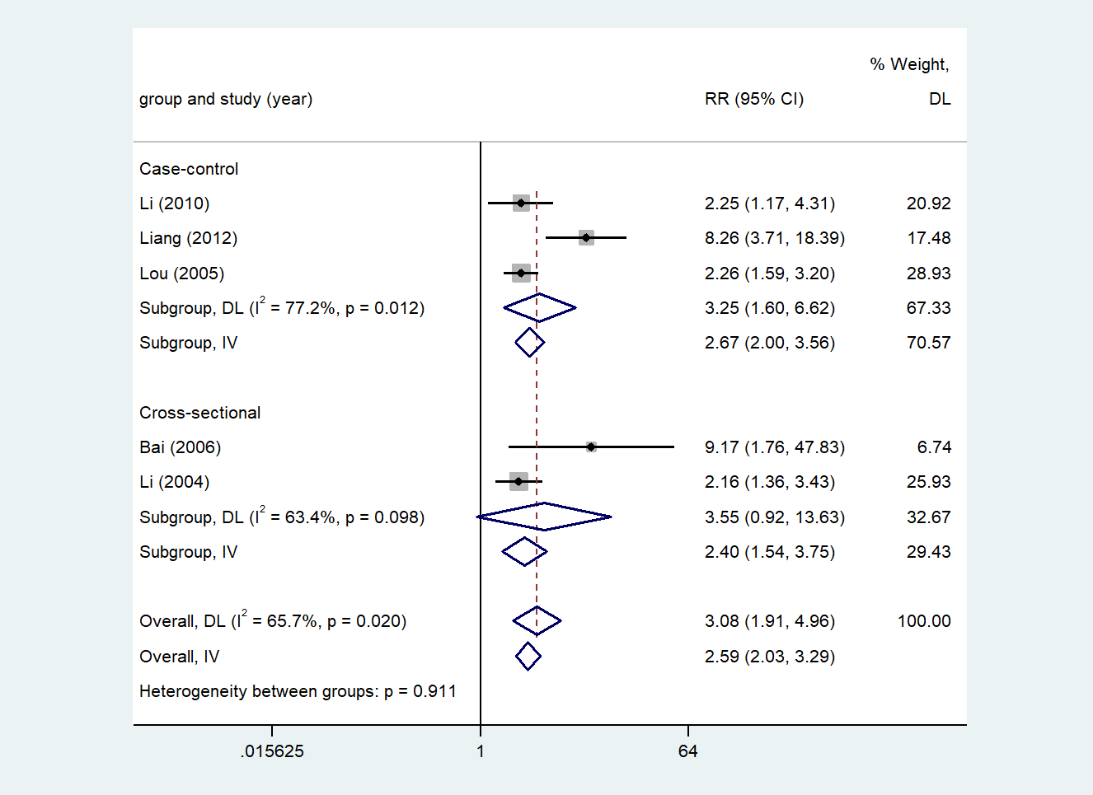


eFigure 11.2. High myopia vs no myopia on open-angle glaucoma.


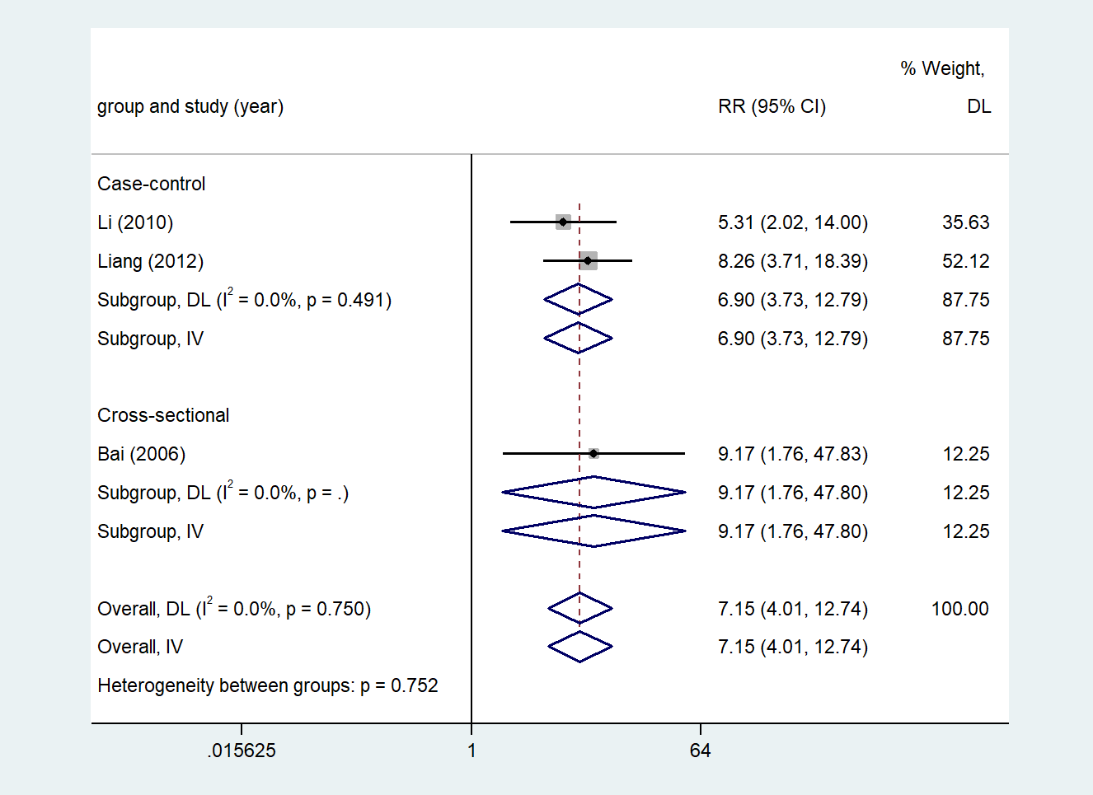


eFigure 12.1. Mild myopia vs emmetropia on diabetic retinopathy.


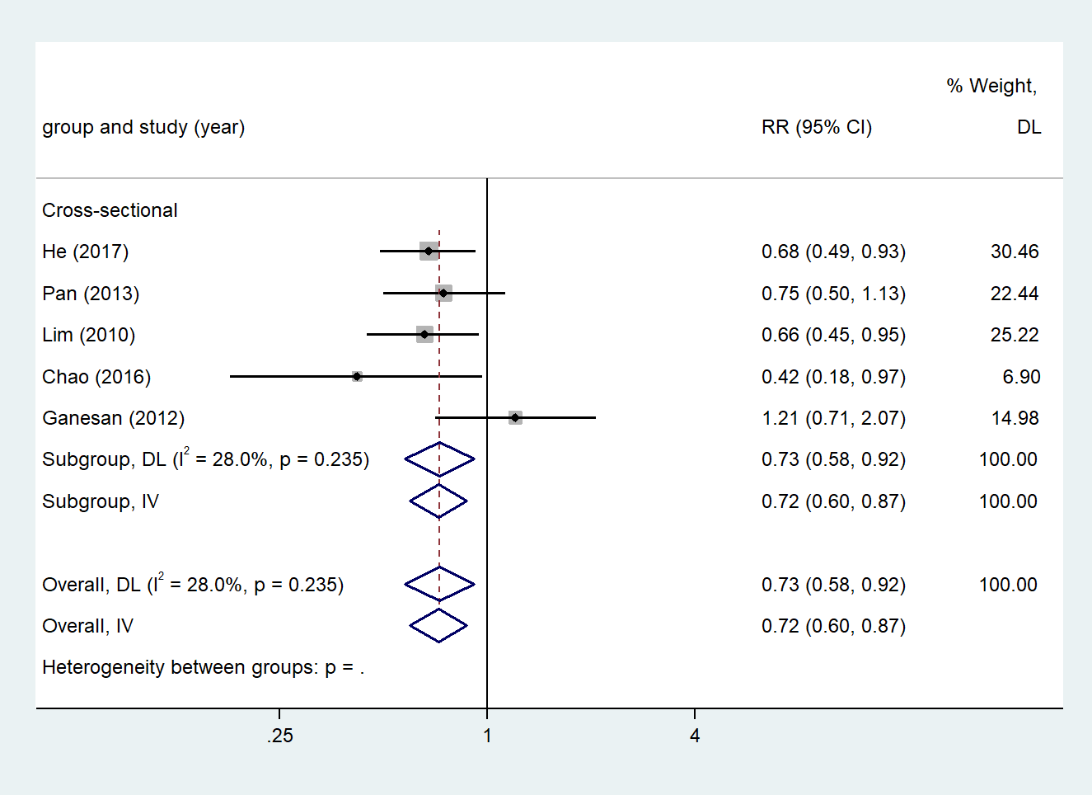


eFigure 12.2. Moderate myopia vs emmetropia on diabetic retinopathy.


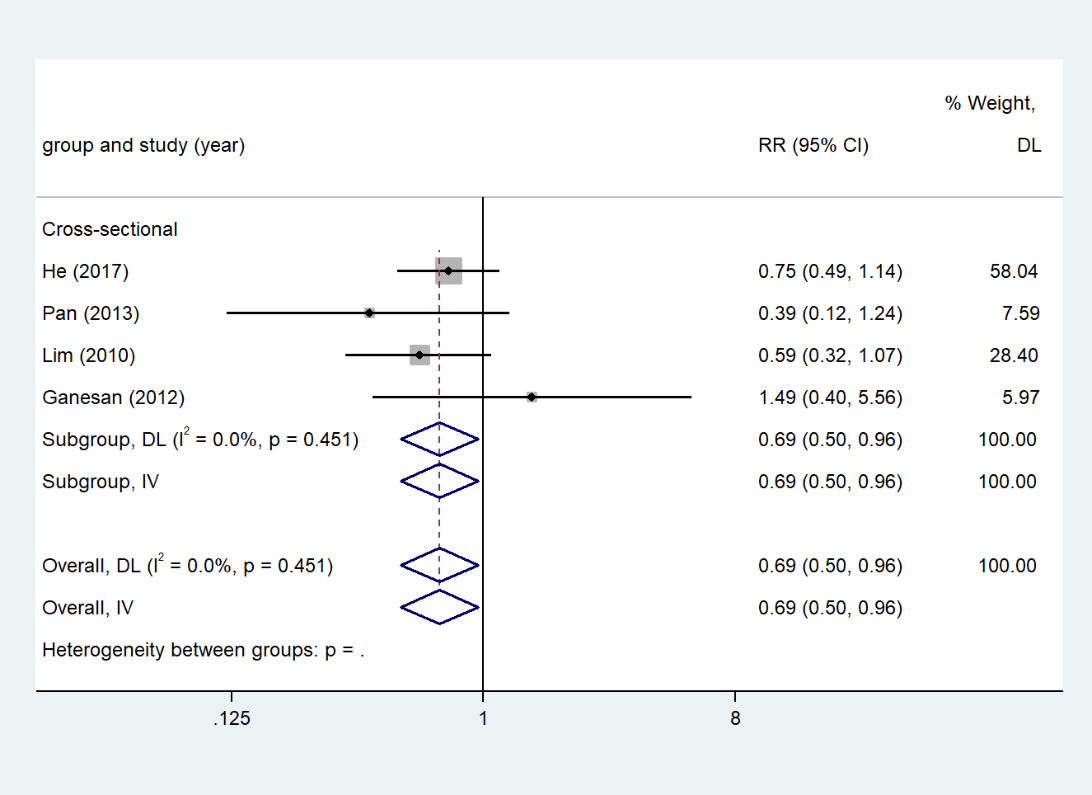


eFigure 12.3. High myopia vs emmetropia on diabetic retinopathy.


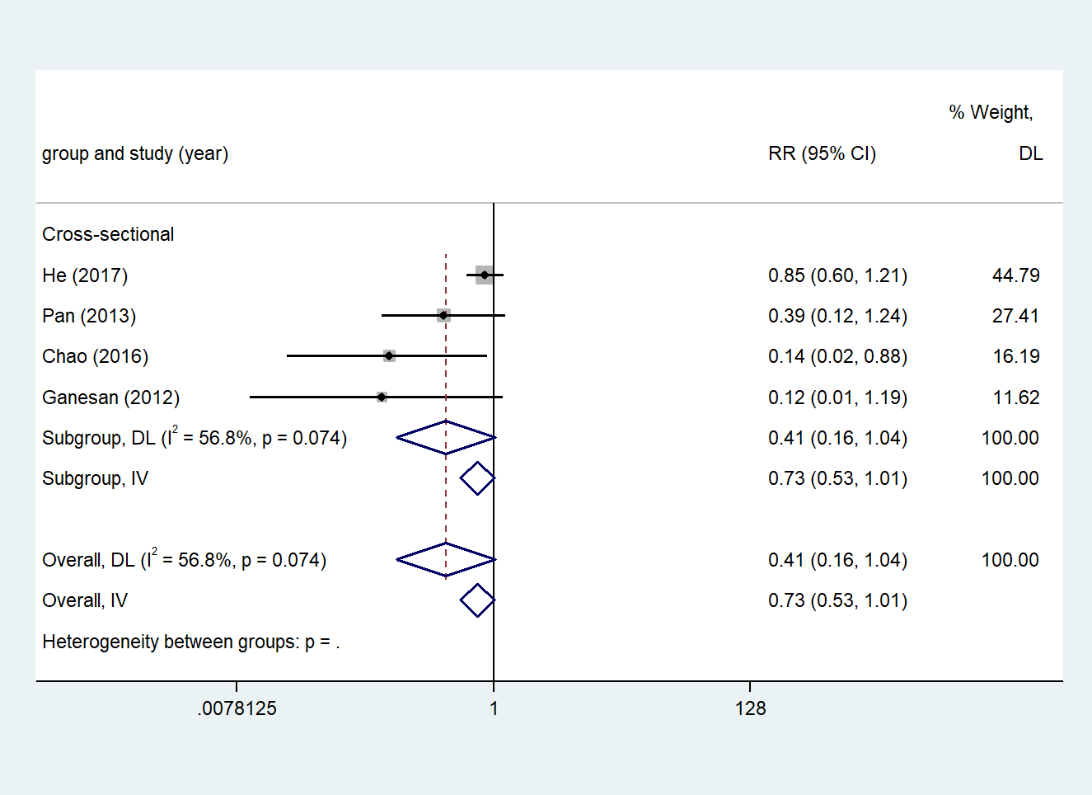


eFigure 12.4. Axial length (per millimeter increase) on diabetic retinopathy.


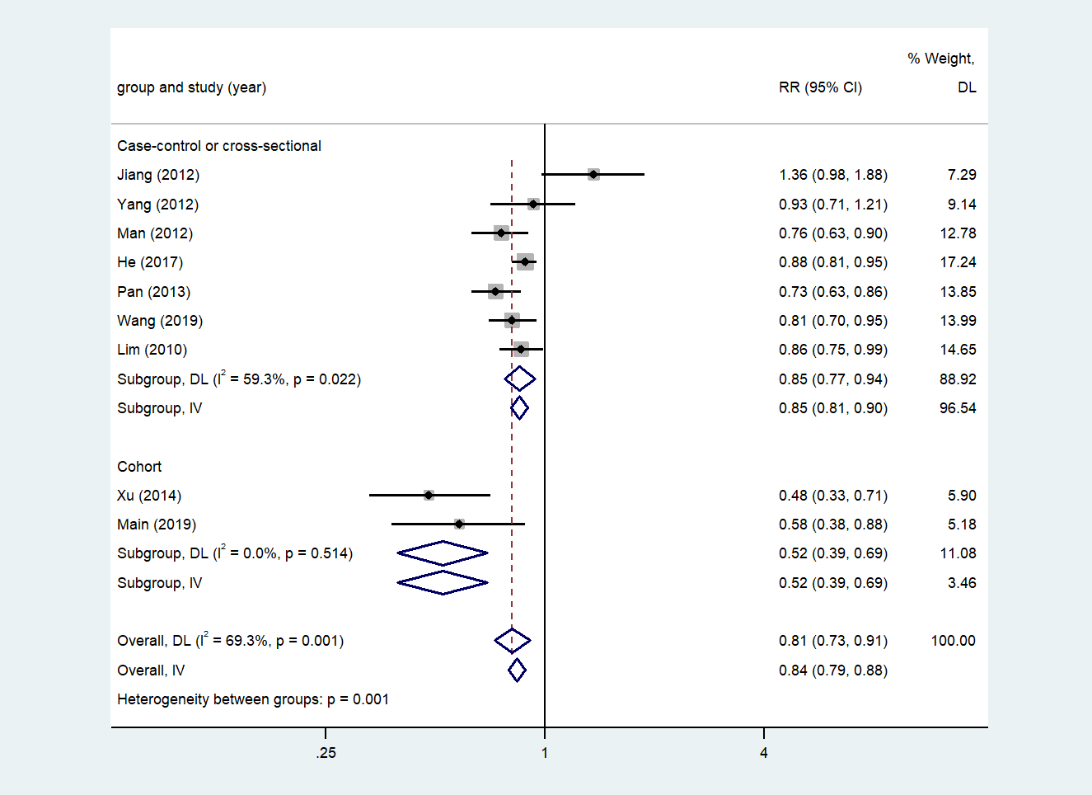


eFigure 12.5. Spherical equivalent (per diopter increase) on diabetic retinopathy.


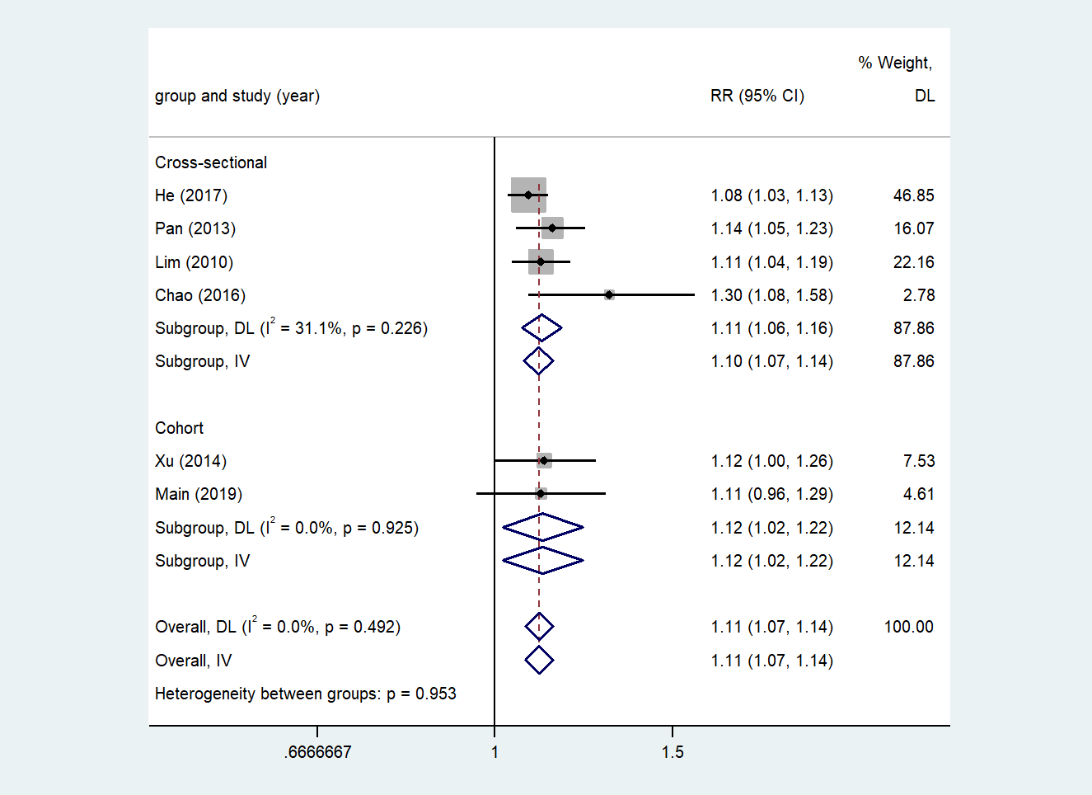


eFigure 12.6. Any myopia vs emmetropia on diabetic retinopathy progression.


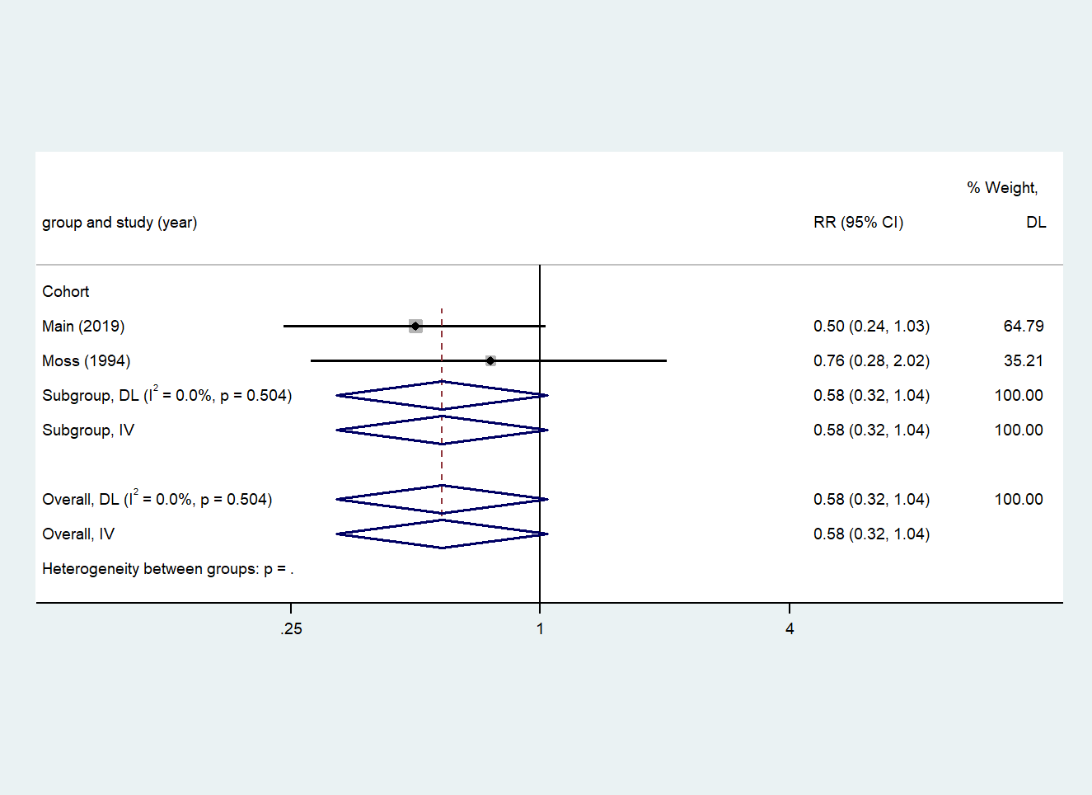


eFigure 12.7. Mild myopia vs emmetropia on vision-threatening diabetic retinopathy.


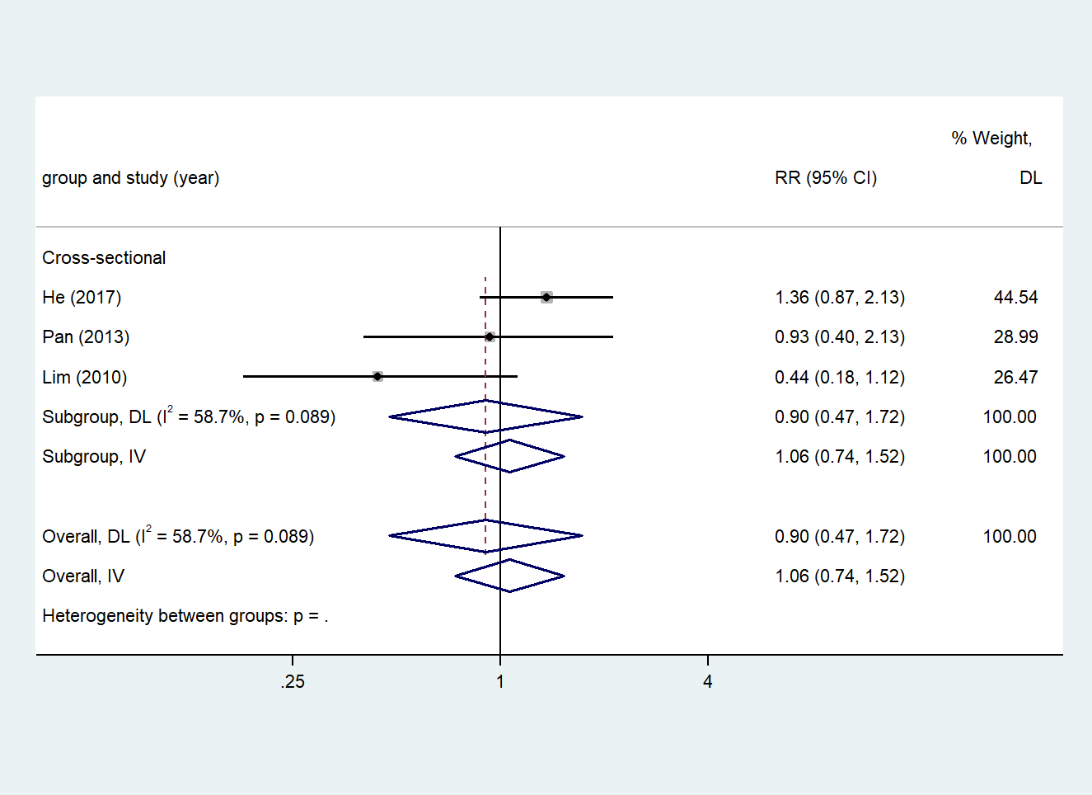


eFigure 12.8. Moderate myopia vs emmetropia on vision-threatening diabetic retinopathy.


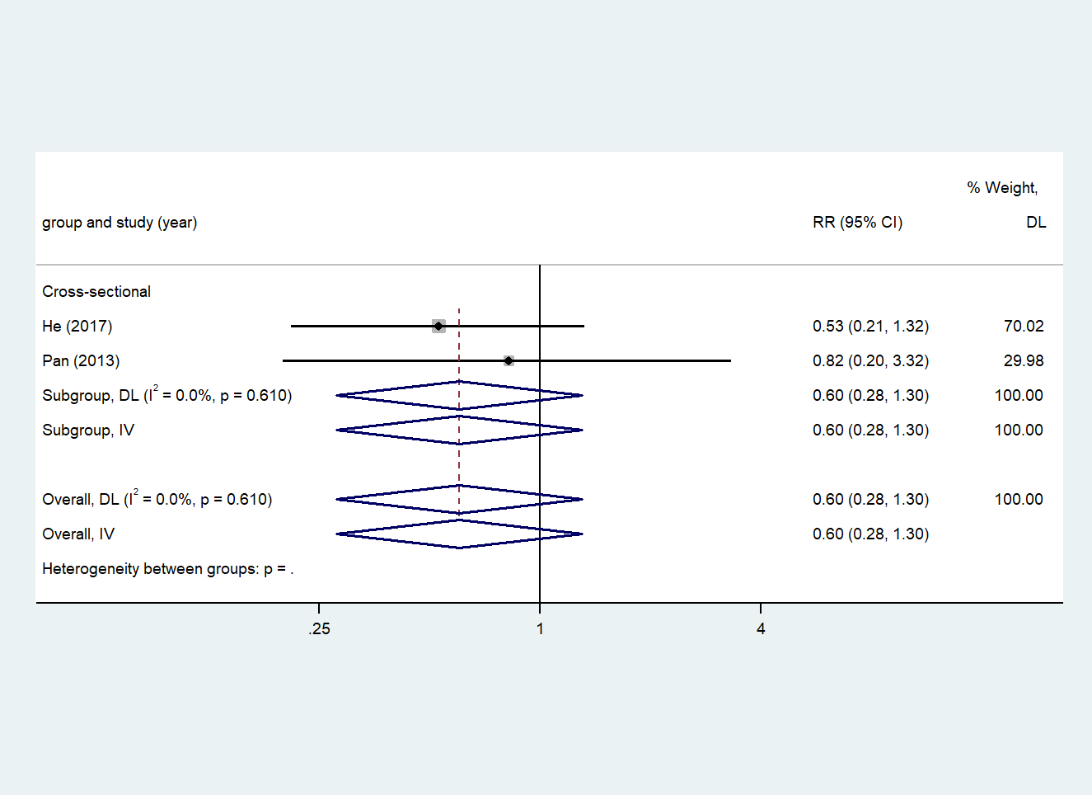


eFigure 12.9. High myopia vs emmetropia on vision-threatening diabetic retinopathy.


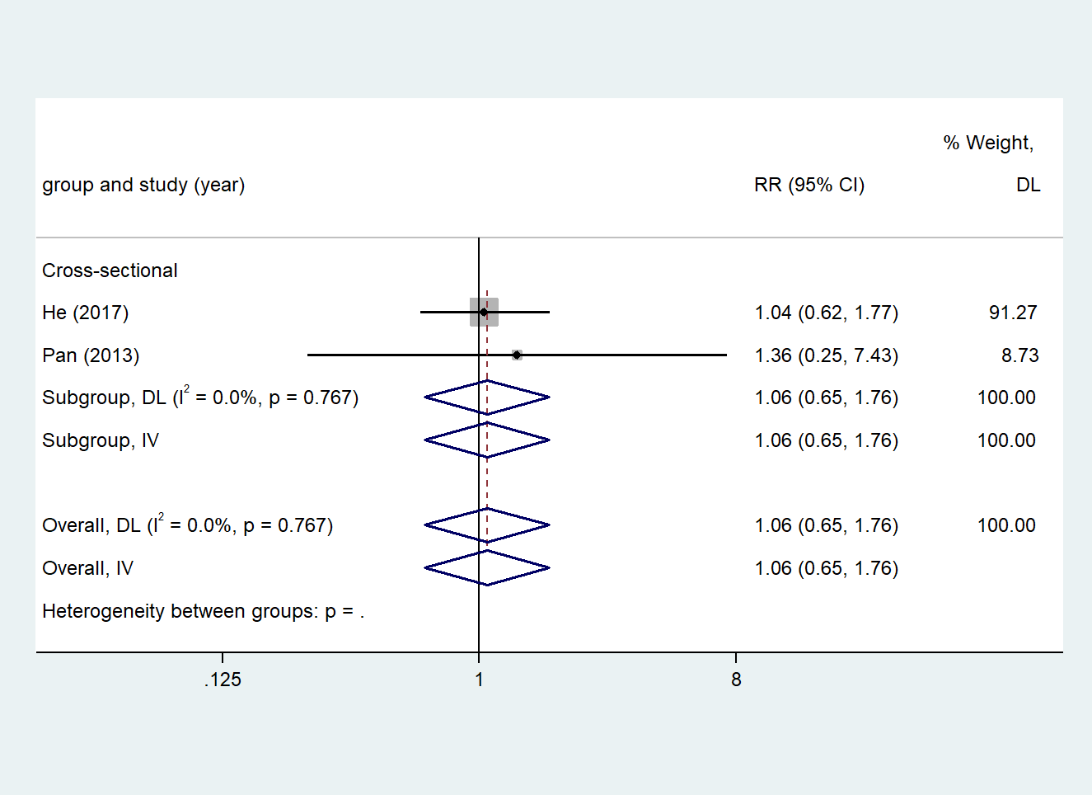


eFigure 12.10. Axial length (per millimeter increase) on vision-threatening diabetic retinopathy.


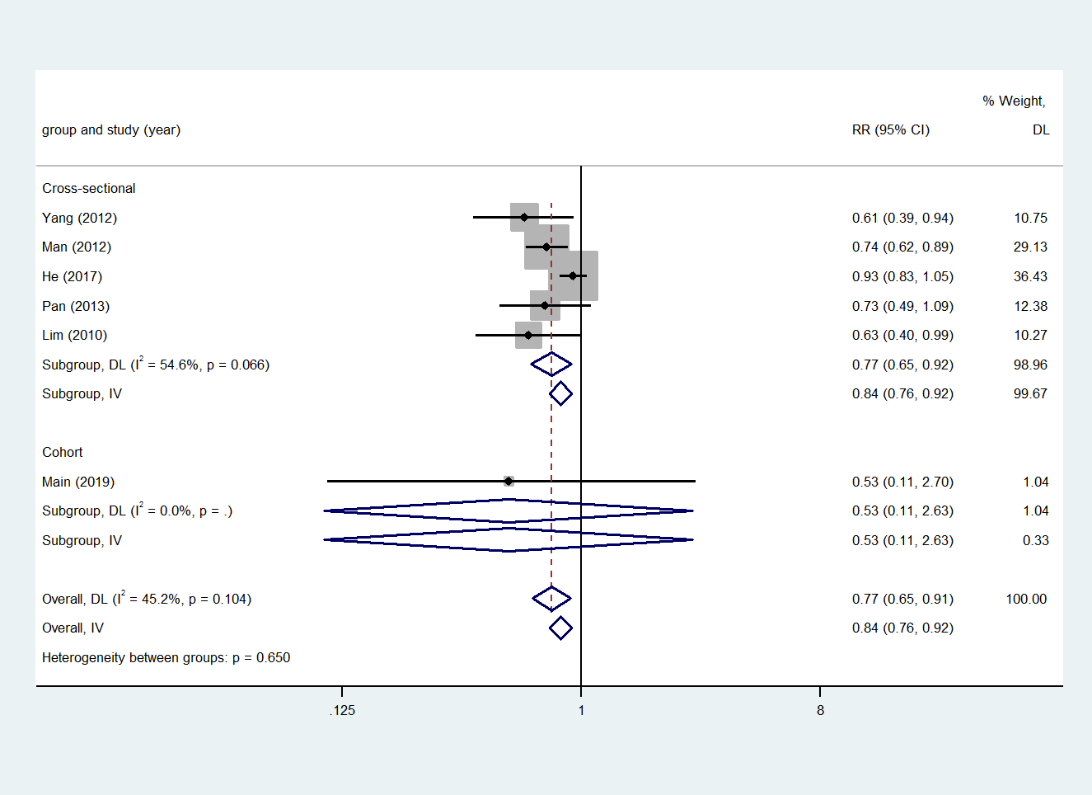


eFigure 12.11. Spherical equivalent (per diopter increase) on vision-threatening diabetic retinopathy.


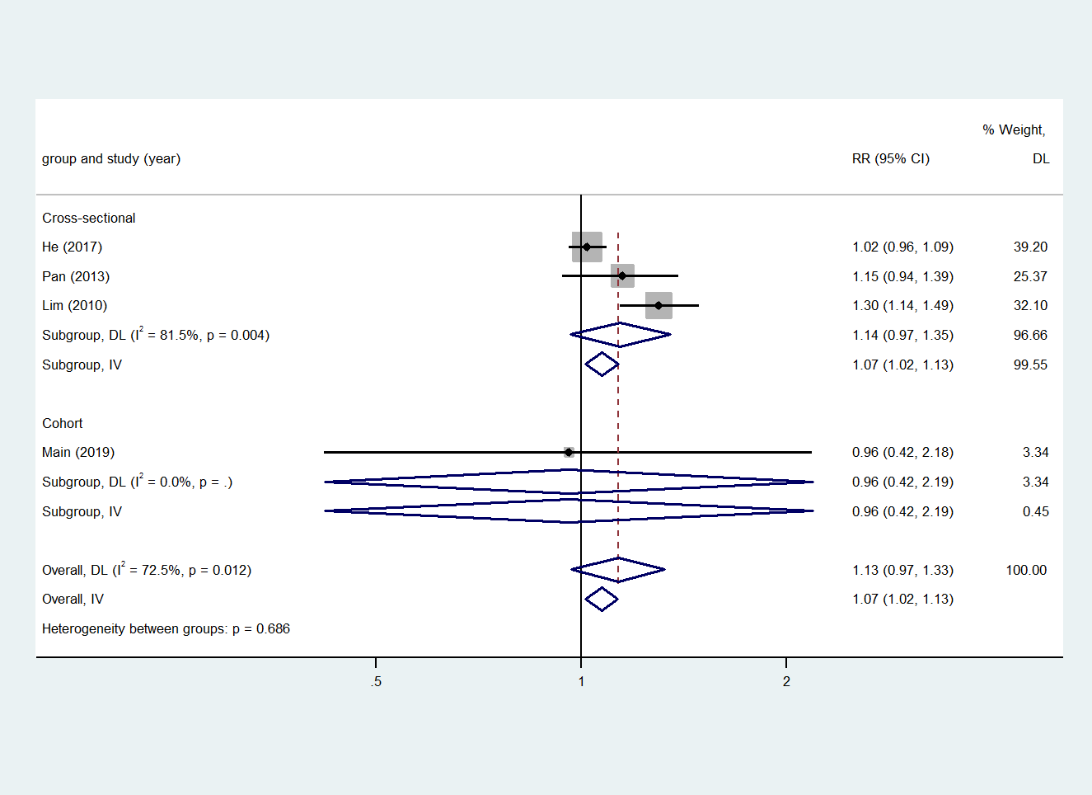

Supplement: Supplementary file 1 [file Data_Sheet_1.zip › 759767_Li_Supplementary3.docx]
