# Supplementary material for: Refractive Error and Eye Health: An Umbrella Review of Meta-Analyses
Source: Front Med (Lausanne). 2021 Nov 4;8:759767. doi: 10.3389/fmed.2021.759767 (PMC8599990; doi:10.3389/fmed.2021.759767)
Supplement: Supplementary file 1 [file Data_Sheet_1.zip › 759767_Li_Supplementary4.docx]

**Supplementary 4. GRADE classification of quality of evidence**

| **Outcome** | **Author** | **Year** | **No. of studies** | **RCTs** | **Cohort** | **Case-control** | **Cross-sectional** | **Risk of Bias** | **Inconsistency** | **Indirectness** | **Imprecision** | **Publication bias** | **Plausible confounding** | **Magnitude of effect** | **Dose-response gradient** | **Quality** |
| --- | --- | --- | --- | --- | --- | --- | --- | --- | --- | --- | --- | --- | --- | --- | --- | --- |
| **Any myopia vs non-myopia/emmetropia** | | | | | | | | | | | | | | | | |
| Myopic macular degeneration | Haarman ^13^ | 2020 | 8 | 0 | 0 | 0 | 8 | No serious risk | No serious inconsistency | No Serious Indirectness | No serious imprecision | Strongly suspected | No | No (c) | Yes | ⊕⭘⭘⭘  VERY LOW |
| Retinal detachment | Haarman ^13^ | 2020 | 5 | 0 | 0 | 5 | 0 | Serious risk | No serious inconsistency | No Serious Indirectness | No serious imprecision | Strongly suspected | No | No (c) | Yes | ⊕⭘⭘⭘  VERY LOW |
| Open angle glaucoma | Haarman ^13^ | 2020 | 14 | 0 | 0 | 0 | 14 | No serious risk | No serious inconsistency | No Serious Indirectness | No serious imprecision | Strongly suspected | No | No | Yes | ⊕⭘⭘⭘  VERY LOW |
| Nuclear cataract | Pan ^16^ | 2013 | 12 | 0 | 4 | 1 | 7 | No serious risk | Serious inconsistency | No Serious Indirectness | No serious imprecision | Undetected | No | Large | No | ⊕⊕⭘⭘  LOW |
| Cortical cataract | Pan ^16^ | 2013 | 11 | 0 | 3 | 1 | 7 | No serious risk | Serious inconsistency | No Serious Indirectness | Serious imprecision | Undetected | No | No | No | ⊕⭘⭘⭘  VERY LOW |
| PSC cataract | Pan ^16^ | 2013 | 11 | 0 | 3 | 1 | 7 | No serious risk | No serious inconsistency | No Serious Indirectness | No serious imprecision | Undetected | No | No | No | ⊕⊕⭘⭘  LOW |
| Epiretinal membrane | Xiao ^17^ | 2017 | 4 | 0 | 0 | 0 | 4 | Serious risk | Serious inconsistency (a) | No Serious Indirectness | Very serious imprecision | Undetected | No | No | No | ⊕⭘⭘⭘  VERY LOW |
| AMD | Pan ^18^ | 2013 | 9 | 0 | 3 | 0 | 6 | No serious risk | No serious inconsistency | No Serious Indirectness | No serious imprecision | Undetected | No | No | Yes | ⊕⊕⊕⭘  MODERATE |
| Early AMD | Li ^19^ | 2014 | 10 | 0 | 3 | 1 | 6 | No serious risk | No serious inconsistency | No Serious Indirectness | No serious imprecision | Detected (b) | No | No | Yes | ⊕⭘⭘⭘  VERY LOW |
| Late AMD | Li ^19^ | 2014 | 3 | 0 | 0 | 0 | 3 | No serious risk | No serious inconsistency | No Serious Indirectness | Very serious imprecision | Undetected | No | No | No | ⊕⭘⭘⭘  VERY LOW |
| Strabismus | Tang ^20^ | 2016 | 6 | 0 | 0 | 0 | 6 | Serious risk | No serious inconsistency | No Serious Indirectness | No serious imprecision | Undetected | No | Large | No | ⊕⊕⭘⭘  LOW |
| Exotropia | Tang ^20^ | 2016 | 3 | 0 | 0 | 0 | 3 | Serious risk | No serious inconsistency | No Serious Indirectness | No serious imprecision | Undetected | No | Large | No | ⊕⊕⭘⭘  LOW |
| Esotropia | Tang ^20^ | 2016 | 3 | 0 | 0 | 0 | 3 | Serious risk | No serious inconsistency | No Serious Indirectness | Serious imprecision | Undetected | No | No (c) | No | ⊕⭘⭘⭘  VERY LOW |
| Diabetic retinopathy | Wang ^15^ | 2016 | 7 | 0 | 1 | 0 | 6 | No serious risk | No serious inconsistency | No Serious Indirectness | Serious imprecision | Undetected | No | No | No | ⊕⭘⭘⭘  VERY LOW |
| Vision-threatening diabetic retinopathy | Guo ^23^ | 2015 | 6 | 0 | 1 | 1 | 4 | Serious risk | No serious inconsistency | No Serious Indirectness | Very serious imprecision | Undetected | No | No | No | ⊕⭘⭘⭘  VERY LOW |
| Diabetic retinopathy progression | He ^26^ | 2021 | 2 | 0 | 2 | 0 | 0 | Very serious risk | No serious inconsistency | No Serious Indirectness | Serious imprecision | Strongly suspected | No | No | No | ⊕⭘⭘⭘  VERY LOW |
| **Mild myopia vs non-myopia/emmetropia** | | | | | | | | | | | | | | | | |
| Myopic macular degeneration | Haarman ^13^ | 2020 | 4 | 0 | 0 | 0 | 4 | No serious risk | No serious inconsistency | No Serious Indirectness | No serious imprecision | Strongly suspected | No | No (c) | Yes | ⊕⭘⭘⭘  VERY LOW |
| Retinal detachment | Haarman ^13^ | 2020 | 3 | 0 | 0 | 3 | 0 | Serious risk | No serious inconsistency | No Serious Indirectness | No serious imprecision | Strongly suspected | No | No (c) | Yes | ⊕⭘⭘⭘  VERY LOW |
| Open angle glaucoma | Haarman ^13^ | 2020 | 10 | 0 | 0 | 0 | 10 | No serious risk | No serious inconsistency | No Serious Indirectness | No serious imprecision | Strongly suspected | No | No | Yes | ⊕⭘⭘⭘  VERY LOW |
| Nuclear cataract | Haarman ^13^ | 2020 | 7 | 0 | 2 | 0 | 5 | Serious risk | No serious inconsistency | No Serious Indirectness | No serious imprecision | Strongly suspected | No | No | Yes | ⊕⭘⭘⭘  VERY LOW |
| Cortical cataract | Haarman ^13^ | 2020 | 6 | 0 | 2 | 0 | 4 | Serious risk | No serious inconsistency | No Serious Indirectness | No serious imprecision | Strongly suspected | No | No | Yes | ⊕⭘⭘⭘  VERY LOW |
| PSC cataract | Haarman ^13^ | 2020 | 7 | 0 | 2 | 0 | 5 | Serious risk | No serious inconsistency | No Serious Indirectness | No serious imprecision | Strongly suspected | No | No | Yes | ⊕⭘⭘⭘  VERY LOW |
| Diabetic retinopathy | He ^26^ | 2021 | 5 | 0 | 0 | 0 | 5 | Very serious risk | No serious inconsistency | No Serious Indirectness | No serious imprecision | Strongly suspected | Yes | No | Yes | ⊕⭘⭘⭘  VERY LOW |
| Vision-threatening diabetic retinopathy | He ^26^ | 2021 | 3 | 0 | 0 | 0 | 3 | Very serious risk | Serious inconsistency | No Serious Indirectness | Very serious imprecision | Strongly suspected | No | No | No | ⊕⭘⭘⭘  VERY LOW |
| **Moderate myopia vs non-myopia/emmetropia** | | | | | | | | | | | | | | | | |
| Myopic macular degeneration | Haarman ^13^ | 2020 | 6 | 0 | 0 | 0 | 6 | No serious risk | No serious inconsistency | No Serious Indirectness | No serious imprecision | Strongly suspected | No | No (c) | Yes | ⊕⭘⭘⭘  VERY LOW |
| Retinal detachment | Haarman ^13^ | 2020 | 2 | 0 | 0 | 2 | 0 | No serious risk | No serious inconsistency | No Serious Indirectness | No serious imprecision | Strongly suspected | No | No (c) | Yes | ⊕⭘⭘⭘  VERY LOW |
| Nuclear cataract | Haarman ^13^ | 2020 | 6 | 0 | 1 | 0 | 5 | Serious risk | No serious inconsistency | No Serious Indirectness | No serious imprecision | Strongly suspected | No | No (c) | Yes | ⊕⭘⭘⭘  VERY LOW |
| Cortical cataract | Haarman ^13^ | 2020 | 5 | 0 | 1 | 0 | 4 | Serious risk | No serious inconsistency | No Serious Indirectness | Serious imprecision | Strongly suspected | No | No | Yes | ⊕⭘⭘⭘  VERY LOW |
| PSC cataract | Haarman ^13^ | 2020 | 6 | 0 | 1 | 0 | 5 | Serious risk | No serious inconsistency | No Serious Indirectness | No serious imprecision | Strongly suspected | No | No (c) | Yes | ⊕⭘⭘⭘  VERY LOW |
| Diabetic retinopathy | He ^26^ | 2021 | 4 | 0 | 0 | 0 | 4 | Very serious risk | No serious inconsistency | No Serious Indirectness | No serious imprecision | Strongly suspected | Yes | No | Yes | ⊕⭘⭘⭘  VERY LOW |
| Vision-threatening diabetic retinopathy | He ^26^ | 2021 | 2 | 0 | 0 | 0 | 2 | Very serious risk | No serious inconsistency | No Serious Indirectness | Very serious imprecision | Strongly suspected | No | No | No | ⊕⭘⭘⭘  VERY LOW |
| **High myopia vs non-myopia/emmetropia** | | | | | | | | | | | | | | | | |
| Myopic macular degeneration | Haarman ^13^ | 2020 | 6 | 0 | 0 | 0 | 6 | No serious risk | No serious inconsistency | No Serious Indirectness | No serious imprecision | Strongly suspected | No | No (c) | Yes | ⊕⭘⭘⭘  VERY LOW |
| Retinal detachment | Haarman ^13^ | 2020 | 3 | 0 | 0 | 3 | 0 | No serious risk | No serious inconsistency | No Serious Indirectness | No serious imprecision | Strongly suspected | No | No (c) | Yes | ⊕⭘⭘⭘  VERY LOW |
| Nuclear cataract | Haarman ^13^ | 2020 | 7 | 0 | 2 | 0 | 5 | Serious risk | No serious inconsistency | No Serious Indirectness | No serious imprecision | Strongly suspected | No | No (c) | Yes | ⊕⭘⭘⭘  VERY LOW |
| Cortical cataract | Haarman ^13^ | 2020 | 6 | 0 | 2 | 0 | 4 | Serious risk | No serious inconsistency | No Serious Indirectness | Serious imprecision | Strongly suspected | No | No | Yes | ⊕⭘⭘⭘  VERY LOW |
| PSC cataract | Haarman ^13^ | 2020 | 7 | 0 | 2 | 0 | 5 | Serious risk | No serious inconsistency | No Serious Indirectness | No serious imprecision | Strongly suspected | No | No (c) | Yes | ⊕⭘⭘⭘  VERY LOW |
| Diabetic retinopathy | He ^26^ | 2021 | 4 | 0 | 0 | 0 | 4 | Very serious risk | No serious inconsistency | No Serious Indirectness | Serious imprecision | Strongly suspected | No | No (c) | Yes | ⊕⭘⭘⭘  VERY LOW |
| Vision-threatening diabetic retinopathy | He ^26^ | 2021 | 2 | 0 | 0 | 0 | 2 | Very serious risk | No serious inconsistency | No Serious Indirectness | Very serious imprecision | Strongly suspected | No | No | No | ⊕⭘⭘⭘  VERY LOW |
| Open angle glaucoma | Xiang ^25^ | 2014 | 3 | 0 | 0 | 2 | 1 | Serious risk | No serious inconsistency | No Serious Indirectness | No serious imprecision | Strongly suspected | No | No (c) | No | ⊕⭘⭘⭘  VERY LOW |
| **High myopia vs no high myopia** | | | | | | | | | | | | | | | | |
| Re-RD after SOR | He ^22^ | 2018 | 6 | 1 | 0 | 5 | 0 | No serious risk | No serious inconsistency | No Serious Indirectness | No serious imprecision | Strongly suspected | No | Large | No | ⊕⊕⭘⭘  LOW |
| Sub-foveal choroidal thickness | Wang ^27^ | 2015 | 8 | NP | NP | NP | NP | No serious risk | Serious inconsistency (a) | No Serious Indirectness | N.A. | Undetected | Yes | N.A. | No | ⊕⊕⭘⭘  LOW |
| **Mild/moderate myopia vs non-myopia** | | | | | | | | | | | | | | | | |
| Diabetic retinopathy | Guo ^23^ | 2015 | 3 | 0 | 0 | 0 | 3 | Serious risk | No serious inconsistency | No Serious Indirectness | Serious imprecision | Undetected | No | No | No | ⊕⭘⭘⭘  VERY LOW |
| **Moderate/high myopia vs non-myopia** | | | | | | | | | | | | | | | | |
| Open angle glaucoma | Haarman ^13^ | 2020 | 10 | 0 | 0 | 0 | 10 | No serious risk | No serious inconsistency | No Serious Indirectness | No serious imprecision | Strongly suspected | No | No (c) | Yes | ⊕⭘⭘⭘  VERY LOW |
| **Hyperopia vs emmetropia** | | | | | | | | | | | | | | | | |
| AMD | Pan ^18^ | 2013 | 9 | 0 | 3 | 0 | 6 | No serious risk | No serious inconsistency | No Serious Indirectness | No serious imprecision | Undetected | No | No | Yes | ⊕⊕⊕⭘  MODERATE |
| Early AMD | Li ^19^ | 2014 | 13 | 0 | 5 | 2 | 6 | Serious risk | No serious inconsistency | No Serious Indirectness | No serious imprecision | Undetected | No | No | Yes | ⊕⊕⭘⭘  LOW |
| Late AMD | Li ^19^ | 2014 | 7 | 0 | 3 | 0 | 4 | Serious risk | No serious inconsistency | No Serious Indirectness | No serious imprecision | Undetected | Yes | No | No | ⊕⊕⭘⭘  LOW |
| Strabismus | Tang ^20^ | 2016 | 6 | 0 | 0 | 0 | 6 | Serious risk | Serious inconsistency | No Serious Indirectness | No serious imprecision | Undetected | No | Large | No | ⊕⭘⭘⭘  VERY LOW |
| Exotropia | Tang ^20^ | 2016 | 3 | 0 | 0 | 0 | 3 | Serious risk | Serious inconsistency | No Serious Indirectness | Very serious imprecision | Undetected | No | No (c) | No | ⊕⭘⭘⭘  VERY LOW |
| Esotropia | Tang ^20^ | 2016 | 3 | 0 | 0 | 0 | 3 | Serious risk | No serious inconsistency | No Serious Indirectness | No serious imprecision | Undetected | No | Large | Yes | ⊕⊕⊕⭘  MODERATE |
| Diabetic retinopathy | Guo ^23^ | 2015 | 4 | 0 | 0 | 0 | 4 | Serious risk | No serious inconsistency | No Serious Indirectness | No serious imprecision | Undetected | No | No | No | ⊕⭘⭘⭘  VERY LOW |
| Epiretinal membrane | Xiao ^17^ | 2017 | 3 | 0 | 0 | 0 | 3 | Serious risk | Serious inconsistency (a) | No Serious Indirectness | No serious imprecision | Undetected | No | No | No | ⊕⭘⭘⭘  VERY LOW |
| **Astigmatism vs emmetropia** | | | | | | | | | | | | | | | | |
| Strabismus | Tang ^20^ | 2016 | 6 | 0 | 0 | 0 | 6 | Serious risk | No serious inconsistency | No Serious Indirectness | No serious imprecision | Undetected | No | Large | No | ⊕⊕⭘⭘  LOW |
| Exotropia | Tang ^20^ | 2016 | 3 | 0 | 0 | 0 | 3 | Serious risk | No serious inconsistency | No Serious Indirectness | No serious imprecision | Undetected | No | Large | No | ⊕⊕⭘⭘  LOW |
| Esotropia | Tang ^20^ | 2016 | 3 | 0 | 0 | 0 | 3 | Serious risk | No serious inconsistency | No Serious Indirectness | No serious imprecision | Undetected | No | Large | No | ⊕⊕⭘⭘  LOW |
| **Anisometropia vs emmetropia** | | | | | | | | | | | | | | | | |
| Strabismus | Tang ^20^ | 2016 | 6 | 0 | 0 | 0 | 6 | Serious risk | No serious inconsistency | No Serious Indirectness | No serious imprecision | Undetected | No | Large | No | ⊕⊕⭘⭘  LOW |
| Exotropia | Tang ^20^ | 2016 | 4 | 0 | 0 | 0 | 4 | Serious risk | No serious inconsistency | No Serious Indirectness | No serious imprecision | Undetected | No | Large | No | ⊕⊕⭘⭘  LOW |
| Esotropia | Tang ^20^ | 2016 | 4 | 0 | 0 | 0 | 4 | Serious risk | No serious inconsistency | No Serious Indirectness | No serious imprecision | Undetected | No | Large | No | ⊕⊕⭘⭘  LOW |
| **Axial length (per millimeter increase)** | | | | | | | | | | | | | | | | |
| AMD | Pan ^18^ | 2013 | 3 | 0 | 0 | 0 | 3 | No serious risk | No serious inconsistency | No Serious Indirectness | No serious imprecision | Undetected | No | No | Yes | ⊕⊕⊕⭘  MODERATE |
| Early AMD | Li ^19^ | 2014 | 4 | 0 | 0 | 0 | 4 | No serious risk | No serious inconsistency | No Serious Indirectness | No serious imprecision | Undetected | No | No | Yes | ⊕⊕⊕⭘  MODERATE |
| Late AMD | Li ^19^ | 2014 | 2 | 0 | 0 | 0 | 2 | No serious risk | No serious inconsistency | No Serious Indirectness | Very serious imprecision | Undetected | No | No | No | ⊕⭘⭘⭘  VERY LOW |
| Diabetic retinopathy | He ^26^ | 2021 | 9 | 0 | 2 | 1 | 6 | No serious risk | Serious inconsistency | No Serious Indirectness | No serious imprecision | Strongly suspected | Yes | No | Yes | ⊕⭘⭘⭘  VERY LOW |
| Vision-threatening diabetic retinopathy | He ^26^ | 2021 | 6 | 0 | 1 | 0 | 5 | Very serious risk | No serious inconsistency | No Serious Indirectness | No serious imprecision | Strongly suspected | Yes | No | Yes | ⊕⭘⭘⭘  VERY LOW |
| **Spherical equivalent (per D increase)** | | | | | | | | | | | | | | | | |
| AMD | Pan ^18^ | 2013 | 7 | 0 | 2 | 0 | 5 | No serious risk | No serious inconsistency | No Serious Indirectness | No serious imprecision | Undetected | No | No | Yes | ⊕⊕⊕⭘  MODERATE |
| Early AMD | Li ^19^ | 2014 | 7 | 0 | 2 | 0 | 5 | No serious risk | No serious inconsistency | No Serious Indirectness | No serious imprecision | Undetected | No | No | Yes | ⊕⊕⊕⭘  MODERATE |
| Late AMD | Li ^19^ | 2014 | 4 | 0 | 1 | 0 | 3 | No serious risk | No serious inconsistency | No Serious Indirectness | No serious imprecision | Undetected | No | No | No | ⊕⊕⭘⭘  LOW |
| Diabetic retinopathy | He ^26^ | 2021 | 6 | 0 | 2 | 0 | 4 | Very serious risk | No serious inconsistency | No Serious Indirectness | No serious imprecision | Strongly suspected | No | No | Yes | ⊕⭘⭘⭘  VERY LOW |
| Vision-threatening diabetic retinopathy | He ^26^ | 2021 | 4 | 0 | 1 | 0 | 3 | Very serious risk | Serious inconsistency | No Serious Indirectness | Serious imprecision | Strongly suspected | No | No | No | ⊕⭘⭘⭘  VERY LOW |

1. Based on heterogeneity (I^2^＞50%)
2. Egger’s test *P* value <0.05
3. Based on low methodological quality and serious imprecision

NP=not published; N.A.=not applicable
