# Supplementary material for: Refractive Error and Eye Health: An Umbrella Review of Meta-Analyses
Source: Front Med (Lausanne). 2021 Nov 4;8:759767. doi: 10.3389/fmed.2021.759767 (PMC8599990; doi:10.3389/fmed.2021.759767)
Supplement: Supplementary file 1 [file Data_Sheet_1.zip › 759767_Li_Supplementary5.docx]

**Supplementary 5. The overlaps among the systematical reviews and meta-analyses**

**Supplementary 5.1. Any myopia vs non-myopia/emmetropia**

***Supplementary 5.1.1. Nuclear cataract***

|  | Haarman ^13^ | Pan ^16^ |
| --- | --- | --- |
| Wu et al. (1999) | ✓ | No |
| Lim et al. (1999) | ✓ | ✓ |
| Wong et al. (2001) | ✓ ^(a)^ | ✓ ^(b)^ |
| Wong et al. (2003) | ✓ | ✓ |
| Chang et al. (2005) | ✓ | ✓ |
| Giuffre et al. (2005) | ✓ | ✓ |
| Mukesh et al. (2006) | ✓ | ✓ |
| Pan et al. (2013) * | ✓ | ✓ |
| Pan et al. (2013) ** | ✓ | ✓ |
| Duan et al. (2013) | ✓ | ✓ |
| Kanthan et al. (2014) | ✓ | No |
| Younan et al. (2002) | No | ✓ |
| Leske et al. (2002) | No | ✓ |

***Supplementary 5.1.2. Cortical cataract***

|  | Haarman ^13^ | Pan ^16^ |
| --- | --- | --- |
| Wu et al. (1999) | ✓ | No |
| Lim et al. (1999) | ✓ | ✓ |
| Wong et al. (2001) | ✓^(a)^ | ✓^(b)^ |
| Wong et al. (2003) | ✓ | ✓ |
| Chang et al. (2005) | ✓ | ✓ |
| Giuffre et al. (2005) | ✓ | ✓ |
| Mukesh et al. (2006) | ✓ | ✓ |
| Pan et al. (2013)* | ✓ | ✓ |
| Pan et al. (2013)** | ✓ | ✓ |
| Duan et al. (2013) | ✓ | ✓ |
| Kanthan et al. (2014) | ✓ | No |
| Younan et al. (2002) | No | ✓ |
| Leske et al. (2002) | No | No |

***Supplementary 5.1.3. Posterior subcapsular cataract***

|  | Haarman ^13^ | Pan ^16^ |
| --- | --- | --- |
| Wu et al. (1999) | ✓ | No |
| Lim et al. (1999) | ✓ | ✓ |
| Wong et al. (2001) | ✓^(a)^ | ✓^(b)^ |
| Wong et al. (2003) | ✓ | ✓ |
| Chang et al. (2005) | ✓ | ✓ |
| Giuffre et al. (2005) | ✓ | ✓ |
| Mukesh et al. (2006) | ✓ | ✓ |
| Pan et al. (2013)* | ✓ | ✓ |
| Pan et al. (2013)** | ✓ | ✓ |
| Duan et al. (2013) | ✓ | ✓ |
| Kanthan et al. (2014) | ✓ | No |
| Younan et al. (2002) | No | ✓ |
| Leske et al. (2002) | No | No |

***Supplementary 5.1.4. Open angle glaucoma***

|  | Haarman ^13^ | Marcus ^21^ | Xiong ^24^ | Xiang ^25^ |
| --- | --- | --- | --- | --- |
| Wu et al. (1999) | ✓ | ✓ | No | No |
| Mitchell et al. (1999) | ✓ | ✓ | No | No |
| Weih et al. (2001) | ✓ | ✓ | ✓ | No |
| Wong et al. (2003) | ✓ | ✓ | ✓ | No |
| Ramakrishnan et al. (2003) | ✓ | ✓ | ✓ | No |
| Suzuki et al. (2006) | ✓ | ✓ | ✓ | No |
| Xu et al. (2007) | ✓ | ✓ | ✓ | No |
| Casson et al. (2007) | ✓ | ✓ | ✓ | No |
| Garudadru et al. (2010) | ✓ | ✓ | ✓ | No |
| Perera et al. (2010) | ✓ | ✓ | ✓ | No |
| Kuzin et al. (2010) | ✓ | ✓ | ✓ | No |
| Qiu et al. (2013) | ✓ | No | ✓ | No |
| Pan et al. (2013) | ✓ | No | No | No |
| Chon et al. (2013) | ✓ | No | No | No |
| Ponte et al. (1994) | No | ✓ | No | No |
| Czudowska et al. (2010) | No | ✓ | No | No |
| Li et al. (2004) | No | No | ✓ | ✓ |
| Bai et al. (2006) | No | No | No | ✓ |
| Liang et al. (2012) | No | No | No | ✓ |
| Li et al. (2010) | No | No | No | ✓ |
| Lou et al. (2005) | No | No | No | ✓ |

***Supplementary 5.1.5. Diabetic retinopathy***

|  | Fu ^14^ | Wang ^15^ | Guo ^23^ |
| --- | --- | --- | --- |
| Lim et al. (2010) | ✓ | ✓ | ✓ |
| Man et al. (2012) | ✓ | ✓ | ✓ |
| Pan et al. (2013) | ✓ | ✓ | ✓ |
| Rand et al. (1985) | ✓ | ✓ | No |
| Baker et al. (1986) | ✓ | No | No |
| Moss et al. (1994) | ✓ | ✓ | ✓ |
| Ganesan et al. (2012) | No | ✓ | ✓ |
| Jee et al. (2013) | No | ✓ | ✓ |

**Supplementary 5.2. Mild myopia vs non-myopia**

***Open angle glaucoma***

|  | Haarman ^13^ | Marcus ^21^ | Xiong ^24^ |
| --- | --- | --- | --- |
| Mitchell et al. (1999) | ✓ | ✓ | No |
| Wong et al. (2003) | ✓ | ✓ | ✓ |
| Ramakrishnan et al. (2003) | ✓ | ✓ | ✓ |
| Suzuki et al. (2006) | ✓ | ✓ | ✓ |
| Xu et al. (2007) | ✓ | ✓ | ✓ |
| Perera et al. (2010) | ✓ | ✓ | ✓ |
| Kuzin et al. (2010) | ✓ | ✓ | ✓ |
| Qiu et al. (2013) | ✓ | No | ✓ |
| Pan et al. (2013) | ✓ | No | No |
| Chon et al. (2013) | ✓ | No | No |
| Czudowska et al. (2010) | No | ✓ | No |
| Li et al. (2004) | No | No | ✓ |

**Supplementary 5.3. High myopia vs non-myopia/emmetropia**

***Diabetic retinopathy***

|  | Guo ^23^ | He ^26^ |
| --- | --- | --- |
| Lim et al. (2010) | ✓ | No |
| Pan et al. (2013) | ✓ | ✓ |
| Ganesan et al. (2012) | ✓ | ✓ |
| He et al. (2017) | No | ✓ |
| Chao et al. (2016) | No | ✓ |

**Supplementary 5.4. Moderate/high myopia vs non-myopia**

***Open angle glaucoma***

|  | Haarman ^13^ | Marcus ^21^ | Xiong ^24^ |
| --- | --- | --- | --- |
| Mitchell et al. (1999) | ✓ | ✓ | No |
| Wong et al. (2003) | ✓ | ✓ | ✓ |
| Ramakrishnan et al. (2003) | ✓ | ✓ | ✓ |
| Suzuki et al. (2006) | ✓ | ✓ | ✓ |
| Xu et al. (2007) | ✓ | ✓ | ✓ |
| Perera et al. (2010) | ✓ | ✓ | ✓ |
| Kuzin et al. (2010) | ✓ | ✓ | ✓ |
| Qiu et al. (2013) | ✓ | No | ✓ |
| Pan et al. (2013) | ✓ | No | No |
| Chon et al. (2013) | ✓ | No | No |
| Czudowska et al. (2010) | No | ✓ | No |
| Li et al. (2004) | No | No | ✓ |

**Supplementary 5.5. AL (per millimeter increase)**

***Supplementary 5.5.1. Diabetic retinopathy***

|  | Fu ^14^ | Wang ^15^ | He ^26^ |
| --- | --- | --- | --- |
| Lim et al. (2010) | ✓ | ✓ | ✓ |
| Yang et al. (2012) | ✓ | ✓ | ✓ |
| Man et al. (2012) | ✓ | ✓ | ✓ |
| Pan et al. (2013) | ✓ | ✓ | ✓ |
| Man et al. (2014) | ✓ | No | No |
| Xu et al. (2014) | ✓ | No | ✓ |
| Ganesan et al. (2012) | No | ✓ | No |
| Jiang et al. (2012) | No | No | ✓ |
| Man et al. (2019) | No | No | ✓ |
| He et al. (2017) | No | No | ✓ |
| Wang et al. (2019) | No | No | ✓ |

***Supplementary 5.5.2. Vision-threatening diabetic retinopathy***

|  | Wang ^15^ | He ^26^ |
| --- | --- | --- |
| Lim et al. (2010) | ✓ | ✓ |
| Pan et al. (2013) | ✓ | ✓ |
| Ganesan et al. (2012) | ✓ | No |
| Man et al. (2012) | ✓ | ✓ |
| Yang et al. (2012) | ✓ | ✓ |
| Man et al. (2019) | No | ✓ |
| He et al. (2017) | No | ✓ |

**Supplementary 5.6. Spherical equivalence (per diopter change)**

***Diabetic retinopathy***

|  | Fu ^14^ | He ^26^ |
| --- | --- | --- |
| Lim et al. (2010) | ✓ | ✓ |
| Pan et al. (2013) | ✓ | ✓ |
| Xie et al. (2008) | ✓ | No |
| Jiang et al. (2012) | ✓ | No |
| Xu et al. (2014) | ✓ | ✓ |
| Man et al. (2019) | No | ✓ |
| He et al. (2017) | No | ✓ |
| Chao et al. (2016) | No | ✓ |

1. Only including cohort study
2. Including cross-sectional study and cohort study separately
