# Supplementary material for: Refractive Error and Eye Health: An Umbrella Review of Meta-Analyses
Source: Front Med (Lausanne). 2021 Nov 4;8:759767. doi: 10.3389/fmed.2021.759767 (PMC8599990; doi:10.3389/fmed.2021.759767)
Supplement: Supplementary file 1 [file Data_Sheet_1.zip › 759767_Li_Supplementary6.docx]

**Supplementary 6. Quality assessment of original studies from published systematical reviews and meta-analyses**

**Supplementary 6.1.**

| Haarman 2020 ^13^ | |
| --- | --- |
| Quality assessment tool | Sanderson et al ^(1)^ |
| The details of the quality assessment were not shown | |

**Supplementary 6.2.**

| Fu 2016 ^14^ | |
| --- | --- |
| Quality assessment tool | Newcastle-Ottawa Scale |
| Study | NOS Scores |
| Rand (1985) | 6 |
| Baker (1986) | 7 |
| Moss (1994) | 7 |
| Xie (2008) | 7 |
| Lim (2010) | 8 |
| Yang (2012) | 7 |
| Man (2012) | 8 |
| Jiang (2012) | 6 |
| Pan (2013) | 8 |
| Man (2014) | 7 |
| Xu (2014) | 8 |

**Supplementary 6.3.**

| Wang 2016 ^15^ | |
| --- | --- |
| Quality assessment tool | Sanderson et al ^(1)^ |
| The details of the quality assessment were not shown | |

**Supplementary 6.4.**

| Pan 2013a ^16^ | | | | | | |
| --- | --- | --- | --- | --- | --- | --- |
| Quality assessment tool | Sanderson et al ^(1)^ | | | | | |
| Study | Selecting Study Participants | Measuring Exposure (Myopia) | Measuring Outcome (Cataract) | Design-Specific  Sources of Bias | Controlling Confounding, and Statistical Methods | Conflict of Interest |
| Giuffre (2005) | Using random numbers, a stratified random sampling was carried out | Not reported | Lens Opacities  Classification  System II | Selection bias; misclassification bias; chance finding; residual confounding; small sample size | Multivariate analysis adjusted for confounders: Nuclear cataract: iris atrophy; Cortical cataract: none; PSC cataract: iris atrophy, family history | None reported |
| Lim (1999) | Total of 3654 were selected from 4433 eligible residents in 2 postcode areas in the Blue Mountains area | Subjective refraction | Wisconsin Grading System | Selection bias; misclassification bias; chance finding; residual confounding | Multivariate analysis adjusted for age, sex, smoking, hypertension, diabetes, use of oral or inhaled steroids, and sun-related skin damage | None reported |
| Wong (2001) | All 5924 people who were 43-84 years of age were invited for baseline examinations. Of eligible persons, 4926 participated in the baseline examination | Subjective refraction | Wisconsin Grading System | Selection bias; misclassification bias; chance finding; residual confounding | Multivariate analysis adjusted for age, sex, diabetes, smoking, and education | None reported |
| Duan (2013) | Residents of Yongnian County in China aged 30+ years were randomly selected using a clustered sampling technique with probabilities proportionate to the size of population in each cluster | Subjective refraction | Lens Opacities  Classification  System III | Selection bias; misclassification bias; chance finding; residual confounding | Multivariate analysis adjusted for age, sex, fasting glucose, serum cholesterol, diabetes, body mass index, smoking, drinking, hypertension, time spent outdoors, level of income and education | None reported |
| Chang (2005) | Total of 2520 individuals aged 65 to 84 years, identified through Medicare rolls, who resided in the Salisbury area of the eastern shore of Maryland, were enrolled | Subjective refraction | Wilmer Grading System | Selection bias; misclassification bias; chance finding; residual confounding | Multivariate analysis adjusted for age, race, and sex.  Additionally adjusted: nuclear cataract: smoking, education; cortical cataract: diabetes, ultraviolet-B exposure; PSC cataract: diabetes | None reported |
| Wong (2003) | The electoral register listed 15082 names of Chinese aged between 40 and 79 years residing in Tanjong Pagar district, Singapore. Two thousand names (13.3%) were initially selected by a stratified, clustered, random sampling method, with more weights given to the older age groups | Subjective refraction | Lens Opacities  Classification  System III | Selection bias; misclassification bias; chance finding; residual confounding; small sample size | Multivariate analysis adjusted for age, sex, diabetes, smoking, and education | None reported |
| Pan (2013a) | An age-stratified random sample of the Indian population aged over 40 years was drawn from a computer-generated random list of 12 000 Indians living in southwest Singapore. In total, 3400 individuals (75.6%) participated in the study | Subjective refraction | Lens Opacities  Classification  System III | Selection bias; misclassification bias; chance finding; residual confounding | Multivariate analysis adjusted for age, sex, diabetes, smoking, and education | None reported |
| Pan (2013b) | An age-stratified random sample of the Malay population aged over 40 years was drawn from a computer-generated random list of 16 069 Malays living in southwest Singapore; 3280 individuals (78.7%) participated in the study | Subjective refraction | Wisconsin Grading System | Selection bias; misclassification bias; chance finding; residual confounding | Multivariate analysis adjusted for age, sex, body mass index, systolic blood pressure, HbA1c, smoking, and education | None reported |
| Wong (2001) | All subjects identified during the initial census were invited for a second examination 5 years after the first. Of the 4541 participants from the baseline examination surviving, 3684 (81.1%) returned for the follow-up examination | Subjective refraction | Wisconsin Grading System | Lost-to-follow-up bias; survival bias; chance finding; residual confounding | Multivariate analysis adjusted for age, sex, diabetes, smoking, and education | None reported |
| Younan (2002) | Five-year follow-up examinations were conducted, 2334 of the survivors (75.1%) were reexamined. Of those not seen, 383 (12.3%) had moved from the area and 394 (12.7%) refused the examination. | Subjective refraction | Wisconsin Grading System | Lost-to-follow-up bias; survival bias; chance finding; residual confounding | Multivariate analysis adjusted for age, sex, diabetes, smoking, and education | None reported |
| Mukesh (2006) | Baseline assessment was conducted on 3271 residents, recruited by door-to-door survey from 9 randomly selected adjacent pairs of census collector districts within urban Victoria. Five-year follow-up assessment was conducted on all available participants. | Subjective refraction | Wilmer Grading System | Lost-to-follow-up bias; survival bias; chance finding; residual confounding | Multivariate analysis adjusted for age, sex, country of birth, occupation, smoking, arthritis, diabetes, vitamin C and calcium channel  blockers intake | None reported |
| Leske (2002) | The studies were based on a simple random sample of Barbados African Americans, 40-84 years old (84% participation); 4631 persons completed baseline examinations at the study site. Surviving members of the cohort were invited to return for a 4-year follow-up visit | Subjective refraction | Lens Opacities  Classification  System II | Lost-to-follow-up bias; survival bias; chance finding; residual confounding | Multivariate analysis adjusted for age, sex, body mass index, iris color, diabetes, IOP, and IOP-lowering treatment | None reported |

**Supplementary 6.5.**

| Xiao 2017 ^17^ | | | | | |
| --- | --- | --- | --- | --- | --- |
| Quality assessment tool | de Weerd et al ^(2)^ and Rogers et al ^(3)^ | | | | |
| Study | Representing the general population | Appropriately recruiting the population | Adequate response rate (>70%) | Objective documentation of the outcomes | Total score |
| Duan (2009) | 1 | 1 | 1 | 1 | 4 |
| Ye (2015) | 1 | 1 | 1 | 1 | 4 |
| Kawasaki (2008) | 1 | 1 | 1 | 1 | 4 |
| McCarty (2005) | 1 | 1 | 1 | 1 | 4 |

**Supplementary 6.6.**

| Pan 2013b ^16^ | | | | | | |
| --- | --- | --- | --- | --- | --- | --- |
| Quality assessment tool | Sanderson et al ^(1)^ | | | | | |
| Study | Selecting Study Participants | Measuring Exposure (refractive errors) | Measuring Outcome (AMD) | Design-Specific  Sources of Bias | Controlling Confounding, and Statistical Methods | Conflict of Interest |
| Wang (1998) | 3654 (82.4%) were selected from 4433 eligible residents in two postcode areas in the Blue Mountains area, west of Sydney | Non-cycloplegic  subjective refraction | Wisconsin AMD Grading System | selection bias; misclassification bias; chance finding | Multiple logistic regression with the generalized estimating equation adjusted for age, gender, family history and smoking | None reported |
| Ikram (2003) * | Of the 10275 eligible adults aged 55 years or more in a suburb of Rotterdam, the Netherlands, 7983 participated in the study | Non-cycloplegic  subjective refraction | International AMD classification | selection bias; misclassification bias; chance finding | Logistic regression models adjusted for age and gender | None reported |
| Lavanya (2010) | An age-stratified random sample of the Malay population aged over 40 years was drawn from a computer-generated random list of 16069 Malays living in South-west Singapore. 3280 (78.7%) individuals participated in the study | Non-cycloplegic  subjective refraction | Wisconsin AMD Grading System | selection bias; misclassification bias; chance finding | Multiple logistic regression with the generalized estimating equation adjusted for age, sex, smoking, education, height, and systolic blood pressure | None reported |
| Jonas (2012) | Of 5885 eligible subjects in 8 villages in the rural region of India, 4711 (80.1%) people participated in the study | Non-cycloplegic  subjective refraction | Wisconsin AMD Grading System | selection bias; misclassification bias; chance finding | Logistic regression model adjusted for age and corneal refractive power | None reported |
| Cheung (2011) | Subjects aged 24-95 years were randomly selected from the Singapore population with disproportionate sampling stratified by racial groups | Non-cycloplegic Autorefraction | Wisconsin AMD Grading System | selection bias; misclassification bias; chance finding | Analyses were straitified by gender. Logistic regression model adjusted for age, race, smoking choronic kidney disease | None reported |
| Pan (2012) | An age-stratified random sample of the Indian population aged over 40 years was drawn from a computer-generated random list of 12000 Indians living in South-west Singapore. In total 3400 (75.6%) individuals participated in the study | Non-cycloplegic  subjective refraction | Wisconsin AMD Grading System | selection bias; misclassification bias; chance finding | Multiple logistic regression with the generalized estimating equation adjusted for age, gender, smoking, education, body mass index, hypertension and cholesterol level | None reported |
| Ikram (2003) ** | 4822 persons free of AMD at baseline and who participated at least in one follow-up examination were included | Non-cycloplegic  subjective refraction | International AMD classification | lost to follow up bias; survival bias | Logistic regression modeling was performed to establish the relationship of baseline refractive status with incident AMD correcting for age, gender, and follow-up time. | None reported |
| Wang (2004) | Five years later (1997–1999), 543 had died and 2335 of the 3111 surviving participants (75.1%) were re-examined in the follow-up visit | Non-cycloplegic  subjective refraction | Wisconsin AMD Grading System | lost to follow up bias; survival bias | Logistic regression models with the generalized estimating equation adjusted for age, sex, smoking | None reported |
| Wong (2002) | Of the 5924 people who were in the 43- to 84-year eligibility age range, 4926 participated in the baseline examination from 1988 through 1990. Of these, 2764 participated in the 10-year follow-up examination in 1998 through 2000 | Non-cycloplegic Autorefraction | Wisconsin AMD Grading System | lost to follow up bias; survival bias | Logistic regression models with the generalized estimating equation adjusted for age | None reported |

* Cross-sectional

** Cohort

**Supplementary 6.7.**

| Li 2014 ^19^ | |
| --- | --- |
| Quality assessment tool | Newcastle-Ottawa Scale |
| Study | NOS score |
| Jonas (2012) | 8 |
| Pan (2013) | 9 |
| Lavanya (2010) | 9 |
| Fraser Bell (2010) | 8 |
| Wang (1998) | 9 |
| Ikram (2003) * | 8 |
| Chaine (1998) | 7 |
| Cheung (2012) | 8 |
| McCarty (2001) | 6 |
| Erke (2012) | 7 |
| Wang (2004) | 8 |
| Wong (2002) | 8 |
| You (2012) | 6 |
| Buch (2005) | 6 |
| Ikram (2003) ** | 8 |

* Cross-sectional

** Cohort

**Supplementary 6.8.**

| Tang 2016 ^20^ | | | | | | | | | | | | | | |
| --- | --- | --- | --- | --- | --- | --- | --- | --- | --- | --- | --- | --- | --- | --- |
| Quality assessment tool | Modified Estabrooks’ Quality Assessment and Validity Tool | | | | | | | | | | | | | |
| Study | Probabilistic sample used | Representative | Sample size appropriate for power | Sample drawn > 1 site | Cluster/stratified design | Multiple adjusted | Response rate > 50% | DV directly measured/administrative | DV reliability | DV validity | Appropriate tests used | P values reported | CI reported | Missing data managed appropriately |
| Robaei (2006a) | Yes | Yes | Yes | Yes | Yes | N/A | Yes | Yes | N/A | Yes | Yes | Yes | Yes | N/A |
| Robaei (2006b) | Yes | Yes | Yes | Yes | Yes | Yes | Yes | Yes | N/A | Yes | Yes | Yes | Yes | N/A |
| Huynh (2006) | Yes | Yes | Yes | Yes | Yes | Yes | Yes | Yes | N/A | Yes | Yes | Yes | Yes | N/A |
| Cotter (2011) | Yes | Yes | Yes | Yes | Yes | Yes | Yes | Yes | N/A | Yes | Yes | Yes | Yes | N/A |
| Chia (2013) | Yes | Yes | Yes | Yes | Yes | Yes | Yes | Yes | N/A | Yes | Yes | Yes | Yes | N/A |
| Fu (2014) | Yes | Yes | Yes | Yes | Yes | N/A | Yes | Yes | N/A | Yes | Yes | Yes | Yes | N/A |
| Zhu (2015) | Yes | Yes | Yes | Yes | Yes | Yes | Yes | Yes | N/A | Yes | Yes | Yes | Yes | N/A |

**Supplementary 6.9.**

| Marcus 2011 ^21^ | |
| --- | --- |
| Quality assessment tool | Sanderson et al ^(1)^ |
| The details of the quality assessment were not shown | |

**Supplementary 6.10.**

| He 2018 ^22^ | |
| --- | --- |
| Quality assessment tool | Newcastle-Ottawa Scale |
| Study | NOS score |
| AI-Wadani (2014) | 7 |
| Avitabile (2008) | 8 |
| Lam (2008) | 8 |
| Li (2005) | 6 |
| Meng (2013) | 8 |
| Teke (2014) | 8 |

**Supplementary 6.11.**

| Guo 2015 ^23^ | |
| --- | --- |
| Quality assessment tool | Sanderson et al ^(1)^ |
| The details of the quality assessment were not shown | |

**Supplementary 6.12.**

| Xiong 2014 ^24^ | |
| --- | --- |
| Quality assessment tool | Not done |
| Xiang 2014 ^25^ | |
| Quality assessment tool | Not done |
| He 2021 ^26^ | |
| Quality assessment tool | Not done |
| Wang 2015 ^27^ | |
| Quality assessment tool | Not done |

1. Sanderson S, Tatt ID, Higgins JP. Tools for assessing quality and susceptibility to bias in observational studies in epidemiology: a systematic review and annotated bibliography. *Int J Epidemiol*. 2007;36:666-676.
2. de Weerd M, Greving JP, de Jong AW, et al. Prevalence of asymptomatic carotid artery stenosis according to age and sex: systematic review and metaregression analysis. *Stroke*. 2009;40:1105-1113.
3. Rogers S, McIntosh RL, Cheung N, et al. The prevalence of retinal vein occlusion: pooled data from population studies from the United States, Europe, Asia, and Australia. *Ophthalmology*. 2010;117:313-319.e311.
