# Supplementary material for: Refractive Error and Eye Health: An Umbrella Review of Meta-Analyses
Source: Front Med (Lausanne). 2021 Nov 4;8:759767. doi: 10.3389/fmed.2021.759767 (PMC8599990; doi:10.3389/fmed.2021.759767)

**Supplementary 7**

Supplementary 7.1. Fundus photo, OCT and visual field from a 42-year-old female with high myopia


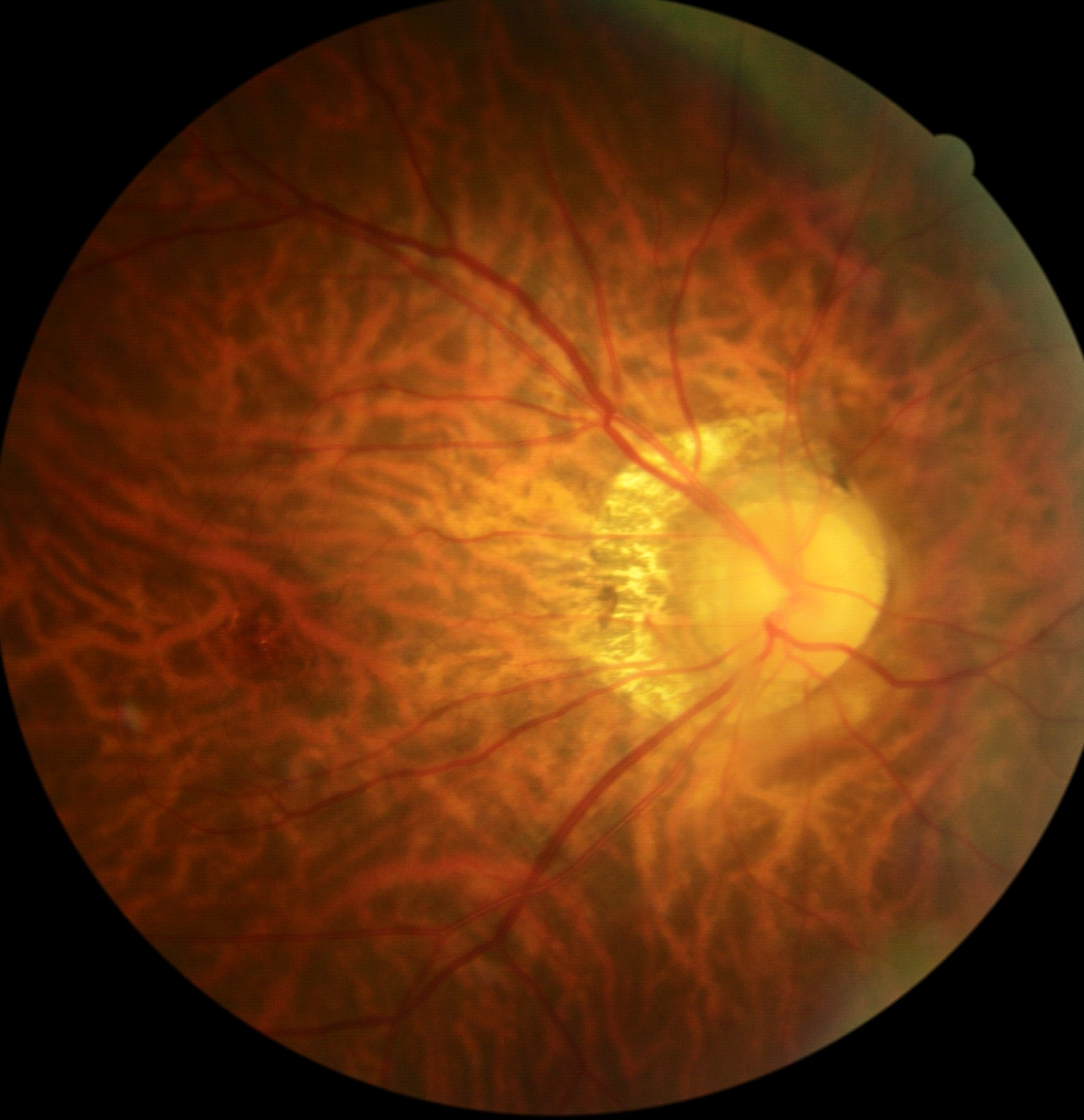


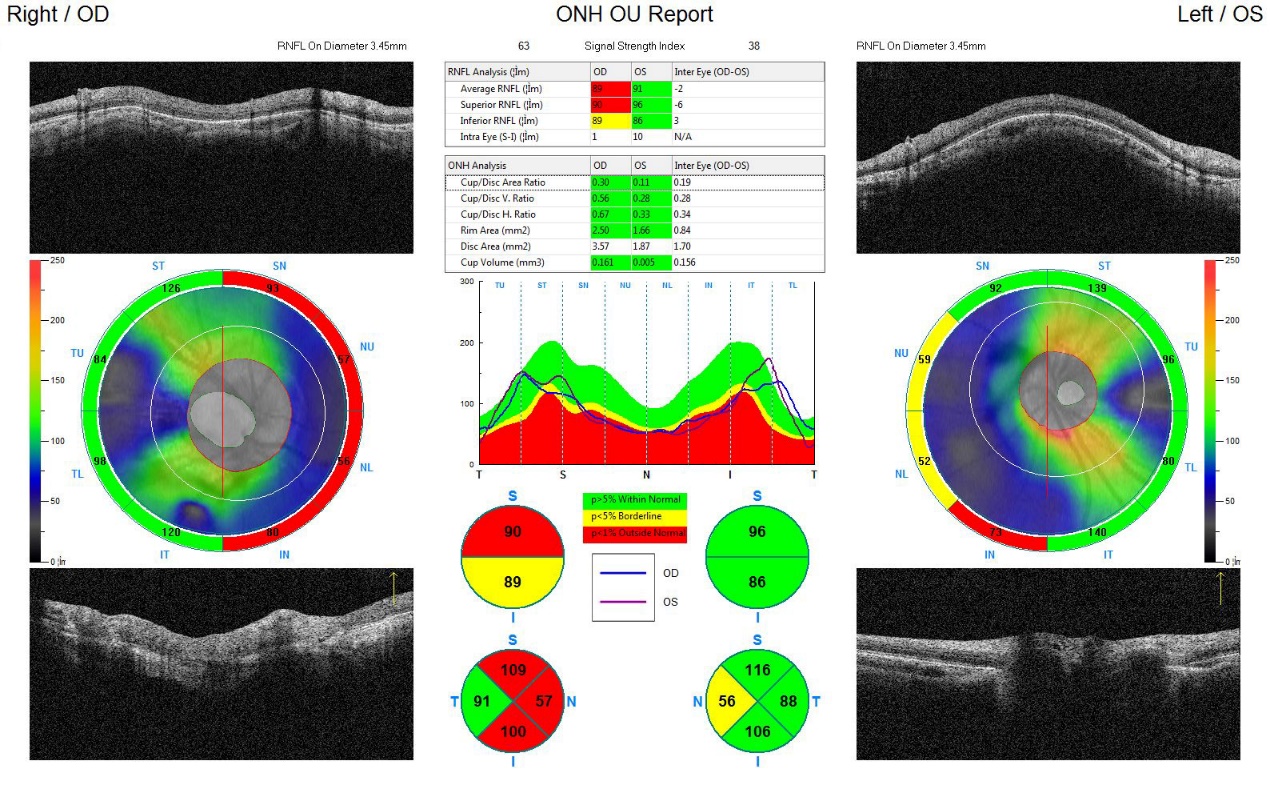


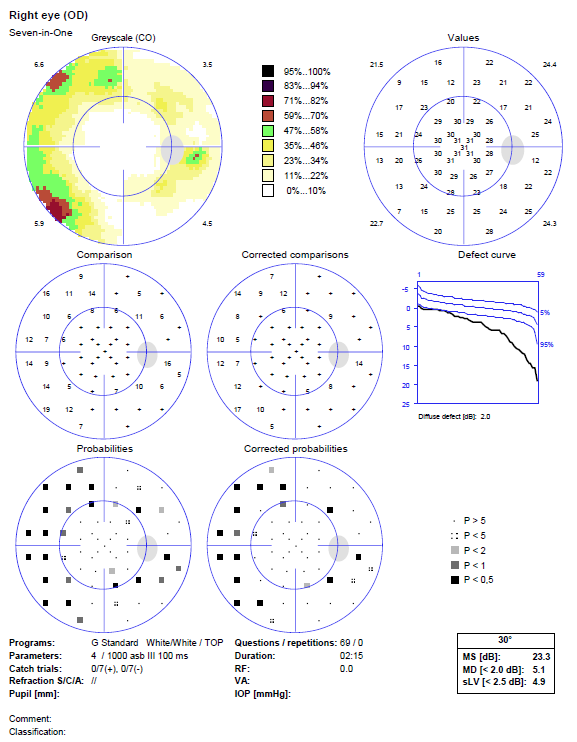


Supplementary 7.2. Fundus photo, OCT and visual field from a 36-year-old female with glaucoma and high myopia


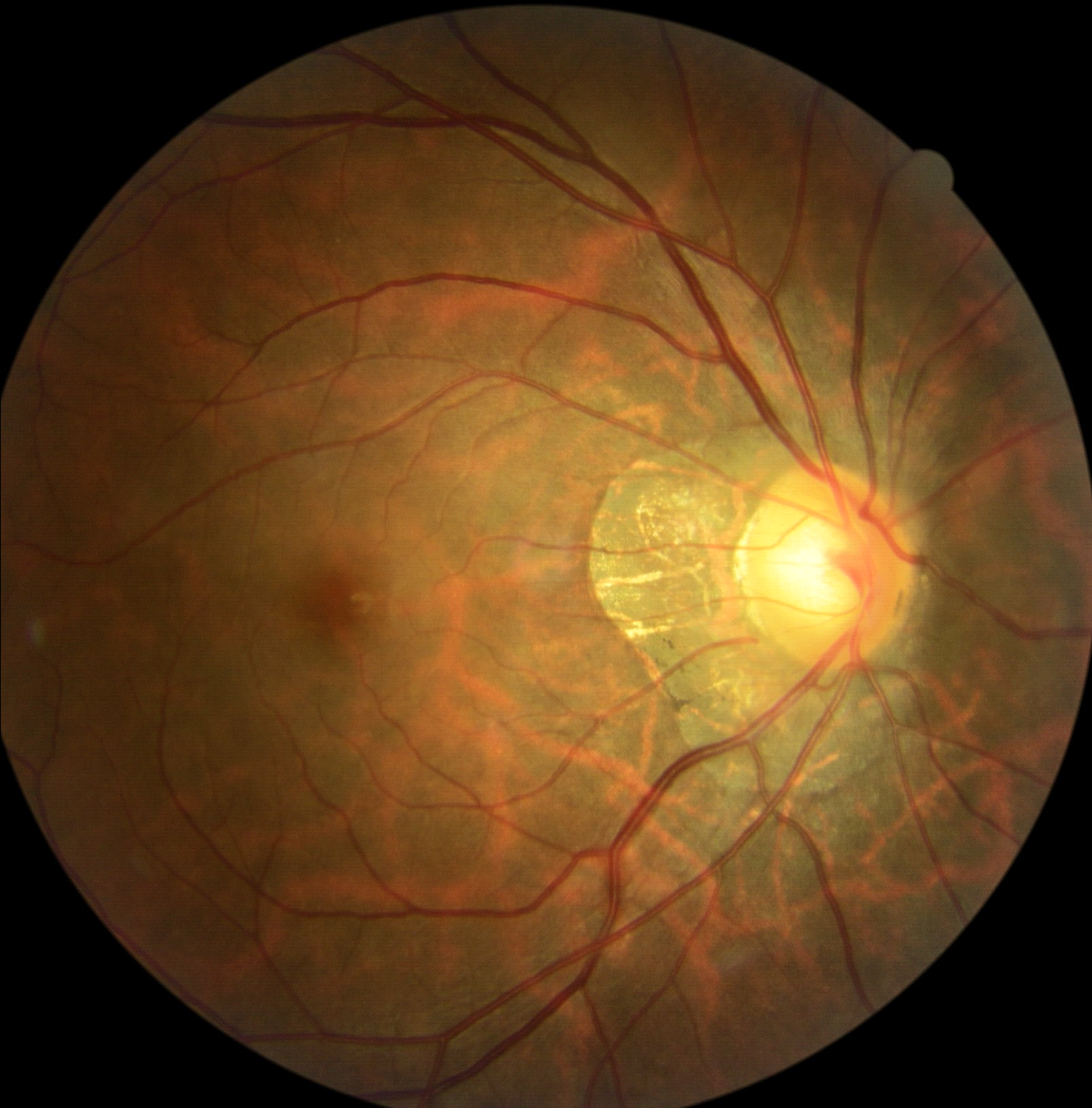


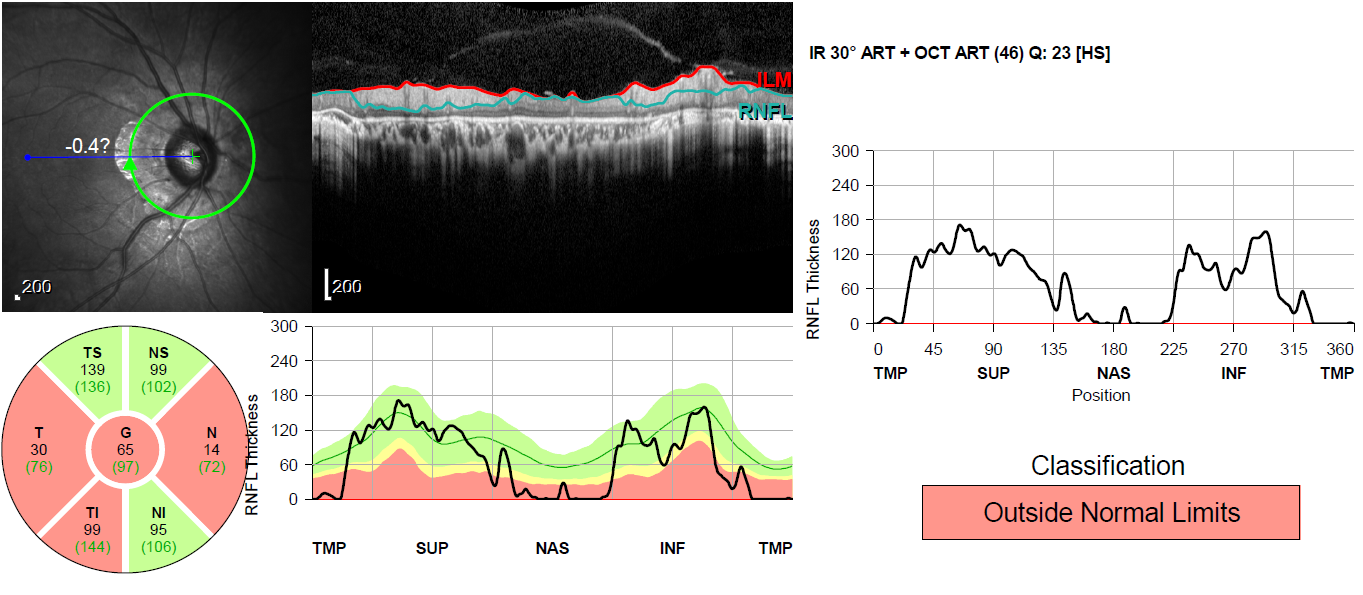


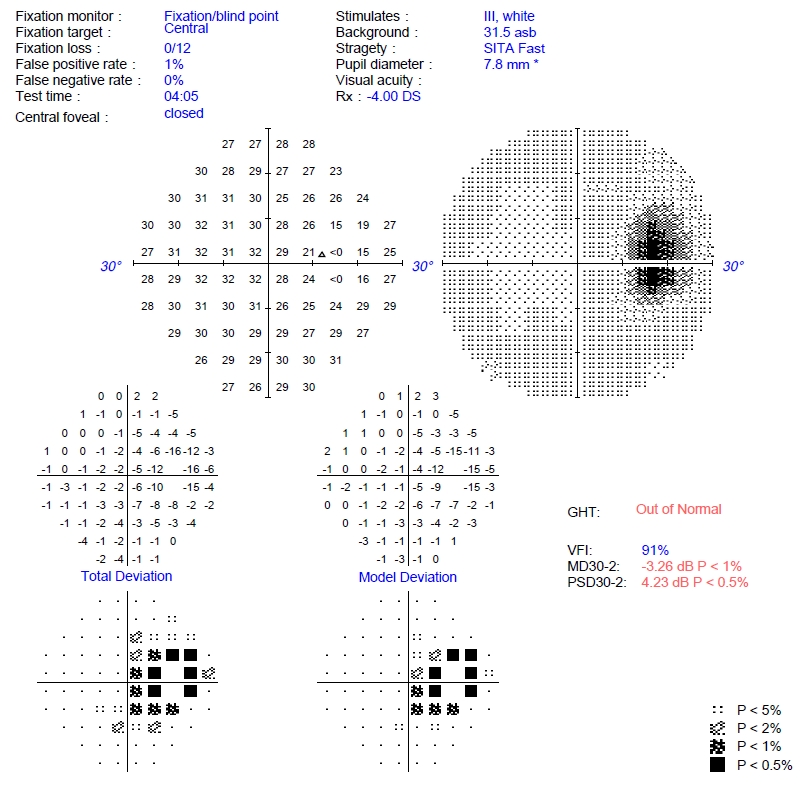

Supplement: Supplementary file 1 [file Data_Sheet_1.zip › 759767_Li_Supplementary7.docx]
